# The longitudinal care cascade for hypertension in people with and without HIV in South Africa: A clinic-based observational study

**DOI:** 10.1371/journal.pmed.1004957

**Published:** 2026-09-11

**Authors:** Siphamandla Bonga Gumede, Jennifer Manne-Goehler, Elizabeth Kelechi Oladimeji, Nicola Bulled, Alana T. Brennan, Samanta Tresha Lalla-Edward

**Affiliations:** 1 Ezintsha, Faculty of Health Sciences, University of the Witwatersrand, Johannesburg, South Africa; 2 Health Sciences Research Office (HSRO), Faculty of Health Sciences, University of the Witwatersrand, Johannesburg, South Africa; 3 Division of Infectious Diseases, Brigham and Women’s Hospital, Harvard Medical School, Boston, Massachusetts, United States of America; 4 MRC/Wits Rural Public Health and Health Transitions Research Unit, School of Public Health, University of the Witwatersrand, Johannesburg, South Africa; 5 Institute for Collaboration on Health, Intervention and Policy, University of Connecticut, Storrs, Connecticut, United States of America; 6 Department of Global Health, Boston University School of Public Health, Boston, Massachusetts, United States of America; 7 Department of Epidemiology, Boston University School of Public Health, Boston, Massachusetts, United States of America; 8 Health Economics and Epidemiology Research Office, Faculty of Health Sciences, University of the Witwatersrand, Johannesburg, South Africa; South African Medical Research Council, SOUTH AFRICA

## Abstract

**Background:**

Hypertension (HTN) constitutes a major and growing public health challenge in South Africa, a setting where HIV prevalence also remains high. Despite this dual burden, longitudinal evidence describing how individuals move through the HTN care cascade particularly comparing people living with HIV (PLHIV) and people not living with HIV (PNLHIV) remains limited. Understanding patterns of progression and regression across the cascade is essential to inform health system strategies for integrated chronic disease management.

**Methods and findings:**

We conducted a longitudinal secondary analysis using data collected from the iHEART-SA trial, implemented across nine public primary healthcare clinics in Johannesburg between August 2022 and May 2024, with follow-up through May 2025. Adults (≥18 years) receiving chronic care, with a known HIV status and a completed medical file review were included. Progression and regression were assessed across the HTN care cascade.

Of 23,855 participants, 78.5% were PLHIV (median age 42 years, IQR 36–49; 69% female). The median SBP was 132 mmHg (IQR: 120–145 mmHg). Along the HTN care cascade, PLHIV were substantially more likely to remain undiagnosed compared with PNLHIV (aRR 3.40; 95% [CI 2.45, 4.73]; *p* < 0.001). Among those treated, PLHIV were less likely to achieve BP control (aRR 0.85; 95% [CI 0.75, 0.97]; *p* = 0.012). During follow-up, PLHIV experienced higher rates of cascade regression, including regression to untreated HTN (22% versus 11%; aRR 1.51; 95% [CI 1.03, 2.23]; *p* = 0.037) and from controlled to uncontrolled BP (aRR 1.10; 95% [CI 1.02, 1.18]; *p* = 0.010).

The main study limitations were the use of routinely collected clinical data, variable follow-up, and treatment ascertainment based on documented medication use.

**Conclusions:**

PLHIV had lower HTN diagnosis and higher regression, including treatment discontinuation and loss of BP control, underscoring persistent gaps in longitudinal HTN management within HIV programmes and the need for integrated and targeted chronic care models that ensure treatment continuity and sustained control.

## Author summary

**Why was this study done?:**

- Hypertension is a leading cause of cardiovascular disease in South Africa and affects both people living with HIV and people not living with HIV.
- Although many studies have described the prevalence of hypertension, less is known about how people move over time through different stages of hypertension care, including diagnosis, treatment, and sustained blood pressure (BP) control.
- Understanding whether patterns of hypertension care differ between people living with and without HIV is important for identifying gaps in care and informing chronic disease management strategies.

**What did the researchers do and find?:**

- We analysed longitudinal data from 23,855 adults attending nine public primary healthcare clinics in Johannesburg, South Africa, between 2022 and 2025.
- We compared progression and regression across the hypertension care cascade between people living with HIV and people not living with HIV.
- More than one-third of participants had high BP. People living with HIV were more likely to remain undiagnosed, less likely to achieve BP control once treated, and more likely to move from treated or controlled states back to untreated or uncontrolled hypertension than people not living with HIV.

**What do these findings mean?:**

- Important gaps remain across multiple stages of hypertension care, including diagnosis, treatment continuity, and sustained BP control.
- People living with HIV experienced poorer longitudinal hypertension outcomes than people not living with HIV, despite receiving care within the same public primary healthcare system.
- Because this study used routine healthcare records and followed people for different lengths of time, it cannot fully explain why these differences occurred. Even so, the findings highlight the need to strengthen hypertension care within routine chronic disease services and to better understand why people move forward or backward along the hypertension care pathway.

## Introduction

Hypertension (HTN) is a growing public health challenge in middle-income countries (MICs) [1–3]. In South Africa, the prevalence of HTN is ~46% in the general population, with estimates suggesting similar or slightly lower rates among people living with HIV (PLHIV) compared to people not living with HIV (PNLHIV) [4–6]. The convergence of the HIV and HTN epidemics presents a significant challenge for the health system but also offers an opportunity to integrate chronic disease management. Several cross-sectional studies have suggested that HTN care may be more effective among PLHIV, largely due to more frequent clinical contact and better continuity of care through established HIV programmes [2,3,7–9]. The robust HIV care infrastructure facilitates systematic screening, earlier treatment initiation, and regular follow-up for comorbid conditions such as HTN. Consequently, PLHIV may have higher rates of diagnosis and treatment initiation for HTN compared to PNLHIV. The relationship between HIV status and longitudinal HTN care outcomes remains unclear. While engagement with HIV services may create opportunities for HTN screening and management, this does not necessarily translate into improved progression and retention across stages of the HTN care cascade.

Despite the feasibility of treating and controlling HTN within the South African healthcare system, current evidence suggests that effective blood pressure (BP) control remains suboptimal at the population level [10,11]. Cross-sectional data indicate that only about 10% of HTN individuals achieve control (often summarised as the “ABC”—Awareness, BP control, and Compliance) [11]. Sustaining this control over time is even more challenging; a longitudinal study from South Africa found that only around 51% of patients who initially achieved control maintained it at follow-up [12]. However, there is limited evidence on whether such patterns differ by HIV status, particularly over time.

To date, longitudinal data directly comparing progression through the HTN care cascade, including diagnosis, treatment, and sustained BP control between PLHIV and PNLHIV are lacking [11,13,14]. Generating such evidence is critical for informing health system interventions that leverage the strengths of existing HIV care platforms to improve cardiovascular outcomes more broadly. These insights are especially relevant in South Africa, where integrated chronic disease care models are being scaled up to address the dual burden of infectious and noncommunicable diseases (NCDs).

In this study, we use a large clinical dataset from the Integrating HIV and Heart Health in South Africa (iHEART-SA) trial to provide a comprehensive description and comparison of progression and regression across stages of the HTN care cascade among PLHIV and PNLHIV in Johannesburg, South Africa. We examined whether progression and regression across the HTN care cascade differed by HIV status.

## Methods

### Study setting

The iHEART-SA trial was conducted in 9 of 14 public sector clinics in region F (inner-city) of the City of Johannesburg, a district within the Gauteng province. These clinics serve almost 700,000 residents in total and offer reproductive and sexual healthcare, mobile and community outreach, HIV screening and treatment, and tuberculosis screening and treatment. As the economic centre of the country, Johannesburg has a large immigrant population, including both South Africans from different provinces within the country and individuals from other countries.

### Data source

This longitudinal study was conducted as a secondary analysis of data collected through the iHEART-SA trial, an implementation study conducted across nine public primary healthcare clinics in Johannesburg, South Africa. The prespecified hypothesis was that patterns of progression and regression across the HTN care cascade would differ by HIV status, with HIV status being the primary exposure of interest. The study objectives, HTN care cascade outcomes, and analytical approach were specified in a written secondary analysis protocol developed prior to conducting the analyses (S1 Protocol). The parent study enrolled adults aged ≥18 years attending participating clinics, irrespective of HIV status or BP level. This study is reported in accordance with the Strengthening the Reporting of Observational Studies in Epidemiology (STROBE) guideline (S1 Checklist).

In this secondary analysis, participants were subsequently classified across stages of the HTN care cascade using BP measurements, diagnosis status, and treatment information obtained through routine medical record review. The present analysis included both PLHIV and PNLHIV to enable comparative assessment of progression and regression across stages of the HTN care cascade.

Data were extracted for participants enrolled between August 2022 and May 2024, with follow-up through routine medical record review extending to May 2025. Follow-up time therefore varied by enrolment date, with some participants contributing up to ~3 years of observation and others ~12 months. This study period allowed assessment of longitudinal transitions across the HTN care cascade within the overall 24-month implementation window and subsequent follow-up period. Participants with baseline data and a completed medical record review were included in the descriptive analyses. Longitudinal progression and regression analyses were subsequently conducted among participants eligible for each transition outcome, based on their baseline HTN care cascade stage and the availability of follow-up clinical information. Consequently, denominators differed across the longitudinal analyses.

The predominance of PLHIV in the study population reflects routine service utilisation patterns within participating primary healthcare clinics, where HIV-related services constitute a substantial proportion of chronic care visits, rather than sampling intended to achieve balanced representation by HIV status. Therefore, the study population is not representative of the general population. Additional details regarding the iHEART-SA trial design and implementation procedures have been described elsewhere. The parent iHEART-SA trial received ethical approval from the Human Research Ethics Committee (Medical) of the University of the Witwatersrand (M211160), and the present secondary analysis was approved by the same committee (M2411128) [15].

### Defining longitudinal HTN care cascade

HTN was defined using routinely documented BP measurements, documented HTN diagnosis, or antihypertensive treatment recorded in the medical record at the relevant time point. In practice, routine medical records generally contained only a single BP reading per visit, and information on whether repeat confirmatory measurements were obtained was not consistently available. Consequently, HTN classification in this analysis was based on single documented BP measurements in combination with diagnosis and treatment documentation recorded during routine care. Although clinical guidelines generally recommend confirmation using repeated BP measurements, this was not possible within the available routine data. BP categories were defined as follows: normal BP: systolic BP (SBP) 120–129 mmHg or diastolic BP (DBP) 80–85 mmHg; high-normal BP: SBP 130–139 mmHg or DBP 86–89 mmHg; and high BP/HTN: SBP of ≥140 mmHg or DBP of ≥90 mmHg [16].

Antihypertensive treatment was defined based on medications documented in the medical record at the relevant time point. Medication information was collected using predefined medication fields in the iHEART-SA file review instrument, which captured antihypertensive agents routinely prescribed within South African public sector primary healthcare services during the study period, using five pre-specified medications (hydrochlorothiazide, enalapril, amlodipine, furosemide, and atenolol; ATC codes provided in S1 Table). Medications recorded outside this list were captured via free-text fields and reviewed in real time by a clinician or nurse on the study team, who cross-checked each entry against the patient’s documented diagnosis/indication in the medical record to confirm hypertension-related use prior to classification as antihypertensive treatment (S1 Table and S1 Appendix). Treatment status at baseline was determined using medication information recorded at the enrolment visit and used to classify participants into initial cascade stages. During follow-up, treatment status was reassessed at each available visit based on recorded medication use. As detailed prescription duration data were not available, treatment status reflects documented medication use at each visit rather than inferred continuity between visits. Treatment status was treated as a time-varying variable and could change across visits based on clinical documentation.

For this longitudinal analysis, baseline was defined as the participant’s enrolment visit into the iHEART-SA study, at which BP measurements, HTN diagnosis status, and antihypertensive treatment information were recorded from routine clinical records. Follow-up consisted of all subsequent documented clinic visits available through medical record review until May 2025. Participants contributed to specific progression and regression analyses according to their baseline HTN care cascade stage, and denominators therefore differed across transition outcomes. Because participants could experience multiple changes in HTN care status over time, an individual could contribute to more than one longitudinal transition outcome during follow-up

Progression and regression across the HTN care cascade were assessed longitudinally during follow-up. These outcomes were defined based on any observed change in HTN care cascade status during available follow-up visits rather than status at the final recorded visit. Participants were classified as having experienced a progression or regression outcome if the relevant transition was documented at any point during routine follow-up care. Consequently, participants could contribute to both progression and regression outcomes over time if multiple changes across cascade stages were observed during follow-up.

**Primary outcome:** Estimates were reported for each stage and outcome (undiagnosed, diagnosed but untreated, diagnosed and treated, treated and controlled, regressed to untreated, and regressed to uncontrolled) based on the primary exposure variable (HIV status) and covariates of interest (Table 1).

**Table 1:** Definitions of the six hypertension care cascade stages.

Stage	Definition	Denominator
**Baseline status** *(assessed at enrolment/initial visit)*		
**1. Undiagnosed**	**Baseline status** among participants with high BP at the initial/enrolment visit. Classified as undiagnosed if high BP was present at baseline with no prior documentation of a hypertension diagnosis.	All participants with high BP at baseline.
**2. Diagnosed, untreated**	**Baseline status** among participants with a documented hypertension diagnosis but no evidence of antihypertensive treatment initiation at baseline.	All participants with a hypertension diagnosis at baseline.
**3. Diagnosed and treated**	**Baseline status** among participants with a documented hypertension diagnosis who had initiated antihypertensive therapy at baseline. **Note:** *Includes participants classified as "ever treated" at baseline.*	All participants with a hypertension diagnosis at baseline.
*Note: Participants could enter the cohort at any stage of the HTN care cascade at baseline, depending on their BP measurements, diagnosis status, and treatment documentation at the enrolment visit.*		
**Longitudinal outcomes** *(assessed at follow-up)*		
**4. Treated and controlled**	**Progression outcome** among participants on antihypertensive treatment at baseline who achieved BP control at follow-up.	All participants on antihypertensive treatment at baseline, regardless of subsequent regression.
**5. Regressed to untreated**	**Regression outcome** among participants on antihypertensive treatment at baseline who were no longer on treatment at follow-up.	All participants on antihypertensive treatment at baseline.
**6. Regressed to uncontrolled**	**Regression outcome** among participants who were treated and controlled at baseline but had uncontrolled BP at follow-up.	All participants who were treated and controlled at baseline.

**Legend:** This table defines the six stages of the longitudinal HTN care cascade used in the study and the corresponding denominator for each stage. Baseline stages were assigned using BP measurements, HTN diagnosis status, and antihypertensive treatment documented at the enrolment visit. Progression and regression stages were assessed longitudinally during follow-up based on documented changes in HTN care status. Participants contributed to progression or regression analyses according to their baseline HTN care cascade stage; consequently, denominators differed across transition outcomes.

**Abbreviations:** HTN, hypertension; BP, blood pressure.

“Progression” was defined as movement of individuals from their baseline stage to a more advanced stage of the HTN care cascade at any subsequent time point during follow-up. For example, a participant could progress from diagnosis to treatment initiation, or from treatment to BP control. Once BP control was achieved, no further forward progression was possible along the continuum [12].

“Regression” was defined as movement from a more advanced to a prior stage of the HTN care cascade over time, such as loss of BP control or discontinuation of treatment. Because participants were assessed at multiple timepoints, individuals could experience both progression and regression during follow-up. Each change was evaluated sequentially according to the direction of movement between consecutive stages. Once a participant was diagnosed with HTN, they could no longer be considered undiagnosed [12].

**Fig 1:**
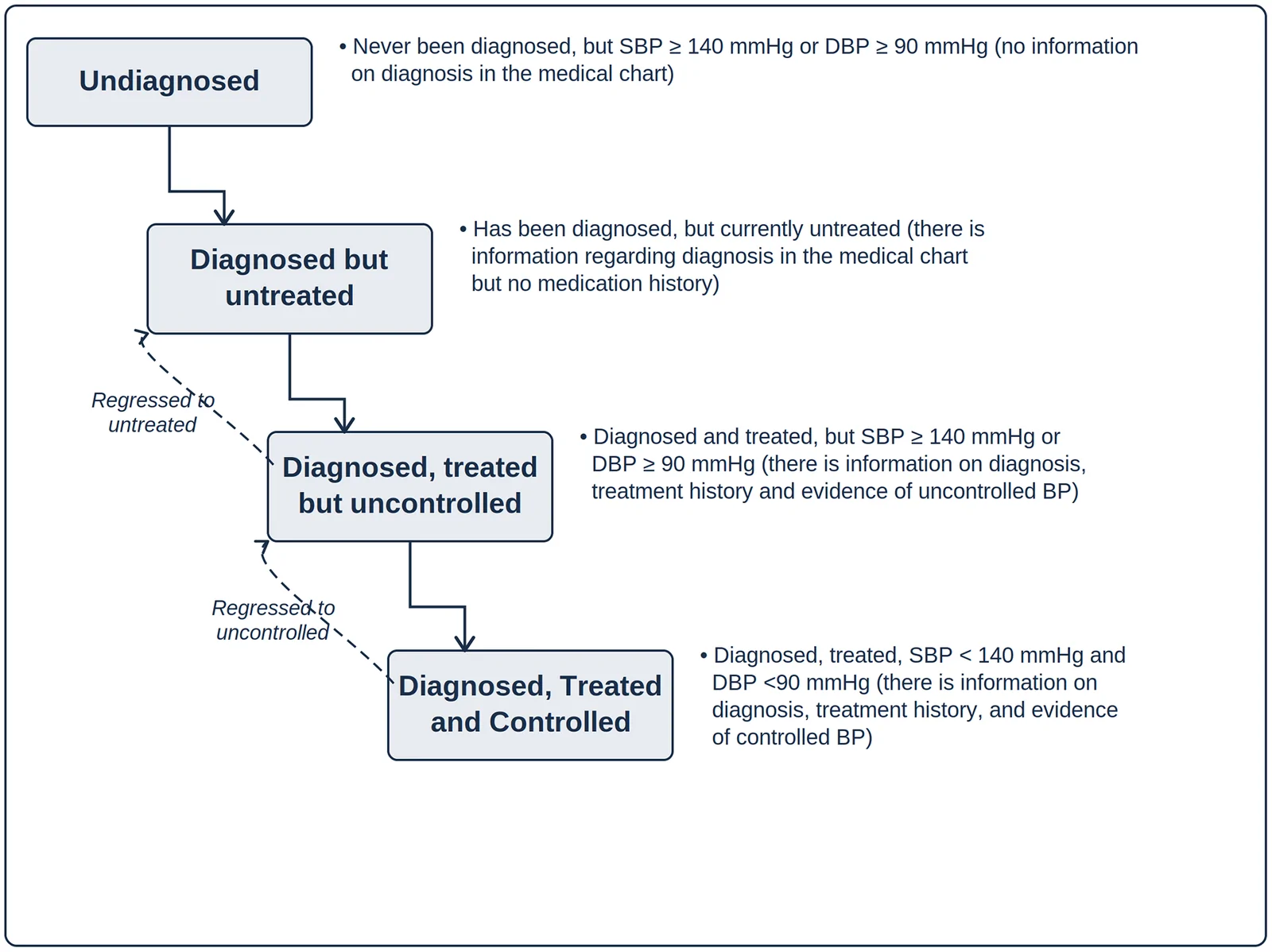
Stages of the HTN care cascade for the study population. **Legend:** The figure illustrates the HTN care cascade states used to classify participants with high blood pressure (BP) during longitudinal follow-up. Participants were categorised as undiagnosed, diagnosed but untreated, diagnosed and treated but uncontrolled, or diagnosed, treated and controlled based on information recorded in routine medical records, including hypertension diagnosis, antihypertensive treatment, and BP measurements. Progression was defined as movement to a subsequent stage of the cascade, while regression was defined as movement from a treated state to either untreated hypertension or uncontrolled hypertension during follow-up. **Abbreviations:** HTN, hypertension; SBP, systolic blood pressure; DBP, diastolic blood pressure.

At baseline, participants were classified into mutually exclusive stages of the HTN care cascade based on BP measurements, diagnosis status, and treatment documentation (Table 1 and Fig 1):

Figure 1 describes individuals’ transition through the HTN care cascade during the study period by reporting the progression and regression of HTN care across all clinical transitions.

### Sample size, inclusion, and exclusion criteria

Adults aged ≥18 years with a known HIV status who consented to participate in the iHEART-SA trial and had a completed medical file review were eligible for inclusion in this secondary analysis. Participants with missing HIV status or without completed medical record review data were excluded. S1 Figure presents the participant flow diagram and application of inclusion criteria.[15].

### Sociodemographic covariates

The following data were available on sociodemographic covariates: age (categorised as: 18–39, 40–49, 50–59, and 60+ in the regression model), sex (male or female), and nationality (South African, Zimbabwean, Other).

### Statistical analyses

We reported simple descriptive statistics, frequencies, proportions, and/or medians with interquartile ranges (IQR) for the covariates included in the analyses (sex, age, and nationality). We also reported the frequencies and proportions of individuals progressing and regressing across stages of the HTN care cascade during follow-up. For each outcome, unadjusted risk ratios (RRs) and 95% confidence intervals (CIs) were estimated using univariable Poisson regression with robust variance and clinic-level clustering, with each covariate considered separately. Adjusted risk ratios (aRRs) and 95% CIs were estimated using multivariable Poisson regression models including HIV status, age category, sex, and nationality, with robust variance and clinic-level clustering. These prespecified covariates were selected based on their potential relevance to HTN care and HIV status and were included in all multivariable models. The aRR for each covariate therefore represents its association with the outcome after adjustment for the other covariates in the model. Analyses for each cascade outcome were restricted to participants eligible for the relevant transition stage and with available follow-up data for assessment of that transition. Consequently, the denominator varied across longitudinal outcomes according to baseline cascade stage, follow-up availability, and completeness of outcome-specific data. This approach was selected because several outcomes were common, and RRs were considered more interpretable than odds ratios for the study objectives [16]. Because the primary objective was to characterise observed progression and regression across the HTN care cascade during available follow-up rather than estimate standardised transition rates over fixed time intervals, analyses focussed on whether transitions occurred during follow-up irrespective of exact observation duration. Analyses were conducted using complete-case data for variables included in each model. Consequently, denominators varied slightly across regression analyses because participants with missing data for specific covariates or outcome classifications were excluded from the relevant model. The analyses presented in this manuscript were prespecified as part of the secondary analysis objectives and were conducted as described in the methods. No additional post hoc or data-driven analyses were undertaken.

### Artificial intelligence (AI) tools and technologies

OpenAI ChatGPT (GPT-5.5) was used to assist with language editing, improving clarity, and formatting. Claude (Anthropic; Claude Sonnet 4.6) was used to cross-check the consistency of data against the study’s Stata dofile script. AI tools were not used for study design, data collection, data management, statistical analyses, interpretation of the findings, or generation or modification of the study results. All AI-assisted content was critically reviewed, verified against the original analyses and source materials, and revised by the authors as necessary. The authors retain full responsibility for the accuracy, integrity, and final content of the manuscript.

## Results

Between August 2022 and May 2024, 37,620 individuals were screened for iHEART-SA trial, of whom 33,484/37,620 (89.0%) had known HIV status. After applying eligibility criteria and obtaining consent, 23,855/25,120 (95.0%) participants had a completed medical file review (S1 Fig).

### Sample characteristics

Table 2 provides descriptive characteristics for the study participants. Of the 23,855 participants, 78.5% (18,720/23,855) were PLHIV. Of the total, 15,553 (65.2%) participants had more than one visit during the study period, including 12,174 (65.0%) PLHIV and 3,379 (65.8%) PNLHIV. Participants attended a median of 2 documented clinic visits (IQR 1–4) including the initial visit, with PNLHIV contributing a higher median number of visits than PLHIV (3 [IQR 1–5] versus 2 [IQR 1–4], respectively). The median follow-up duration was 337 days (IQR: 168–532) overall, 336 days (IQR: 168–525) among PLHIV, and 358 days (IQR: 169–559.5) among PNLHIV.

**Table 2:** Descriptive characteristics for the study participants.

Participant characteristics	All	PLHIV	PNLHIV
**Participants who had their medical records reviewed**	**23,855**	**18,720**	**5,135**
**Median follow-up duration in days (IQR)**	337 (168–532)	336 (168–525)	358 (169–559.5)
**Mean follow-up duration in days (SD)**	376 (231)	370 (229)	395 (234)
**Had ≥1 follow-up visit, n (%)**	15,553 (65.2%)	12,174 (65.0%)	3,379 (65.8%)
**Number of documented clinic visits incl. initial visit, median (IQR)**	2 (1–4)	2 (1–4)	3 (1–5)
**Number of documented clinic visits incl. initial visit, mean (SD)**	2.8 (2.3)	2.7 (2.1)	3.5 (2.7)
**Median age (IQR, in years)**	44 (37–52)	42 (36–49)	51 (42–61)
**Age category (in years)**			
18–39	7,225 (31.42%)	6,383 (35.50%)	842 (16.78%)
40–49	8,587 (37.34%)	7,268 (40.42%)	1,319 (26.29%)
50–59	4,946 (21.51%)	3,518 (19.57%)	1,428 (28.46%)
60+	2,240 (9.74%)	811 (4.61%)	1,429 (28.48%)
**Sex**			
Female	16,296 (68.31%)	12,940 (69.12%)	3,356 (65.36%)
Male	7,559 (31.69%)	5,780 (30.88%)	1,779 (34.64%)
**Nationality**			
South African	16,097 (67.48%)	12,380 (66.13%)	3,717 (72.39%)
Zimbabwean	5,825 (24.42%)	5,023 (26.83%)	802 (15.62%)
Other	1,933 (8.10%)	1,317 (7.04%)	616 (12.00%)
Median SBP (mmHg) for those who underwent file review	132 (120–145)	129 (117–141)	140 (129–154)
Median DBP (mmHg) for those who underwent file review	81 (72–90)	80 (71–89)	84 (75–93)

**Legend:** Characteristics are presented for all participants and stratified by HIV status. Categorical variables are reported as *n* (%), and continuous variables are presented as median (interquartile range [IQR]) or mean (standard deviation [SD]), as appropriate. Percentages are calculated using participants with available data for each variable; therefore, denominators may vary slightly because of missing values.

**Abbreviations:** PLHIV, People living with HIV; PNLHIV, People not living with HIV; SD, Standard Deviation; IQR, Interquartile Range; SBP, Systolic Blood Pressure; DBP, Diastolic Blood Pressure; mmHg, millimetres of mercury.

The median age was 44 years (IQR: 37–52), with PLHIV being younger (median 42 years, IQR: 36–49) compared to PNLHIV (median 51 years, IQR: 42–61). Of the total participants, 68.3% (16,296/23,855) were female, with a slightly higher proportion among PLHIV (69.1%, 12,940/18,720) than PNLHIV (65.4%, 3,356/5,135). South Africans comprised 67.5% (16,097/23,855) of participants, more among PNLHIV (72.4%, 3,717/5,135) compared to PLHIV (66.1%, 12,380/18,720). One-quarter of the participants were Zimbabweans (24.4%, 5,825/23,855), with a higher proportion among PLHIV (26.8%, 5,023/18,720) versus PNLHIV (15.6%, 802/5,135). The median SBP was 132 mmHg (IQR:120–145 mmHg).

### Movement in the longitudinal HTN care cascade

Results reflect changes in HTN care cascade among participants enrolled in the iHEART-SA trial between August 2022 and May 2024 and followed up through May 2025 using routine medical record data. Progression and regression along the HTN care cascade were therefore assessed for each participant, within the 24-month implementation window and the 12 months follow-up period.

### Progression of individuals across the longitudinal HTN care cascade

**Fig 2:**
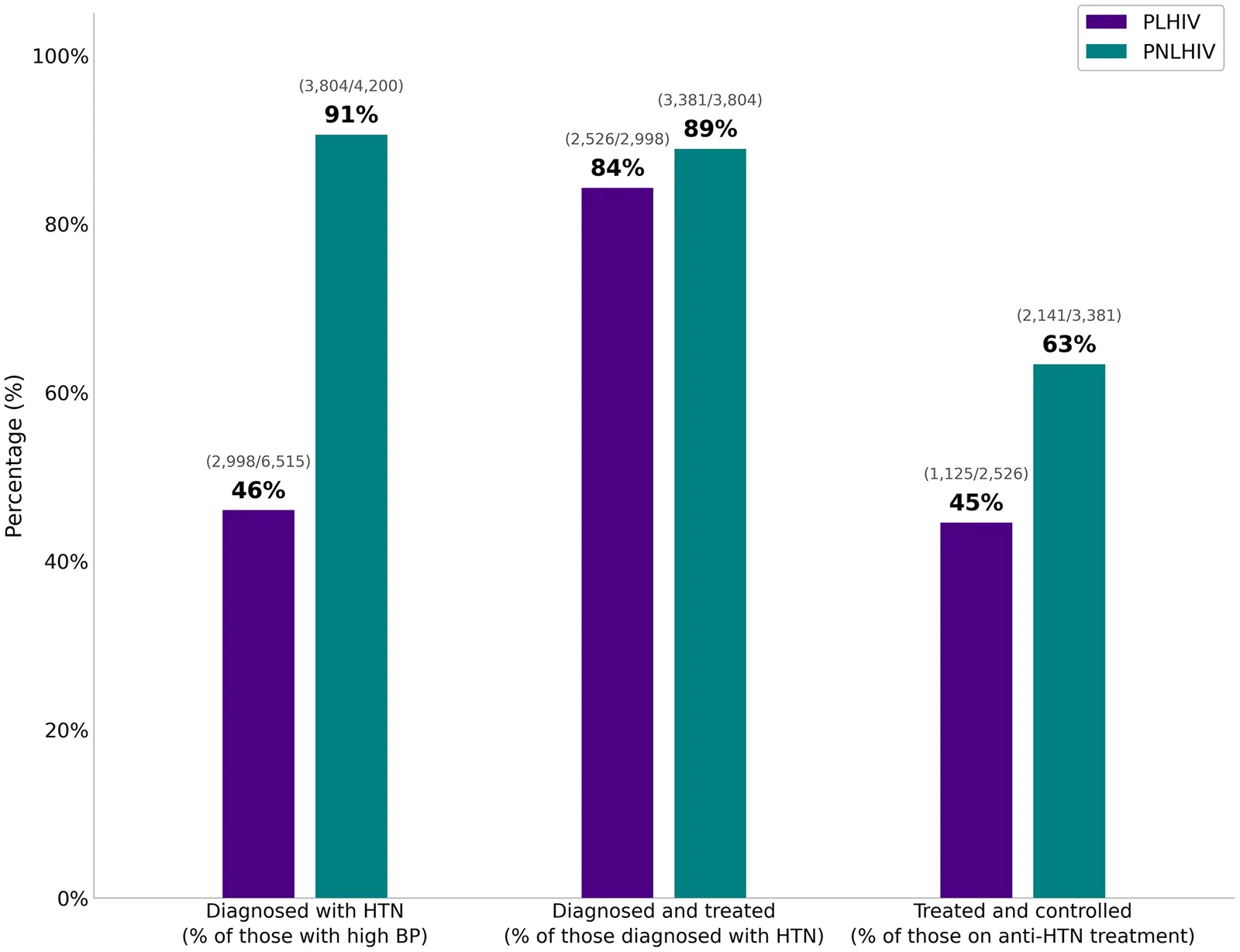
Progression in HTN care cascade among participants enrolled Aug 2022–May 2024 and followed through May 2025. **Legend:** The figure shows the proportion of participants at each stage of the HTN care cascade at enrolment, stratified by HIV status, and the proportion achieving BP control at follow-up among those receiving antihypertensive treatment at enrolment. Participants were classified according to their documented BP, HTN diagnosis, and antihypertensive treatment status at enrolment. The first stage shows the proportion of participants with high BP at enrolment; subsequent stages show HTN diagnosis and antihypertensive treatment among participants meeting the corresponding criteria. The final stage shows BP control at follow-up among participants who were receiving antihypertensive treatment at enrolment. Percentages and corresponding numerators/denominators are shown for each stage. **Abbreviations:** PLHIV, people living with HIV; PNLHIV, people not living with HIV; HTN, hypertension; BP, blood pressure.

Figure 2 illustrates the forward movement of participants through the stages of HTN care. Of the total PLHIV with high BP at enrolment, 46% (2,998/6,515) were diagnosed with HTN, compared to 91% (3,804/4,200) of PNLHIV. Among those diagnosed, 84% (2,526/2,998) of PLHIV progressed to anti-HTN treatment, compared to 89% of PNLHIV (3,381/3,804). Among individuals who began treatment, 45% (1,125/2,526) of PLHIV achieved BP control, compared to 63% (2,141/3,381) of PNLHIV, highlighting a significant drop-off at the control stage for both groups, but substantially worse for PLHIV.

### Regression of individuals across the longitudinal HTN care cascade

**Fig 3:**
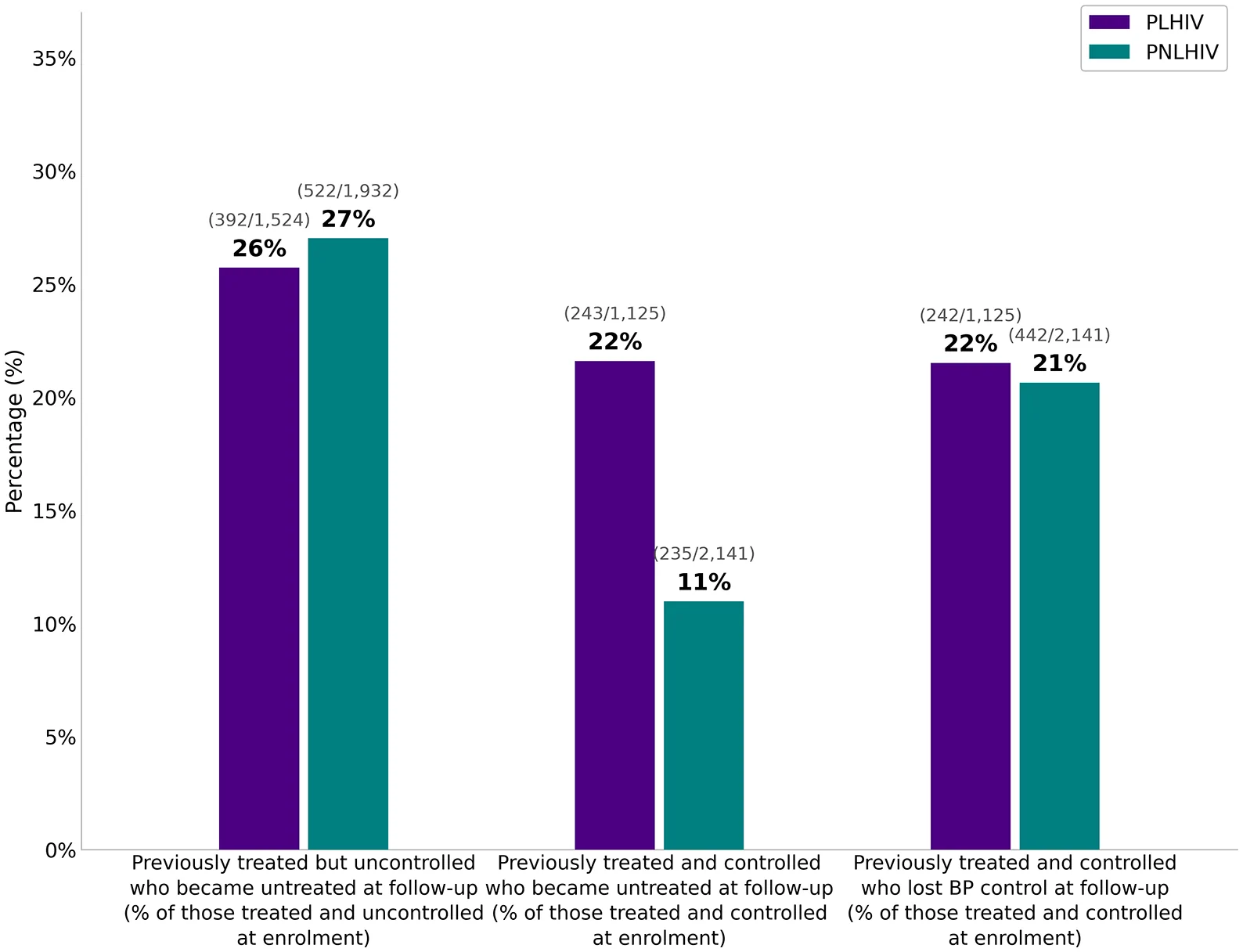
Regression in HTN care cascade among participants enrolled Aug 2022–May 2024 and followed through May 2025. **Legend:** The figure shows the proportion of participants who experienced regression in HTN care status during follow-up, stratified by HIV status. Participants were classified according to their documented HTN care status at enrolment and followed longitudinally using available information from subsequent clinical visits. Participants entered follow-up with their documented baseline HTN care status. Percentages and corresponding numerators/denominators are shown for each regression outcome. **Abbreviations:** PLHIV, people living with HIV; PNLHIV, people not living with HIV; BP, blood pressure.

Figure 3 presents the regression or backward movement of participants across the cascade. Of those who had been previously diagnosed with HTN and had initiated antihypertensive treatment but remained uncontrolled, almost one in four discontinued treatment and regressed to being untreated at follow-up visits (26%, 392/1524 for PLHIV versus 27%, 522/1932 for PNLHIV).

Of those who initially achieved BP control, 22% (243/1125) of PLHIV versus 11% (235/2141) of PNLHIV, regressed to being untreated at follow-up visits. Furthermore, almost one in five of the participants who had achieved BP control were no longer controlled at follow-up (22%, 242/1125 for PLHIV versus 21%, 442/2141 for PNLHIV).

### Regression analysis

**Fig 4:**
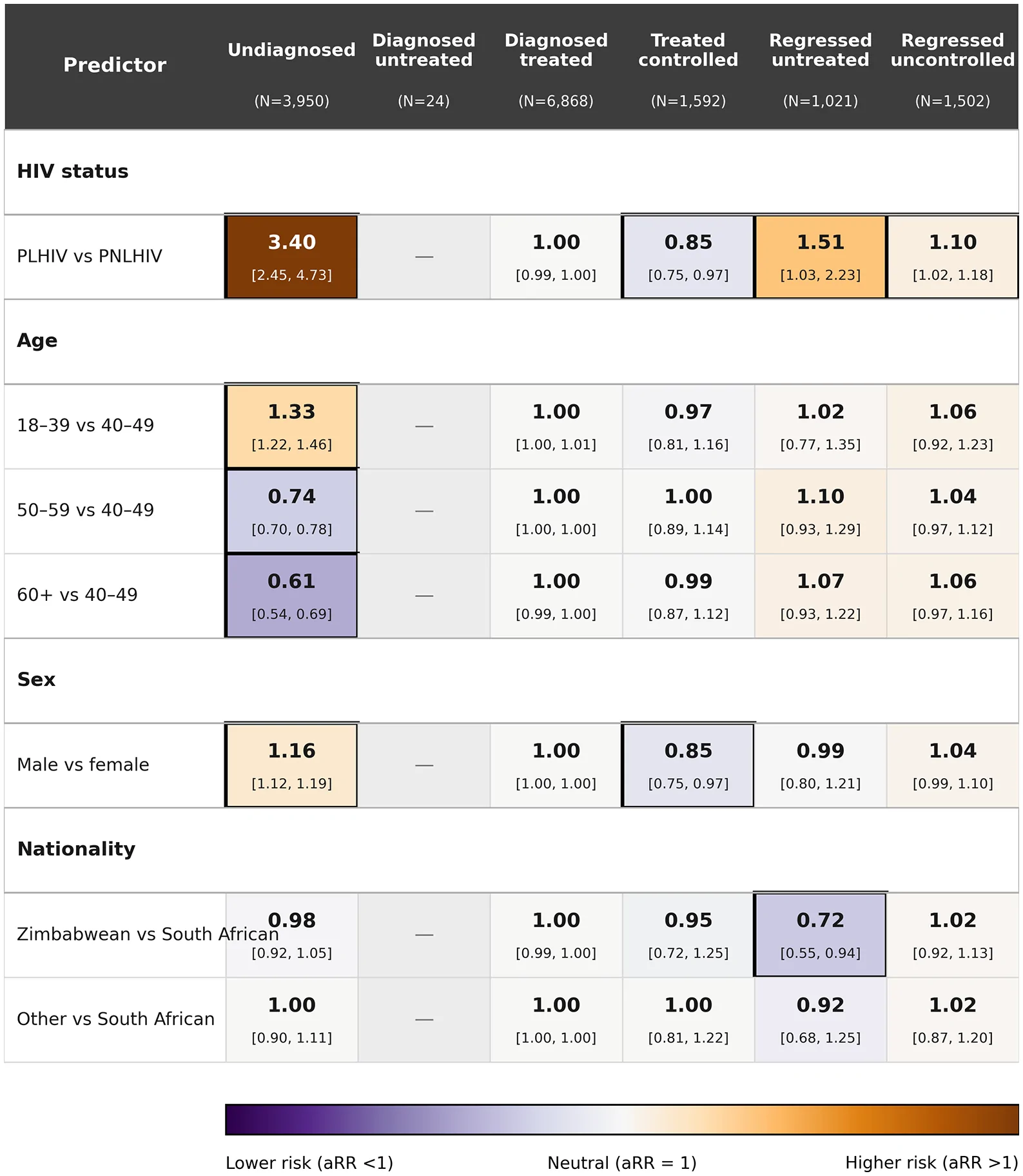
Heatmap of adjusted risk ratios for progression and regression across the longitudinal hypertension care cascade. **Legend:** Cell colour reflects the direction and magnitude of the adjusted risk ratio relative to the reference category, with purple indicating a lower risk and orange indicating a higher risk to the reference category; white/grey cells indicate little or no association. Bold cell borders indicate statistically significant associations, defined as 95% confidence intervals (CIs) that exclude 1.0. **Abbreviations:** aRR, adjusted risk ratio; CI, confidence interval; PLHIV, people living with HIV; PNLHIV, people not living with HIV; Ref, reference category.

The full unadjusted and adjusted regression results are presented in Tables 3 and 4, respectively. Unadjusted and adjusted estimates were broadly consistent across outcomes, although the magnitude of some associations changed after adjustment for age, sex, and nationality. To facilitate interpretation of patterns across outcomes, Fig 4 provides a heatmap visualisation of the adjusted RRs and their statistical significance. Denominators differed slightly across regression models because analyses were restricted to participants eligible for each cascade transition and to observations with complete covariate data.

**Table 3:** Unadjusted risk ratios for progression and regression across the HTN care cascade.

	Remained undiagnosed (*N*=3,950) — unfavourable ↑ *Numerator: n who remained undiagnosed, within that stratum.* *Denominator: N = 3,950, total who remained undiagnosed (all strata).*		Diagnosed, not yet treated (*N*=24) — descriptive only* Numerator: n who were diagnosed but not yet treated, within that stratum. *Denominator: N = 24, total who were diagnosed but not yet treated (all strata).*		Progressed to treatment (*N*=6,868) — favourable ↑ *Numerator: n who progressed to treatment, within that stratum.* *Denominator: N = 6,868, total who progressed to treatment (all strata).*		Achieved BP control (*N*=1,592) — favourable ↑ *Numerator: n who achieved BP control, within that stratum.* *Denominator: N = 1,592, total who achieved BP control (all strata).*		Regressed to untreated (*N*=1,021) — unfavourable ↑ *Numerator: n who regressed to untreated, within that stratum.* *Denominator: N = 1,021, total who regressed to untreated (all strata).*		Regressed to uncontrolled (*N*=1,502) — unfavourable ↑ *Numerator: n who regressed to uncontrolled, within that stratum.* *Denominator: N = 1,502, total who regressed to uncontrolled (all strata).*	
	*n* (%) of outcome group	Unadjusted RR (95% CI)	*n* (%) of outcome group	Unadjusted RR (95% CI)	*n* (%) of outcome group	Unadjusted RR (95% CI)	*n* (%) of outcome group	Unadjusted RR (95% CI)	*n* (%) of outcome group	Unadjusted RR (95% CI)	*n* (%) of outcome group	Unadjusted RR (95% CI)
**HIV status**												
PNLHIV	494/3950 (12.51)	Ref	14/24 (58.33)	-	4050/6868 (58.97)	Ref	1015/1592 (63.76)	Ref	504/1021 (49.36)	Ref	884/1502 (58.85)	Ref
PLHIV	3456/3950 (87.49)	3.93[2.79, 5.53]	10/24 (41.67)	-	2818/6868 (41.03)	1.00[0.99, 1.01]	577/1592 (36.24)	0.85[0.75, 0.96]	517/1021 (50.64)	1.47[0.99, 2.18]	618/1502 (41.15)	1.09[1.01, 1.17]
**Age category (in years)**												
18–39	1072/3811 (28.13)	1.38[1.23, 1.54]	0/24 (0)	-	558/6722 (8.30)	1.00[1.00, 1.01]	116/1560 (7.44)	0.99[0.82, 1.18]	80/1005 (7.96)	0.99[0.74, 1.31]	121/1470 (8.23)	1.05[0.91, 1.22]
40–49	1624/3811 (42.61)	Ref	9/24 (37.50)	-	2145/6722 (31.91)	Ref	488/1560 (31.28)	Ref	310/1005 (30.85)	Ref	420/1470 (28.57)	Ref
50–59	844/3811 (22.15)	0.68[0.63, 0.73]	8/24 (33.33)	-	2385/6722 (35.48)	1.00[1.00, 1.00]	552/1560 (35.38)	1.00[0.89, 1.12]	381/1005 (37.91)	1.11[0.95, 1.29]	553/1470 (37.62)	1.03[0.96, 1.11]
60+	271/3811 (7.11)	0.39[0.31, 0.49]	7/24 (29.17)	-	1634/6722 (24.31)	1.00[0.99, 1.00]	404/1560 (25.90)	1.03[0.92, 1.17]	234/1005 (23.28)	0.99[0.82, 1.19]	376/1470 (25.58)	1.03[0.94, 1.12]
**Sex**												
Female	2533/3950 (64.13)	Ref	17/24 (70.83)	-	4670/6868 (68.00)	Ref	1108/1592 (69.60)	Ref	695/1021 (68.07)	Ref	1056/1502 (70.31)	Ref
Male	1417/3950 (35.87)	1.07[0.99, 1.17]	7/24 (29.17)	-	2198/6868 (32.00)	1.00[1.00, 1.00]	484/1592 (30.40)	0.86[0.77, 0.96]	326/1021 (31.93)	0.99[0.81, 1.21]	446/1502 (29.69)	1.04[0.99, 1.09]
**Nationality**												
South African	2615/3950 (66.20)	Ref	16/24 (66.67)	-	4871/6868 (70.92)	Ref	1168/1592 (73.37)	Ref	770/1021 (75.42)	Ref	1071/1502 (71.30)	Ref
Zimbabwean	994/3950 (25.16)	1.19[1.04, 1.37]	6/24 (25.00)	-	1295/6868 (18.86)	1.00[0.99, 1.00]	272/1592 (17.09)	0.94[0.73, 1.21]	153/1021 (14.99)	0.75[0.59, 0.96]	275/1502 (18.31)	1.02[0.92, 1.12]
Other	341/3950 (8.63)	0.95[0.74, 1.22]	2/24 (8.33)	-	702/6868 (10.22)	1.00[1.00, 1.00]	152/1592 (9.55)	0.99[0.81, 1.22]	98/1021 (9.60)	0.88[0.64, 1.21]	156/1502 (10.39)	1.01[0.86, 1.20]

**Legend:** For each outcome, ***N*** denotes the total number of participants classified with that outcome. The ***n* (%)** values describe the composition of each outcome group across covariate strata and should not be interpreted as stratum-specific risks. Unadjusted risk ratios (RRs) represent the relative likelihood of each outcome for each covariate category compared with the reference category, without adjustment for other covariates. Column headings indicate whether a higher RR reflects a more favourable or less favourable outcome, depending on the outcome being assessed. The diagnosed but not yet treated outcome was not modelled because of the small number of participants in this category (*n* = 24); descriptive counts and percentages are presented instead. Denominators vary slightly across analyses because of missing data for some covariates or outcomes. As a result, the following denominators were used: age and undiagnosed (*n* = 3,811); age and diagnosed and treated (*n* = 6,722); age and treated and controlled (*n* = 1,560); age and regressed to untreated (*n* = 1,005); and age and regressed to being uncontrolled (*n* = 1,470). For all other covariates, the total outcome denominators correspond to those reported for each outcome in the table. Incident and prevalent cases: participants contributing to each cascade stage include both those newly identified during the study observation period (e.g., newly diagnosed with HTN or newly initiated on antihypertensive treatment) and those who entered the study already aware of their diagnosis or already receiving treatment. As this analysis did not distinguish between incident and prevalent cases within each stage, the findings should be interpreted in the context of the overall study population.

Participants contributing to each cascade stage included both those newly identified during the study observation period and those who entered the study already aware of their diagnosis or already receiving treatment. As the analysis did not distinguish between incident and prevalent cases within each stage, findings should be interpreted in the context of the overall study population.

**Abbreviations:** RR, risk ratio; CI, confidence interval; HTN, hypertension; PLHIV, people living with HIV; PNLHIV, people not living with HIV; Ref, reference category.

**Table 4:** Adjusted risk ratios for progression and regression across the HTN care cascade.

	Remained undiagnosed (*N*=3,950) — unfavourable ↑ *Numerator: n who remained undiagnosed, within that stratum.* *Denominator: N = 3,950, total who remained undiagnosed (all strata).*		Diagnosed, not yet treated (*N*=24) — descriptive only* Numerator: n who were diagnosed but not yet treated, within that stratum. *Denominator: N = 24, total who were diagnosed but not yet treated (all strata).*		Progressed to treatment (*N*=6,868) — favourable ↑ *Numerator: n who progressed to treatment, within that stratum.* *Denominator: N = 6,868, total who progressed to treatment (all strata).*		Achieved BP control (*N*=1,592) — favourable ↑ *Numerator: n who achieved BP control, within that stratum.* *Denominator: N = 1,592, total who achieved BP control (all strata).*		Regressed to untreated (*N*=1,021) — unfavourable ↑ *Numerator: n who regressed to untreated, within that stratum.* *Denominator: N = 1,021, total who regressed to untreated (all strata).*		Regressed to uncontrolled (*N*=1,502) — unfavourable ↑ *Numerator: n who regressed to uncontrolled, within that stratum.* *Denominator: N = 1,502, total who regressed to uncontrolled (all strata).*	
	*n* (%) of outcome group	aRR (95% CI)	*n* (%) of outcome group	aRR (95% CI)	*n* (%) of outcome group	aRR (95% CI)	*n* (%) of outcome group	aRR (95% CI)	*n* (%) of outcome group	aRR (95% CI)	*n* (%) of outcome group	aRR (95% CI)
**HIV status**												
PNLHIV	494/3950 (12.51)	Ref	14/24 (58.33)	-	4050/6868 (58.97)	Ref	1015/1592 (63.76)	Ref	504/1021 (49.36)	Ref	884/1502 (58.85)	Ref
PLHIV	3456/3950 (87.49)	3.40[2.45, 4.73]	10/24 (41.67)	-	2818/6868 (41.03)	1.000[0.994, 1.004]	577/1592 (36.24)	0.85[0.75, 0.97]	517/1021 (50.64)	1.51[1.03, 2.23]	618/1502 (41.15)	1.10[1.02, 1.18]
**Age category (in years)**												
18–39	1072/3811 (28.13)	1.33[1.22, 1.46]	0/24 (0)	-	558/6722 (8.30)	1.004[1.000, 1.008]	116/1560 (7.44)	0.97[0.81, 1.16]	80/1005 (7.96)	1.02[0.77, 1.35]	121/1470 (8.23)	1.06[0.92, 1.23]
40–49	1624/3811 (42.61)	Ref	9/24 (37.50)	-	2145/6722 (31.91)	Ref	488/1560 (31.28)	Ref	310/1005 (30.85)	Ref	420/1470 (28.57)	Ref
50–59	844/3811 (22.15)	0.74[0.70, 0.78]	8/24 (33.33)	-	2385/6722 (35.48)	1.001[0.998, 1.004]	552/1560 (35.38)	1.00[0.89, 1.14]	381/1005 (37.91)	1.10[0.93, 1.29]	553/1470 (37.62)	1.04[0.97, 1.12]
60+	271/3811 (7.11)	0.61[0.54, 0.69]	7/24 (29.17)	-	1634/6722 (24.31)	1.000[0.994, 1.005]	404/1560 (25.90)	0.99[0.87, 1.12]	234/1005 (23.28)	1.07[0.93, 1.22]	376/1470 (25.58)	1.06[0.97, 1.16]
**Sex**												
Female	2533/3950 (64.13)	Ref	17/24 (70.83)	-	4670/6868 (68.00)	Ref	1108/1592 (69.60)	Ref	695/1021 (68.07)	Ref	1056/1502 (70.31)	Ref
Male	1417/3950 (35.87)	1.16[1.12, 1.19]	7/24 (29.17)	-	2198/6868 (32.00)	1.001[0.998, 1.003]	484/1592 (30.40)	0.85[0.75, 0.97]	326/1021 (31.93)	0.99[0.80, 1.21]	446/1502 (29.69)	1.04[0.99, 1.10]
**Nationality**												
South African	2615/3950 (66.20)	Ref	16/24 (66.67)	-	4871/6868 (70.92)	Ref	1168/1592 (73.37)	Ref	770/1021 (75.42)	Ref	1071/1502 (71.30)	Ref
Zimbabwean	994/3950 (25.16)	0.98[0.92, 1.05]	6/24 (25.00)	-	1295/6868 (18.86)	0.998[0.992, 1.004]	272/1592 (17.09)	0.95[0.72, 1.25]	153/1021 (14.99)	0.72[0.55, 0.94]	275/1502 (18.31)	1.02[0.92, 1.13]
Other	341/3950 (8.63)	1.00[0.90, 1.11]	2/24 (8.33)	-	702/6868 (10.22)	1.000[0.996, 1.005]	152/1592 (9.55)	1.00[0.81, 1.22]	98/1021 (9.60)	0.92[0.68, 1.25]	156/1502 (10.39)	1.02[0.87, 1.20]

**Legend:** For each outcome, ***N*** denotes the total number of participants classified with that outcome. The ***n*** **(%)** values describe the composition of the outcome group across covariate strata and should not be interpreted as stratum-specific risks. Relative likelihood of each outcome was estimated using aRRs from multivariable Poisson regression models, adjusted for the other covariates included in the model. Column headings indicate whether a higher aRR reflects a more favourable or less favourable outcome, depending on the outcome being assessed. Estimates are shown as aRR estimated from Poisson regression models for each outcome (progression and regression) on the variables of interest (HIV status, age, sex, and nationality). Favourable versus unfavourable: outcomes are labelled “favourable” where a higher aRR reflects a greater likelihood of positive movement through the HTN care cascade (progressing to treatment or achieving BP control), and “unfavourable” where a higher aRR reflects a greater likelihood of negative movement or stagnation in the cascade (remaining undiagnosed or regressing to untreated or uncontrolled status). This direction differs across outcomes and is indicated in each column header. *Due to the small number of individuals classified as diagnosed but untreated (*n* = 24 overall), we did not fit multivariable regression models for this outcome; we report descriptive n/N and crude percentages by covariate strata instead.

Age and outcome variables contained some missing values, which were excluded from these analyses. As a result, the following denominators were used: age and undiagnosed (*n* = 3,811); age and diagnosed and treated (*n* = 6,722); age and treated and controlled (*n* = 1,560); age and regressed to untreated (n = 1,005); and age and regressed to being uncontrolled (*n* = 1,470). For all other covariates, the total outcome denominators correspond to those reported for each outcome in the table. Incident and prevalent cases: participants contributing to each cascade stage include both those newly identified during the study observation period (e.g., newly diagnosed with HTN or newly initiated on antihypertensive treatment) and those who entered the study already aware of their diagnosis or already receiving treatment. As this analysis did not distinguish between incident and prevalent cases within each stage, the findings should be interpreted in the context of the overall study population.

**Abbreviations:** aRR, adjusted risk ratio; CI, confidence interval; HTN, hypertension; PLHIV, people living with HIV; PNLHIV, people not living with HIV; Ref, reference category.

**Variables controlled for:** All adjusted risk ratios (aRR) in this table were estimated using multivariable Poisson regression models adjusting for HIV status, age category (18–39, 40–49, 50–59, 60+), sex, and nationality (South African, Zimbabwean, Other), with clinic-level clustering. Each covariate-specific aRR reflects the association between that covariate and the outcome after adjustment for the other three covariates in the same model. The same four covariates were included across all five modelled outcomes. The diagnosed, not yet treated outcome was not modelled because of the small number of participants (*n* = 24).

### Diagnosis of HTN

PLHIV were more likely to remain undiagnosed compared with PNLHIV (aRR 3.40; 95% [CI 2.45,4.73]; *p* < 0.001). Individuals aged 18–39 years had a higher risk of being undiagnosed with HTN (aRR 1.33; 95% [CI 1.22,1.46]; *p* < 0.001), while older adults were less likely to remain undiagnosed (50–59 years: aRR 0.74; 95% [CI 0.70,0.78]; *p* < 0.001; ≥ 60 years: aRR 0.61; 95% [CI 0.54,0.69]; *p* < 0.001). Male sex was also associated with undiagnosed HTN (aRR 1.16; 95% [CI 1.12,1.19]; *p* < 0.001).

### HTN control among treated individuals

Among those receiving antihypertensive treatment, PLHIV were less likely to achieve BP control (aRR 0.85; 95% [CI 0.75,0.97]; *p* = 0.012).

### Regression from a more advanced to a prior stage of the HTN care cascade

PLHIV had a higher risk of regressing to untreated HTN (aRR 1.51; 95% [CI 1.03,2.23]; *p* = 0.037) and of regressing from controlled to uncontrolled HTN (aRR 1.10; 95% [CI 1.02,1.18]; *p* = 0.010). Zimbabwean nationality was associated with a lower risk of regression to untreated HTN compared with South African nationality (aRR 0.72; 95% [CI 0.55,0.94]; *p* = 0.016).

## Discussion

This study provides longitudinal evidence on progression and regression across the HTN care cascade among adults with and without HIV attending public primary healthcare clinics in Johannesburg, South Africa. The findings reveal persistent gaps and distinct differences between PLHIV and PNLHIV. HTN prevalence was markedly higher among PNLHIV than among PLHIV, yet rates of HTN diagnosis were substantially lower among PLHIV despite their frequent contact with healthcare services. Although treatment initiation rates were comparable across groups, sustained BP control remained low overall. Follow-up duration was generally comparable between PLHIV and PNLHIV, indicating that the observed differences in HTN care cascade outcomes are unlikely to be attributable to differential observation time. A higher proportion of PLHIV regressed to being untreated or lost BP control over time, underscoring significant challenges in maintaining continuity of HTN care within HIV-focussed health programmes. These findings should be interpreted in the context of a clinic-attending population and may not be generalisable to individuals not engaged in routine healthcare

The disproportionately higher prevalence of HTN among PNLHIV (53.5%) compared with PLHIV (29.3%) is likely attributable to both demographic and structural factors. PNLHIV in this cohort were considerably older, and older age is a well-established to be associated with high BP [17]. However, the predominance of PLHIV may reflect the service profile of region F primary healthcare clinics, where the majority of chronic care visits are for HIV-related services rather than other chronic conditions. As parent iHEART-SA trial eligibility required participants to be receiving chronic care and to have an existing medical record at the clinic, individuals accessing HIV care were more likely to meet the inclusion criteria and be enrolled. Consequently, PNLHIV in this cohort may represent a selected subgroup already engaged in healthcare services for chronic or other medical conditions, rather than the broader population of PNLHIV in the community. This may partially explain the higher observed prevalence of HTN and cardiometabolic risk factors within this group, while PLHIV who attend ART clinics might not receive routine HTN screening. This aligns with prior South African evidence demonstrating inconsistent HTN screening among PLHIV despite regular clinic attendance [18].

Our longitudinal results correspond with findings from Mauer and colleagues [12], who demonstrated substantial treatment discontinuation and loss of BP control across several MICs, underscoring the global challenge of maintaining longitudinal HTN control. In this study, nearly one-third of PLHIV who achieved BP control reverted to being untreated, compared with one-fifth among PNLHIV. This pattern suggests that the most vulnerable point in the HTN care cascade for PLHIV is treatment continuity rather than initiation. Factors contributing to this may include competing clinical priorities within HIV care, therapeutic inertia among healthcare providers, and patient-level barriers such as pill burden and prioritisation of ART adherence [19–23].

Conversely, PNLHIV, who typically access care primarily for HTN, may benefit from more focussed and consistent follow-up, leading to better continuity [19,20]. These observations reflect the consequences of health system organisation: vertically oriented HIV programmes achieve exceptional ART continuity but may inadequately support comorbid NCD management. Comparable challenges have been reported in other sub-Saharan African settings, where fragmented care structures have constrained the delivery of integrated chronic disease services [21,22].

Several factors may potentially contribute to the poorer longitudinal HTN care cascade outcomes observed among PLHIV. Although differentiated and vertically structured HIV programs may inadvertently limit integration of HTN management within routine HIV care [21,22], additional mechanisms are also plausible. PLHIV may experience higher medication burden due to lifelong ART use, which could affect adherence to antihypertensive therapy or lead to prioritisation of HIV treatment over HTN management [24,25]. Potential pharmacologic interactions between ART regimens and antihypertensive medications may also complicate BP control [25]. In addition, BP monitoring and HTN management may not always be prioritised during routine HIV-focussed clinical encounters, particularly in resource-constrained settings where consultation time and staffing are limited [26]. These factors may likely interact in complex ways and could collectively contribute to observed differences in progression and regression across the HTN care cascade.

In adjusted analyses, HIV status was the factor most consistently associated with engagement across the longitudinal HTN care cascade, with PLHIV substantially more likely to remain undiagnosed, less likely to achieve BP control once treated, and more likely to regress to untreated or uncontrolled HTN. These findings suggest that frequent contact with HIV services does not translate into effective HTN detection or sustained management, underscoring persistent gaps in HIV/NCD integration and the need for routine BP screening and longitudinal HTN care within HIV programmes [27–29]. Although several associations reached statistical significance, some effect sizes were modest and should therefore be interpreted cautiously with respect to clinical relevance.

Younger adults and men were more likely to remain undiagnosed, reflecting patterns reported in population-based studies of HTN awareness and diagnosis, and highlighting the limitations of facility-based screening for these groups [30–34]. Although younger adults showed a marginally higher likelihood of being treated, the magnitude of this association was minimal.

Regression after treatment initiation was common, particularly among PLHIV, consistent with prior evidence showing that treatment initiation alone does not ensure sustained BP control in routine care settings, especially in the absence of integrated services and long-term adherence support [20, 33, 35,36]. These findings should be interpreted in light of how treatment was measured, as treatment status was based on recorded medication use at clinic visits rather than continuous prescription data.

The lower risk of regression to untreated HTN observed among Zimbabwean participants may reflect greater continuity of care and sustained engagement once treatment is initiated, patterns that have been observed in other studies of chronic disease care among migrant populations in Southern Africa [37,38]. Similar findings in NCD cascade analyses suggest that, despite barriers to initial access, nonnational populations may experience comparable or improved retention once engaged in care; however, the mechanisms underlying these differences warrant further investigation.

These findings should also be interpreted within the broader context of migration and healthcare access in South Africa. Migrant populations may experience complex and sometimes competing influences on healthcare engagement, including barriers related to mobility, continuity of care, documentation challenges, and experiences of social exclusion or discrimination within healthcare settings. At the same time, migrant individuals successfully linked to chronic care services may represent a relatively stable subgroup with sustained healthcare utilisation once engaged in care. Differences in healthcare-seeking behaviours and patterns of clinic engagement may therefore contribute to the observed association. However, our study was not specifically designed to evaluate migrant health outcomes or the broader structural determinants shaping healthcare access among migrant populations.

These findings have important implications for chronic disease care in high HIV-burden settings. PLHIV in this study experienced lower HTN diagnosis, poorer BP control, and higher regression across the HTN care cascade, suggesting persistent gaps in longitudinal HTN management within routine HIV care services. Prior studies suggest that integrating NCD services into HIV care may improve screening, follow-up, and continuity of chronic disease management [18,39–41]. In addition, emerging evidence from community- and home-based HTN care models in South Africa demonstrates the potential of differentiated and patient-centred approaches to improve BP control outside traditional facility-based care platforms [42]. However, the present study was not designed to directly compare service-delivery models, and further research is needed to determine which strategies are most effective for sustaining longitudinal HTN control in routine care settings [18,39–42].

Our study had several limitations. Data were collected from routine medical records, which may be subject to poor recording, and incomplete documentation. For example, BP measurements, HTN diagnoses and treatment initiation may not always be recorded accurately or completely, and/or discontinuation of treatment could be under- or over-reported. Treatment status was based on medications documented in routine medical records at each visit, and information on prescription duration or adherence was not available. As a result, treatment continuity between visits could not be directly assessed and may have been misclassified in some instances.

An additional methodological limitation relates to the relatively small number of clusters (nine clinics) used to estimate cluster-robust standard errors in the regression models. Cluster-robust variance estimation generally performs best with a larger number of clusters (commonly 30–40 or more); with fewer clusters, CIs may be modestly anticonservative, and borderline findings should therefore be interpreted with caution. However, the principal findings were consistent across analyses and supported by relatively precise effect estimates for the primary outcomes, suggesting that this limitation is unlikely to materially affect the overall interpretation of the results. Future studies could consider statistical methods specifically developed for analyses with few clusters, such as the wild cluster bootstrap, to further evaluate the robustness of these findings

Our study did not account for several potentially important factors associated with HTN care, including medication adherence, patient socioeconomic status, provider-level factors such as training or attitudes toward integrated care, and clinical covariates such as BMI, which were not collected as part of the parent study. The absence of this data limits the ability to fully describe observed differences in progression and regression between PLHIV and PNLHIV. Nonetheless, some of these factors have been explored and reported in a qualitative study conducted in this setting [40].

Another limitation of this analysis is the variation in follow-up duration among participants. Enrolment spanned from August 2022 to May 2024, and follow-up extending through May 2025, resulting in heterogeneous observation periods across the cohort. Participants with longer follow-up had greater opportunity to experience both progression and regression across stages of the HTN care cascade, which may have influenced the observed frequency of transitions. Consequently, progression and regression outcomes should be interpreted as reflecting observed longitudinal movement during available follow-up rather than standardised transition rates over fixed time intervals. Although follow-up duration was generally comparable between PLHIV and PNLHIV, differences in observation time may still have contributed to variation in cascade outcomes. In addition, ascertainment of progression and regression depended on the frequency of documented clinical encounters at which BP and treatment status were recorded, rather than follow-up duration alone. PNLHIV had a higher median number of documented follow-up visits than PLHIV (median 3 [IQR 1–5] versus 2 [IQR 1–4]; Table 2), providing greater opportunity for transitions across the HTN care cascade to be observed. Consequently, differences in visit frequency should be considered when interpreting comparisons in progression and regression between the two groups.

In addition, this secondary analysis was conducted within the broader iHEART-SA stepped-wedge implementation trial, during which clinics transitioned from usual care to an integrated HTN/HIV intervention at different timepoints. As the present analysis did not explicitly model intervention exposure or study period, it is possible that implementation activities influenced observed progression and regression across the HTN care cascade. Consequently, observed longitudinal transitions may partly reflect the effects of ongoing intervention rollout and changing clinic practices over time.

Our analysis was also limited by the challenge of capturing movement across the HTN care cascade. Participants could experience both progression and regression during follow-up, and the timing between observations varied. As a result, transitions between cascade stages may not fully reflect the true continuity or duration of engagement in care. Consequently, our estimates of movement along the cascade should be interpreted as indicative of stage changes between recorded visits rather than continuous trajectories over time.

The study sample was drawn from a single urban setting in South Africa and was restricted to individuals attending primary healthcare clinics; therefore, the findings may not be generalisable to other settings or to the broader population, particularly individuals not engaged in routine care. Given that study participation and follow-up depended on engagement with the healthcare system, individuals who are less likely to seek care or who disengage from care may be underrepresented. This could lead to an overestimation of progression rates and an underestimation of regression.

In addition, measures of ART adherence and retention in HIV care were not consistently available within the routine medical records used for this secondary analysis and therefore could not be included as covariates in the regression models. These factors may represent important markers of healthcare engagement among PLHIV and could influence opportunities for HTN screening, treatment, follow-up, and BP control. Consequently, residual confounding related to differential healthcare engagement may have influenced observed associations between HIV status and HTN care cascade outcomes.

The overrepresentation of PLHIV reflects the underlying service utilisation profile of region F primary healthcare clinics, where the majority of chronic care visits are for HIV-related services rather than other chronic conditions. This sampling bias, arising from clinic-based recruitment within chronic care services, should be considered when interpreting comparative findings between PLHIV and PNLHIV.

As with many studies using routine data, there is the possibility of missing data or measurement errors in BP readings, diagnosis, diagnosis dates, or treatment status. This might contribute to bias in our results. While this study offers valuable longitudinal insights into the longitudinal HTN care cascade for PLHIV and PNLHIV, its findings should be interpreted in light of these methodological and contextual limitations.

This study identifies substantial gaps across the longitudinal HTN care cascade among adults attending urban primary healthcare clinics in South Africa, with pronounced disparities among PLHIV. Less than half of PLHIV with high BP were diagnosed with HTN, and only 45% of those treated achieved BP control. High levels of regression, including treatment discontinuation and loss of control, were observed across both PLHIV and PNLHIV. These findings underscore persistent gaps in longitudinal HTN care and highlight the potential importance of integrated approaches that strengthen HTN screening, diagnosis, and sustained BP control within routine HIV care services. Future studies integrating more detailed patient- and provider-level data and applying alternative longitudinal state-transition approaches, such as Markov chain modelling, may provide additional insight into movement across HTN care cascade stages over time.

### Ethical approval

Written informed consent was obtained from all participants prior to enrolment in the iHEART-SA trial. Ethical approval for the parent trial was obtained from the Human Research Ethics Committee (Medical) of the University of the Witwatersrand (M211160) and the Johannesburg District Research Office (NHRD No. GP_202111_015). Ethical approval for this secondary analysis was also obtained from the Human Research Ethics Committee (Medical) of the University of the Witwatersrand (M2411128).

## Supporting information

S1 Fig Flow diagram illustrating participants with known HIV status who were included in the study from August 2022 to May 2024. Legend: Flow diagram showing participant screening, eligibility assessment, consent, and chart review completion for inclusion in the study. Of 37,620 individuals screened in the iHEART-SA study, 23,855 participants with known HIV status and completed chart review records were included in the analysis. Reasons for exclusion at each stage are shown.
(TIFF)

S1 Appendix REDCap data dictionary (codebook) for the medical record abstraction component of the iHEART-SA study. Legend: This appendix contains the REDCap dictionary codebook for medical record instrument (data abstraction from routine medical records). The codebook includes the predefined medication fields used to classify antihypertensive treatment, as well as variables used to determine hypertension diagnosis, treatment status, blood pressure measurements (for blood pressure control), and longitudinal transitions across the hypertension care cascade. The appendix is provided to enhance transparency and reproducibility of the study methods.
(XLSX)

S1 Table List of hypertensive medications captured in the iHEART-SA medical record abstraction instrument. Legend: This table lists the five pre-specified antihypertensive medications available in the medical record abstraction instrument (hydrochlorothiazide, enalapril, amlodipine, furosemide, and atenolol), their ATC codes, drug classes, and recorded dose bands. Medications outside this list were captured via free-text fields and verified by clinical staff against the patient’s documented diagnosis/indication before classification as antihypertensive treatment (S1 Appendix).
(PDF)

S1 Protocol Legend: Study protocol for “Progression and regression in the hypertension (HTN) care continuum in people living with HIV (PLHIV) and those without HIV (PNLHIV): a longitudinal study,” a sub-study of the iHEART-SA project (Protocol Version 1.0, October 2024). Legends: This protocol describes the study design, objectives, study population, sample size calculation, data sources, and statistical analysis plan for the secondary data analysis presented in this manuscript.
(PDF)

S1 Data Stata do-file used for data cleaning, variable derivation, and statistical analysis. Legend: This file contains the Stata code used to derive the hypertension care cascade variables (diagnosis, treatment, and control status), classify participants by HIV status and sociodemographic characteristics, and estimate the adjusted risk ratios reported in Tables 3 and 4, provided to support transparency and reproducibility of the analytic approach.
(PDF)

S1 Checklist Legend: Completed Strengthening the Reporting of Observational Studies in Epidemiology (STROBE) checklist for this longitudinal cohort study, indicating the location within the manuscript where each recommended reporting item is addressed. The checklist is reproduced from the STROBE Statement, licensed under the Creative Commons Attribution 4.0 International License (CC BY 4.0). Information on the STROBE Initiative is available at https://www.strobe-statement.org/. von Elm E, Altman DG, Egger M, Pocock SJ, Gøtzsche PC, Vandenbroucke JP; STROBE Initiative. The Strengthening the Reporting of Observational Studies in Epidemiology (STROBE) Statement: Guidelines for Reporting Observational Studies. *PLoS Med*. 2007;4(10):e296. https://doi.org/10.1371/journal.pmed.0040296.
(PDF)

S2 Data Legend: This dataset contains the minimal de-identified participant-level data and variables required to reproduce the analyses and findings reported in the manuscript, including variables used to define HIV status, hypertension care cascade stages, blood pressure control, antihypertensive treatment, participant characteristics, follow-up, and study outcomes. Direct personal identifiers have been removed.
(CSV)

## Abbreviations

AI: artificial intelligence
aRRs: Adjusted risk ratios
BP: blood pressure
CIs: confidence intervals
DBP: diastolic BP
HTN: Hypertension
iHEART-SA: Heart Health in South Africa
IQR: interquartile ranges
MICs: middle-income countries
NCDs: noncommunicable diseases
PLHIV: people living with HIV
PNLHIV: people not living with HIV
RRs: risk ratios
SBP: systolic BP
SD: standard deviation
STROBE: Strengthening the Reporting of Observational Studies in Epidemiology
