# Supplementary material for: The longitudinal care cascade for hypertension in people with and without HIV in South Africa: A clinic-based observational study
Source: PLoS Med. 2026 Sep 11;23(9):e1004957. doi: 10.1371/journal.pmed.1004957 (PMC13567817; doi:10.1371/journal.pmed.1004957)
Supplement: Supplementary file 1 [file pmed.1004957.s003.pdf]

S2 Table.

List of hypertensive medications captured in the iHEART-SA medical record abstraction instrument.

This table lists the five pre-specified antihypertensive medications available in the medical record abstraction instrument (hydrochlorothiazide, enalapril, amlodipine, furosemide, and atenolol), their ATC codes, drug classes, and recorded dose bands. Medications outside this list were captured via free-text fields and verified by clinical staff against the patient's documented diagnosis/indication before classification as antihypertensive treatment (S1 Appendix).

Medication (generic name)	ATC code	Drug class	Notes
Hydrochlorothiazide	C03AA03	Thiazide diuretic	*Also referred to as HCTZ in the CRF.*
Enalapril	C09AA02	ACE inhibitor	*Recorded in the CRF field label as "Enalipril" (data-entry spelling); the intended medication is enalapril.*
Amlodipine	C08CA01	Calcium channel blocker (dihydropyridine)	
Furosemide	C03CA01	Loop diuretic	
Atenolol	C07AB03	Beta-blocker	
Other / unlisted antihypertensive	—	Not restricted to a specific class	*Free-text entries were reviewed in real time during data abstraction by a clinician or nurse on the study team, who cross-checked each entry against the patient's documented diagnosis/indication in the medical record to confirm hypertension-related use before classification as antihypertensive treatment.*

Prescribed dose bands captured in the CRF	
5 mg, twice daily	10 mg, twice daily
12.5 mg, once daily	20 mg, once daily
25 mg, once daily	40 mg, once daily
50 mg, once daily	Other (free text)

Note: Dose bands were recorded generically for whichever medication was selected (i.e., the same eight dose options applied to any of the five named medications, or to an "Other" entry), rather than being medication-specific fields. ATC codes are from the WHO Collaborating Centre for Drug Statistics Methodology (ATC/DDD Index). Source: S1 Appendix (REDCap data dictionary), fields previous_htn_regimen1–8 / file_review_htn_regimen1–8 and prev_prescribed_htn_dos_1–8 / file_review_htn_dosage1–8.
