# Supplementary material for: The longitudinal care cascade for hypertension in people with and without HIV in South Africa: A clinic-based observational study
Source: PLoS Med. 2026 Sep 11;23(9):e1004957. doi: 10.1371/journal.pmed.1004957 (PMC13567817; doi:10.1371/journal.pmed.1004957)
Supplement: Supplementary file 2 [file pmed.1004957.s004.pdf]

**Study title**	**Progression and regression in the hypertension (HTN) care continuum in people living with HIV (PLHIV) and those without HIV (PNLHIV): a longitudinal study**
**Principal investigator (PI)**	Dr. Siphamandla Gumede, Ezintsha, a division of the Wits Health Consortium, University of the Witwatersrand, SA
**Co-Is**	Dr. Samanta Lalla-Edward, Ezintsha, a division of the Wits Health Consortium, University of the Witwatersrand, Johannesburg, South Africa.<br><br>Prof. Jennifer Manne-Goehler, 1) Division of Infectious Diseases, Brigham and Women's Hospital, Harvard Medical School, Boston, MA 02115, USA. 2) MRC/Wits Rural Public Health and Health Transitions Research Unit, School of Public Health, University of Witwatersrand, 2193 Johannesburg, South Africa.<br><br>Dr Elizabeth Kelechi Oladimeji, Ezintsha, a division of the Wits Health Consortium,<br>University of the Witwatersrand, South Africa
**Scientific advisors**	None
**Sponsors**	HLB-SIMPLe Alliance Small Research Project grant
**Protocol Version number**	1.0
**Version date**	October 2024
**HREC protocol number**	
**HREC approval date**	

Confidentiality Statement

This document contains information that is confidential and proprietary. The contents of this document may be disclosed only to study personnel, the Independent Ethics Committee, or duly authorized representatives of regulatory agencies for this purpose under the condition that they maintain confidentiality.

Protocol Title: Progression and regression in the hypertension (HTN) care continuum in people living with HIV (PLHIV) and those without HIV (PNLHIV): a longitudinal study

Protocol Number:

Protocol Version (Date):

This version of the protocol has been read, understood, and approved by:

	21/10/2024
Siphamandla Gumede, MSc, PhD	Date
	21/10/2024
Samanta Lalla-Edward, MA, PhD	Date
	21/10/2024
Jen Manne-Goehler, MD. DSc	Date
KEO	21/10/2024
Elizabeth Kelechi Oladimeji, MPH. PhD	Date

TABLE OF CONTENTS

PROTOCOL SYNOPSIS 4
LIST OF ABBREVIATIONS AND DEFINITIONS OF TERMS 6
BACKGROUND AND SIGNIFICANCE 7
BACKGROUND 7
SIGNIFICANCE 7
STUDY AIMS 8
INNOVATION 8
METHODOLOGY 9
STUDY DESIGN 9
IHEART-SA PRIMARY OUTCOME 10
STUDY PERIOD 11
STUDY POPULATION AND LOCATION 11
SAMPLE SIZE 12
DATA, INCLUSION, AND EXCLUSION CRITERIA 13
OUTCOME MEASUREMENTS 15
STATISTICAL ANALYSES 15
ETHICAL CONSIDERATIONS 16
MENTOR OVERSIGHT 16
DATA INTEGRITY 16
GENERAL DATA PROTECTION 17
DISSEMINATION 17
TIMELINES 18
FUNDING 18
REGULATION STATEMENT 18
STUDY TEAM 18
REFERENCE 19

PROTOCOL SYNOPSIS

**Study design**	This will be a longitudinal study conducted as a sub-study of **I**ntegrating **H**IV and h**EART** health in **S**outh **A**frica (iHEART-SA) utilizing **secondary data analysis**.<br>The iHEART-SA project is a National Institutes of Health-funded project that seeks to evaluate an intervention designed to improve the diagnosis and management of HTN in PLHIV, as well as enhance the delivery of recommended care in selected primary healthcare clinics in Johannesburg, South Africa (M211160).
**Study objectives**	1. Describe longitudinal patterns of progression and regression in the HTN care continuum among PLHIV and PNLHIV in urban South Africa.<br>2. Evaluate the socio-demographic factors associated with progression and regression in the HTN care continuum between PLHIV and PNLHIV in urban South Africa
**Study site**	9 healthcare facilities in Johannesburg Region F (iHEART-SA study sites)
**Duration of participation**	August 2022-September 2024
**Study duration**	12 months
**Primary outcome measures/endpoints**	Estimates will be provided for each outcome (progression and regression across the continuum of care-HTN diagnosis, ant-HTN treatment, and achieving BP control)<br>Estimates will be provided for each outcome (progression and regression) based on the factors of interest (age, gender, and nationality) as well as the exposure variables.<br>"Progression" is the movement of individuals from their baseline stage into a more advanced stage of the hypertension care continuum. Once BP control is achieved, no further progression along the continuum is possible.<br>"Regression" is the backward movement of individuals from their baseline stage into a prior stage of the hypertension care continuum. Once study subjects have been diagnosed, they can no longer be undiagnosed.
**Secondary outcome measures/endpoints**	The difference in mean systolic BP between the intervention and control conditions<br>HTN is classified as mean systolic BP (SBP) ≥ 140 mmHg, mean diastolic BP (DBP) ≥ 90 mmHg, or a reported prior diagnosis of HTN.
**Inclusion criteria**	Patients: The iHEART-SA dataset will be utilized for data extraction (for both PLHIV and PNLHIV).<br>1. Adults ≥18 years living with HIV and hypertension<br>2. Enrolled in the study clinic<br>3. Willing and able to provide informed consent
**Exclusion criteria**	Patients:<br>1. Unable or unwilling to provide informed consent<br>2. Planning to relocate or change service providers/clinics
**Sample size**	We will include all the eligible data in the dataset. However, the minimal sample size is 519 participants (PLHIV=346 and PNLHIV=173).

**Analytic methods**	We will describe and compare individuals' transition through the continuum during the study period by reporting the progression and/or regression of HTN care across all important clinical transitions: undiagnosed, becoming diagnosed (among all those who are undiagnosed), becoming treated (among all those who are untreated), becoming treated and controlled (among all those with uncontrolled BP), stopping treatment (among all those who report taking treatment during the study period), and losing control (among all those with treated and controlled BP). We will conduct compartmental modeling across various care continuum stages for both groups over time. We will report the frequencies, proportions, and probabilities of individuals in the data that made each of the transitions.<br><br>We will also investigate whether certain socio-demographic subgroups are at an increased risk of not moving forward through the continuum or moving backward to prior stages. We will compare and report the differences based on age group, sex, nationality, facility size classification (high, medium, low), and substance use. To estimate the associations, we will use the univariate analysis, chi-squared, Fisher's Exact and student t-tests. To calculate the risk magnitude of differences and adjust for different exposures or distributions across the continuum stages, we will use regression models (Poisson and/or logistic) for progression and regression through the continuum with each sociodemographic characteristic as the main exposure.

LIST OF ABBREVIATIONS AND DEFINITIONS OF TERMS

Abbreviation	Term
ART	Antiretroviral therapy
BP	Blood pressure
CAB	Community advisory board
CPHR	Community participatory health research
HLB-SIMPLe	Heart, Lung, and Blood Co-morbiditieS Implementation Models in People Living with HIV
HTN	Hypertension
iHEART-SA	**I**ntegrating **H**IV and h**EART** health in **S**outh **A**frica
NHLBI	National Heart, Lung, and Blood Institute
PLHIV	People living with HIV
PNLHIV	People not living with HIV
SOPs	Standard Operating Procedures
VL	Viral load

TITLE: Progression and regression in the hypertension (HTN) care continuum in people living with HIV (PLHIV) and those without HIV (PNLHIV): a longitudinal study

BACKGROUND AND SIGNIFICANCE

BACKGROUND

HTN is a well-established risk factor for cardiovascular disease (CVD) in PNLHIV. For many years, HTN has long been reported as a risk factor for CVDs and mortality for PNLHIV in many middle-income countries such as South Africa [1–6]. The prevalence of HTN in PNLHIV in South Africa has doubled since 1998, from 20% to 46% [3–6]. Aging, weight gain family history of HTN, and lifestyle or behavioural factors, such as substance use, are potential causes of the HTN burden among PNLHIV [7,8]. Recent studies have also reported similar factors for PLHIV [8].

HTN has been a growing health concern in PLHIV in recent years. Several recent observational studies have revealed that PLHIV who are on antiretroviral therapy (ART) are more likely to develop HTN than PNLHIV. According to studies conducted in Kenya and South Africa, the prevalence of HTN among PLHIV receiving ART is estimated to vary between 6 and 50% [9–11] and, particularly in South Africa, this is coupled with poor rates of diagnosis, treatment, and sustained control [12]. Numerous epidemiological and demographic factors contribute to the increased prevalence of HTN among PLHIV. The increasing HTN prevalence in PLHIV receiving ART is associated with a heightened risk of developing classic HTN risk factors, such as aging, lifestyle-related risk factors, and comorbid medical conditions [13–15]. Middle-income countries such as South Africa, which bear high burdens of HTN independent of HIV status, are the most affected [16,17].

The disparities in the HTN care continuum are visible and are much worse in the failure to achieve sustained control [18]**.** It is important to note that, on average and regardless of the HIV status, just over 50% of people diagnosed with HTN in South Africa have successfully reached the recommended BP targets of <140mmHg systolic and <90% diastolic [18,19]. A cross-sectional study was carried out in 44 low and middle-income countries (LMICs), involving a total of 1.1 million individuals [18]. It found a prevalence of 17.5% for HTN. Among those affected, only 30% received treatment, and a mere 10% were able to achieve control of their HTN. Notably, countries in sub-Saharan Africa fared significantly worse in comparison to other regions, irrespective of income classification [18]. Overall, HTN identification, management, and sustained control remain a health concern in PLHIV and PNLHIV globally, independent of income status.

SIGNIFICANCE

HTN is a key risk factor for several of the most prevalent causes of death in LMICs, and that the condition may be well controlled at a reasonable cost, tracking the progression and regression through the HTN care continuum is likely to be valuable. Previous research [6] has shown significant losses in the HTN care continuum, highlighting the importance of investigating progression and regression through this care continuum. When developing targeted, evidence-based interventions to manage HTN in LMICs with varying socio-demographics and HIV burdens, it is essential first to identify the areas where HTN-related healthcare is lacking.

Multiple studies have used the HTN care continuum, which encompasses the sequential steps

required in managing HTN, such as diagnosis, treatment, and the attainment of controlled BP [18,20,21]. A drawback of these studies is that they were not designed to adequately capture crucial aspects of the HTN care continuum such as how individuals arrived at a certain continuum stage or whether they progressed or regressed from that stage over time. Hence, there is a scarcity of longitudinal studies that provide descriptions and comparisons of the progression and regression of PLHIV and PNLHIV along the HTN continuum over a period of time in LMICs. Therefore, this study aims to provide a comprehensive description and comparison of the movement across different stages of the HTN care continuum among PLHIV and PNLHIV in Johannesburg region F, South Africa.

STUDY AIMS

We intend to conduct a longitudinal assessment of the hypertension (HTN) care continuum among people living with HIV (PLHIV) and those without HIV (PNLHIV) in a South African metropolitan population with reported adult HTN prevalence rates ranging from 19 to 35% (unpublished iHEART-SA data, Table 2). For this study population, we will describe how patients progress through each stage of the HTN care continuum over nine months. By comparing both PLHIV and PNLHIV, we will look at the progression (from less advanced to advanced) through the HTN care continuum, and the likelihood of regressing from more to less advanced continuum phases. We will also assess whether and how the transitions vary between both groups by age, sex, facility size classification (high, medium, or low volume), and substance use.

Specific aim 1: To describe longitudinal patterns of progression and regression in the HTN care continuum among PLHIV and PNLHIV in urban South Africa. *We will describe and compare how PLHIV and PNLHIV move between each of the stages of the HTN care continuum. We will examine both progression through the care continuum and regression from more to less advanced continuum stages.* **Hypothesis:** There are no significant differences in the rates of progression and regression in the HTN care continuum between PLHIV and PNLHIV. The proportion of individuals with controlled blood pressure (BP) who will regress over the study period will be similar between PLHIV and PNLHIV.

Specific aim 2: To evaluate the socio-demographic factors associated with progression and regression in the HTN care continuum between PLHIV and PNLHIV in urban South Africa. *We will investigate how transitions vary between PLHIV and PNLHIV across key sociodemographics.*
Hypothesis: There are no differences in the socio-demographic factors associated with progression and regression in the HTN care continuum between PLHIV and PNLHIV.

INNOVATION

1. This study utilizes a relatively large dataset for this region, comprising data collected from standard-of-care services within the primary healthcare context. This approach underscores the importance of data collection in public health facilities and its application in research, and implementation science, for enhanced clinical care.
2. By employing advanced statistical analysis techniques to examine sociodemographic data and health between PLHIV and PNLHIV, this analysis will enhance our understanding and management of HTN control outcomes, helping to prevent HTN regression in this specific population demographic.

METHODOLOGY

STUDY DESIGN

This will be a longitudinal study conducted as a sub-study of **I**ntegrating **H**IV and h**EART** health in **S**outh **A**frica (iHEART-SA) utilizing **secondary data analysis**. The iHEART-SA project (M211160) is a National Institutes of Health-funded project that seeks to evaluate an intervention designed to improve the diagnosis and management of HTN in PLHIV, as well as enhance the delivery of recommended care in selected primary healthcare clinics in Johannesburg, South Africa. The iHEART-SA study is conducting an implementation-effectiveness evaluation to address HTN services for PLHIV. It implemented a tailored care model in healthcare facilities (primary care settings) and is assessing effectiveness endpoints (patient-level) and implementation (provider/clinic) simultaneously at nine clinics using a 24-month cluster stepped-wedge effectiveness-implementation type 2 hybrid design.

As part of the iHEART-SA project's implementation population, PNLHIV, much like PLHIV, were recruited and consented to medical chart review. The effectiveness population of interest of the iHEART-SA main study exclusively comprises PLHIV and does not include PLNHIV (Figure 1).

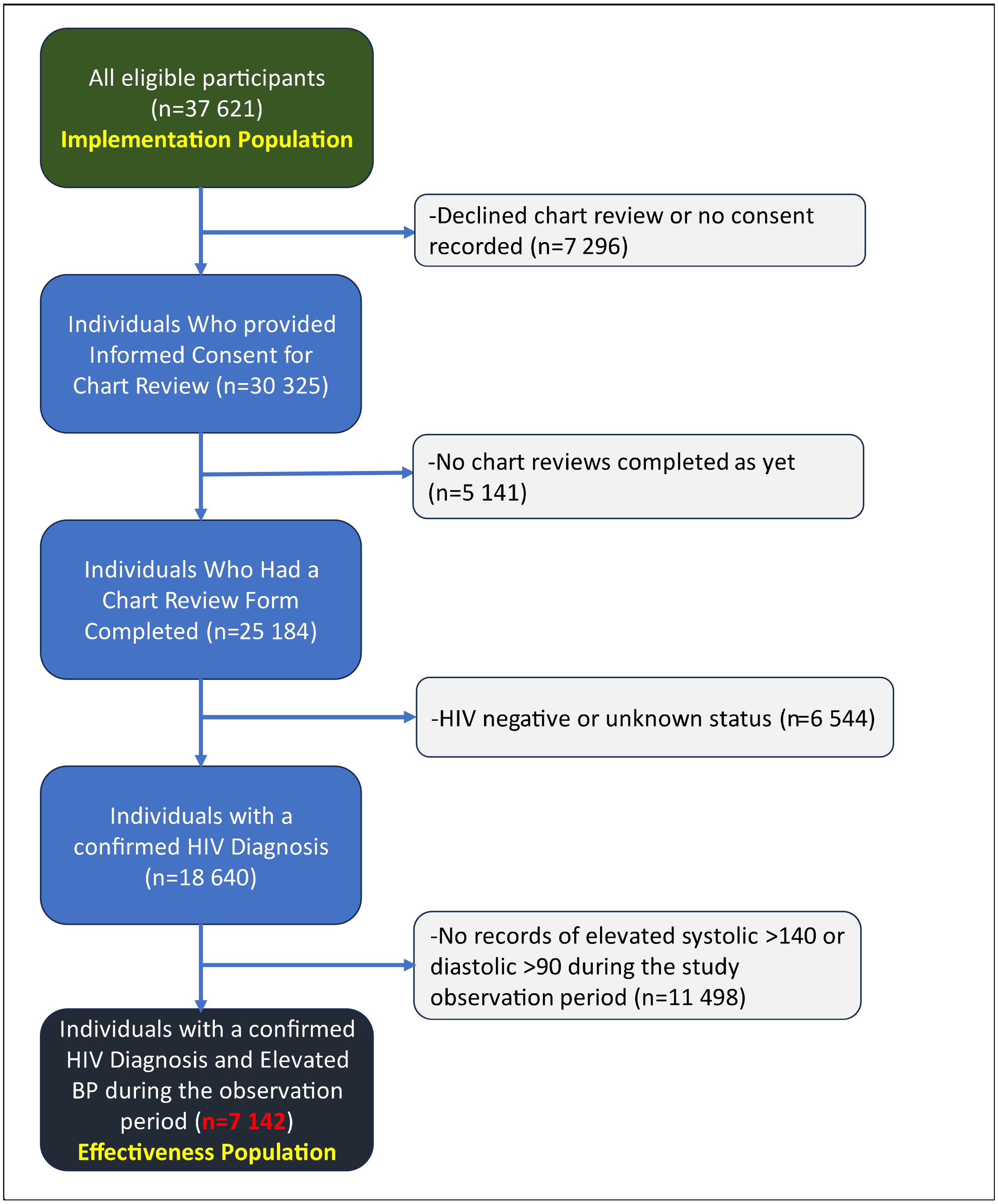

Figure 1: Consort diagram for the selection of the study participants for the iHEART-SA project (by 31 May 31, 2024)

IHEART-SA PRIMARY OUTCOME

The iHEART-SA study estimates the proportion of persons obtaining sustained HTN control in the intervention versus control clinics, taking into account the stepped-wedge design. HTN is classified as mean systolic BP (SBP) ≥ 140 mmHg, mean diastolic BP (DBP) ≥ 90 mmHg, or a reported prior diagnosis of HTN. iHEART-SA calculates the effect of clinic-level intervention exposure (no intervention versus intervention by study staff) using mixed-effects logistic regression models with fixed effects for intervention exposure status and study step, as well as a random variable for the

clinic. iHEART-SA includes patient demographic and clinical information in adjusted analyses to account for any differences between clinics.

Our study differs from iHEART-SA in that it will include both PLHIV and PNLHIV, allowing for an in-depth assessment and comparison of the transition across various care continuum stages for both groups. Furthermore, while the iHEART-SA project focuses on PLHIV care continuum progression, our study will explore the regression from more advanced to less advanced stages of the continuum, as well as progression along the continuum for both PLHIV and PNLHIV.

STUDY PERIOD

We will extract data from the iHEART-SA database for study participants who were enrolled between August 2022 and May 2024. This was the study recruitment period for the iHEART-SA project, followed by the maintenance phase/period (file reviews of follow-up visits for the enrolled participants) which began in June 2024 and will continue until June 2025. Our study will also include file reviews for the follow-up visits that occurred during the recruitment and maintenance periods, up until September 2024.

STUDY POPULATION AND LOCATION

The iHEART-SA study is conducted in 9 of 14 clinics (Crown Gardens clinic, Esselen clinic, Glenanda clinic, Jeppe clinic, Kibler Park clinic, Malvern clinic, Rosettenville clinic, South Hills clinic, Yeoville clinic) in Region F (Figure 7). These clinics serve almost 700000 residents and offer reproductive and sexual health care, mobile and community outreach, HIV screening and treatment, and tuberculosis screening and treatment. From August 2022 to March 2024, each clinic served an average of 3000 PLHIV who had elevated BP.

The study location, region F (inner-city), possesses particular characteristics that significantly impact the utilization of health services and the execution of programs. The inner city is a constituent region of the City of Johannesburg, which is divided into seven regions. The City of Johannesburg is a district inside the Gauteng Province, which is considered the economic center of the country. The inner city is highly populated, comprising the flatland regions of Hillbrow and Berea, together with the Johannesburg Central Business District. The district is believed to house approximately 15% of the city of Johannesburg's 4 million residents [21]. Nevertheless, this percentage probably exceeds 15% as the figures do not account for the majority of the sizable transient population residing in the inner-city area. The urban area is a distinctively intricate and ever-changing setting that has experienced significant changes in population, social structure, and economy in recent decades. A significant portion of its population consists of immigrants, both South Africans from different provinces within the country and individuals from other countries. Approximately more than 25% of adults in the region are without employment [21]. The urban core contains numerous major cab hubs, and it is estimated that 800,000 commuters traverse the city daily. Informal trading is the prevailing economic activity. Regrettably, the region is afflicted by elevated rates of criminal activity and illicit drug trade [22].

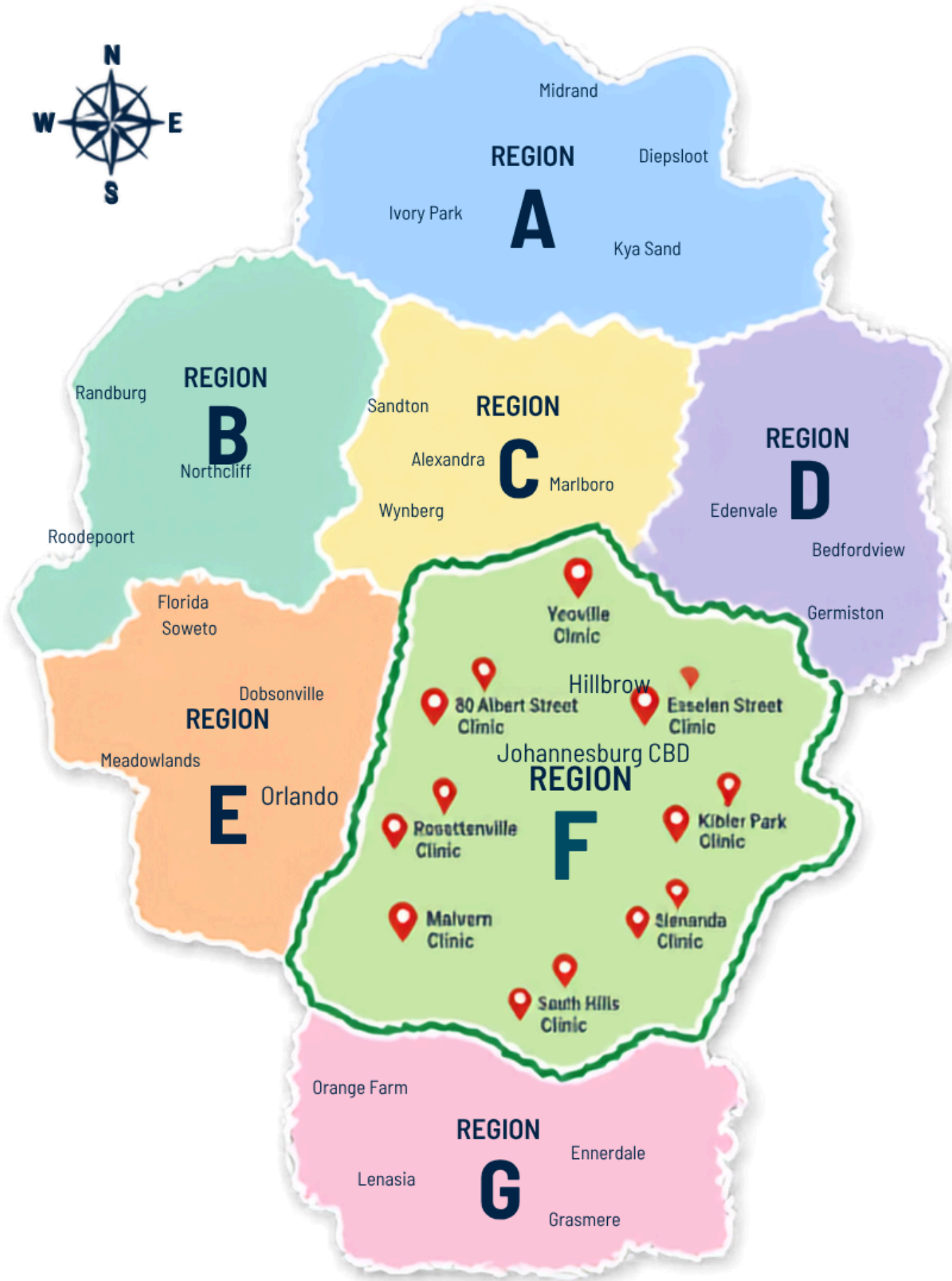

Figure 2: Region F location with study clinics

SAMPLE SIZE

For the analyses, we will include all the eligible data in the dataset. However the minimal sample size (to detect the primary clinical outcome) was calculated as follows: a total study population of PLHIV with HTN and PNLHIV with HTN in the study setting was used, with 85% power and alpha=0.05, assuming the ratio between these populations as 1:3 and 35% HTN prevalence in the overall study population (based on Berry et al., (2017) [22] and unpublished iHEART-SA data) (Table 1), we require a base sample of 519 participants across all clinics (PLHIV=346 and PNLHIV=173).

Table 1: iHEART-SA data disaggregated by BP categories for PLHIV and PNLHIV from August 2022 to May 2024.

**Participant characteristics**	All	**HIV positive**	**HIV negative**
**BP categorization**	**24,846**	19,807 (79.7%)	5,039 (20.3%)
High	**8,567 (34.5%)**	5,739 (29.0%)	2,828 (56.1%)
High-normal	**5,069 (20.4%)**	4,067 (20.5%)	1,002 (19.9%)
Normal	**11,210 (45.1%)**	10,001 (50.5%)	1,209 (23.9%)

DATA, INCLUSION, AND EXCLUSION CRITERIA

The iHEART-SA dataset will be utilized for data extraction (for both PLHIV and PNLHIV). We will consider the measured and recorded BP and HTN diagnosis, treatment, and control information. HTN diagnosis is made after two consecutive elevated BP measures and subsequently is recorded in the medical record/chart. Being on anti-hypertensive treatment is considered as receiving anti-hypertensive medication at study enrolment. Participants were categorized according to possible BP control, defined as a measured systolic BP of less than 140 mmHg and a measured diastolic BP of less than 90 mmHg [23]. We will include all available covariates and exposure variables recorded in the web-based iHEART-SA REDCap dataset during the study period to maximize the comparability and depth of the data obtained. This will include information on sex, age, HIV status, pregnancy status (women), nationality, facility size classification (high, medium, low), substance use, BP measurements, HTN diagnosis, HTN diagnosis date, HTN medication, HTN medication start date, name of HTN medication or regimen, HTN medication dosages, lifestyle modification education or counselling, referral for lifestyle modification services or support, comorbidity information. For PLHIV, CD4 cell count, viral load information, and duration on ART will make up the analytical dataset (Table 2). REDCap tools used in the iHEART-SA study are provided as supporting material in this protocol.

Table 2: iHEART-SA REDCap data collection tools

**iHEART-SA_Enrollment Demographics Form**	This is the form in the iHEART-SA REDcap tools that records enrolment and demographic-related information. This includes first name, last name, date of birth, age, gender, nationality, HIV status, weight and height. It also records information treatment dispensing and distribution models. At the initial visit, this form also serves as a screening tool to ensure that patients younger than 18 years old are categorized accordingly. This form also records systolic and diastolic BP readings at the initial or first visit.
**iHEART-SA Study Consent Form**	In the iHEART-SA study, each participant who is interested in taking part and be enrolled in the study will need to first give consent for study participation. Informed consent means that sufficient information about the study is provided to the potential participants for them to make an informed decision about taking part in the study. The participant needs to understand the information about the study and the implications of acting on that information. Informed consent relates to a person's right to human dignity and autonomy.<br>For patients giving full consent at the initial visit (consent to file review and consent to further research), this form is completed once off. However, it is possible to complete this form at subsequent visits, for those patients who may have declined consent at the initial visit or had partial consent (consenting for either file review or further research, but not both).
**iHEART-SA_Triage Blood Pressure Measurements**	This is the form that records systolic and diastolic BP readings at subsequent or follow-up visits. This includes information such as "BP taken at initial visit (yes/no)", reasons for not taking BP readings, systolic and diastolic BP readings at follow-up visits, BP readings explained to the patient, most recent height, and weight.
**iHEART-SA_Blood Pressure Management File Review Form**	This is the form that records previous BP diagnosis information (if the patient has ever been diagnosed with BP, date of diagnosis, and if there was antihypertensive therapy initiation) and file review of the current visit (was BP reading recorded in the file, was BP listed as a problem in the file, lifestyle modifications and lifestyle modification support services recorded in the file). This form also records current antihypertensive therapy options and comorbidity information (if the patient has other chronic conditions such as HIV, Mellitus Diabetes, Cholesterol, or Tuberculosis)

OUTCOME MEASUREMENTS

Primary outcome: Estimates will be shown for each outcome (progression and regression) based on the covariates of interest and the exposure variable (HIV status). "Progression" is the movement of individuals from their baseline stage into a more advanced stage of the hypertension care continuum. Once BP control is achieved, no further progression along the continuum is possible.
"Regression" is the backward movement of individuals from their baseline stage into a prior stage of the hypertension care continuum. Once study subjects have been diagnosed, they can no longer be undiagnosed.

Secondary outcome: As defined in the iHEART-SA project, individuals are classified as hypertensive if they meet any of the following conditions: mean SBP ≥ 140 mmHg, mean DBP ≥ 90 mmHg, or a reported prior diagnosis of HTN (including self-reported HTN). Our study will also adopt this outcome measurements used in the iHEART-SA project.

STATISTICAL ANALYSES

We will describe and compare individuals' transition through the continuum during the study period by reporting the progression and/or regression of HTN care across all important clinical transitions: undiagnosed, becoming diagnosed (among all those who are undiagnosed), becoming treated (among all those who are untreated), becoming treated and controlled (among all those with uncontrolled BP), stopping treatment (among all those who report taking treatment during the study period), and losing control (among all those with treated and controlled BP). We will conduct compartmental modeling across various care continuum stages for both groups over time. We will report the frequencies, proportions, and probabilities of individuals in the data that made each of the transitions (Figure 2).

We will also investigate whether certain socio-demographic subgroups are at an increased risk of not moving forward through the continuum or moving backward to prior stages. We will compare and report the differences based on age group, sex, nationality, facility size classification (high, medium, low), and substance use. To estimate the associations, we will use the univariate analysis, chi-squared, Fisher's Exact and student t-tests. To calculate the risk magnitude of differences and adjust for different exposures or distributions across the continuum stages, we will use regression models (Poisson and/or logistic) for progression and regression through the continuum with each sociodemographic characteristic as the main exposure.

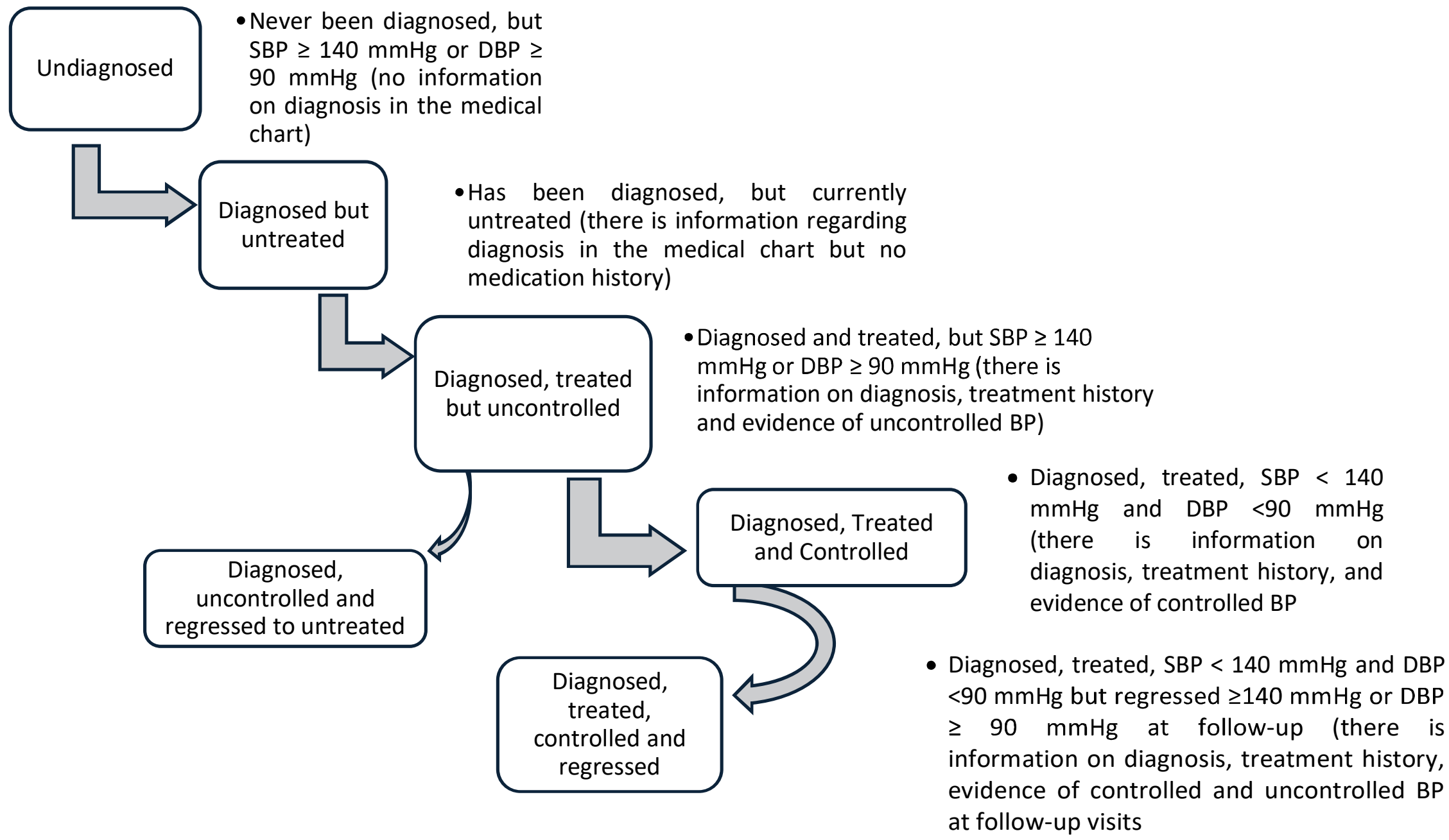

Figure 2. Stages of the hypertension care continuum for the study population

ETHICAL CONSIDERATIONS

This will be a sub-study of the iHEART-SA study which has received approval from the Human Research Ethics Committee (Medical) of the University of Witwatersrand (M211160) and the Johannesburg District Research Office (NHRD No. GP_202111_015). Approval for this sub-study will also be sought from the Human Research Ethics Committee (Medical) of the University of Witwatersrand before the start of the study.

MENTOR OVERSIGHT

Dr. Gumede will convene regular monthly meetings with the mentors, to evaluate the progress of the study and discuss challenges and opportunities additional to the on-site support from Lalla-Edward as required.

DATA INTEGRITY

We will comply with the current iHEART-SA Standard Operating Procedures (SOPs) and report regularly to the study team (including IHEART-SA PIs), addressing data issues in real time.

GENERAL DATA PROTECTION

We have strict procedures in place to protect the privacy and confidentiality of patient records. We will utilize the following protections:

a. Data will be collected, processed, shared, and stored in accordance with provisions of the **Protection of Personal Information Act 4 of 2013** (as amended) and with the research protocol/proposal as approved by the Wits HREC (Medical);
b. All study personnel are trained in procedures to minimize the potential for breaches of confidentiality, including but not limited to ensuring that all files are closed and that no conversations about participants occur in public settings.
c. All names and potential identifiers will be omitted from study records, and replaced with assigned numeric study identifiers; however, these are not meaningful to anyone without access to the original study logs. All data files are maintained under password protection at all times.
d. The study employs password-protection for computer data files, and locked file cabinets in restricted-access buildings. Regular data back-up schedules and appropriate server security procedures (to ward off unauthorized data retrieval attempts) are already instituted.
e. Analyses of data and publication of manuscripts will be conducted in a manner such that identification of individuals will be impossible.

DISSEMINATION

Over the course of the project, we will share our findings with relevant stakeholders, produce all required reports, and present at scientific conferences (e.g.SAAIDS). The manuscript *(draft title: Progression and regression through the HTN care continuum: comparisons between people living with HIV (PLHIV) and those without HIV (PNLHIV) from the clinic-based clinical trial data in South Africa)* will be submitted to peer-reviewed journals (potential journals: International Journal of Chronic Diseases, Journal of Hypertension, Journal of Epidemiology and Global Health). We have budgeted for open access journal fees to support this project.

TIMELINES

Table 3: Timelines for the planned study-related activities for the duration of the study

**Activities**	Oct-24	Nov-24	Dec-24	Jan-25	Feb-25	Mar-25	Apr-25	May-25	Jun-25	Jul-25	Aug-25	Sep-25
Regulatory processes												
Data activities for Aim 1												
Data activities for Aim 2												
Statistical analysis of all data												
Manuscript development												
Dissemination outputs for conferences and journals												
Grant report summary and submission												

FUNDING

This study is funded by the HLB-SIMPLe Alliance as a Small Research Project grant.

REGULATION STATEMENT

This study will be conducted in accordance with the ethical principles of:

- World Medical Association Declaration of Helsinki
- Council for International Organizations of Medical Sciences
- ICH GCP guidelines
- Applicable national ethics and regulatory requirements in countries where the study is being conducted, e.g. Guidelines for Good Practice in the Conduct of Clinical Trials in Human Participants in South Africa.

STUDY TEAM

Dr. Gumede (Researcher at Ezintsha) will lead the implementation of the project under the mentorship of South African-based Dr. Lalla-Edward (Director of Research Strategy and Integrating HIV and Heart Health in South Africa (iHEART-SA) co-investigator), and Prof. Manne-Goehler based in the United States. In addition, Dr. Elizabeth Oladimeji, a mixed methods researcher who leads the qualitative components of Ezintsha studies, will be a co-investigator in this project.

REFERENCE

1. Gazzaruso C, Bruno R, Garzaniti A, Giordanetti S, Fratino P, Sacchi P, et al. Hypertension among HIV patients: Prevalence and relationships to insulin resistance and metabolic syndrome. J Hypertens. 2003;21(7). doi 10.1097/00004872-200307000-00028
2. Peck RN, Shedafa R, Kalluvya S, Downs JA, Todd J, Suthanthiran M, et al. Hypertension, kidney disease, HIV and antiretroviral therapy among Tanzanian adults: A cross-sectional study. BMC Med. 2014;12(1). doi 10.1186/s12916-014-0125-2
3. Daniels A, Huffman M, Lombard C, Steyn K, Levitt NS, Katzenellenbogen J. Blood pressure and social support observations from Mamre, South Africa, during social and political transition. J Hum Hypertens. 1999;13(10). doi 10.1038/sj.jhh.1000892
4. Metcalf CA, Hoffman MN, Steyn K, Katzenellenbogen JM, Fourie JM. Design and baseline characteristics of a hypertension intervention program in a South African village. J Hum Hypertens. 1996;10(1).
5. Steyn K, Gaziano TA, Bradshaw D, Laubscher R, Fourie J. Hypertension in South African adults: Results from the Demographic and Health survey, 1998. J Hypertens. 2001;19(10). doi 10.1097/00004872-200110000-00004
6. National Department of Health. South Africa Demographic and health survey. Natl Dep Heal [Internet]. 2016; Available from: https://dhsprogram.com/pubs/pdf/FR337/FR337.pdf. https://dhsprogram.com/pubs/pdf/FR337/FR337.pdf
7. Xu X, Lin H, Chen X, Zhu B, Shen W, Ning C, et al. Differences in hypertension and prehypertension among people living with and without HIV in China: role of HIV infection and antiretroviral therapy. HIV Med. 2021;22(5). doi 10.1111/hiv.13040
8. Tsuro U, Oladimeji KE, Pulido-Estrada GA, Apalata TR. Risk Factors Attributable to Hypertension among HIV-Infected Patients on Antiretroviral Therapy in Selected Rural Districts of the Eastern Cape Province, South Africa. Int J Environ Res Public Health. 2022;19(18). doi 10.3390/ijerph191811196
9. Dillon DG, Gurdasani D, Riha J, Ekoru K, Asiki G, Mayanja BN, et al. Association of HIV and ART with cardiometabolic traits in sub-Saharan Africa: A systematic review and meta-analysis. Int J Epidemiol. 2013;42(6). doi 10.1093/ije/dyt198
10. Okyere J, Ayebeng C, Owusu BA, Dickson KS. Prevalence and factors associated with hypertension among older people living with HIV in South Africa. BMC Public Health. 2022;22(1). doi 10.1186/s12889-022-14091-y
11. Chiwandire N, Zungu N, Mabaso M, Chasela C. Trends, prevalence and factors associated with hypertension and diabetes among South African adults living with HIV, 2005–2017. BMC Public Health. 2021;21(1). doi 10.1186/s12889-021-10502-8
12. Ware LJ, Chidumwa G, Charlton K, Schutte AE, Kowal P. Predictors of hypertension awareness, treatment and control in South Africa: results from the WHO-SAGE population survey (Wave 2). J Hum Hypertens. 2019;33(2). doi 10.1038/s41371-018-0125-3
13. Harimenshi D, Niyongabo T, Preux PM, Aboyans V, Desormais I. Hypertension and associated factors in HIV-infected patients receiving antiretroviral treatment in Burundi: a cross-sectional study. Sci Rep. 2022;12(1). doi 10.1038/s41598-022-24997-7
14. Todowede OO, Sartorius B, Magula N, Schutte AE. Association of predicted 10 years cardiovascular mortality risk with duration of HIV infection and antiretroviral therapy among HIV-infected individuals in Durban, South Africa. Diabetol Metab Syndr. 2019;11(1). doi

10.1186/s13098-019-0502-2

15. Fiseha T, Belete AG, Dereje H, Dires A. Hypertension in HIV-Infected Patients Receiving Antiretroviral Therapy in Northeast Ethiopia. Int J Hypertens. 2019;2019. doi 10.1155/2019/4103604
16. World Health Organization (WHO). Hypertension. 2023 [cited 2023 Apr 24]; Available from: https://www.who.int/news-room/fact-sheets/detail/hypertensiondoi doi: 10.5830/CVJA-2022-065. https://www.who.int/news-room/fact-sheets/detail/hypertension
17. Ferdinand KC. Uncontrolled hypertension in sub-Saharan Africa: Now is the time to address a looming crisis. Vol. 22, Journal of Clinical Hypertension. 2020. doi 10.1111/jch.14046
18. Geldsetzer P, Manne-Goehler J, Marcus ME, Ebert C, Zhumadilov Z, Wesseh CS, et al. The state of hypertension care in 44 low-income and middle-income countries: a cross-sectional study of nationally representative individual-level data from 1·1 million adults. Lancet. 2019;394(10199). doi 10.1016/S0140-6736(19)30955-9
19. Kohli-Lynch CN, Erzse A, Rayner B, Hofman KJ. Hypertension in the South African public healthcare system: A cost-of-illness and burden of disease study. BMJ Open. 2022;12(2). doi 10.1136/bmjopen-2021-055621
20. Chow CK, Teo KK, Rangarajan S, Islam S, Gupta R, Avezum A, et al. Prevalence, awareness, treatment, and control of hypertension in rural and urban communities in high-, middle-, and low-income countries. JAMA. 2013;310(9). doi 10.1001/jama.2013.184182
21. Wozniak G, Khan T, Gillespie C, Sifuentes L, Hasan O, Ritchey M, et al. Hypertension Control Cascade: A Framework to Improve Hypertension Awareness, Treatment, and Control. J Clin Hypertens. 2016;18(3). doi 10.1111/jch.12654
22. Berry KM, Parker WA, McHiza ZJ, Sewpaul R, Labadarios D, Rosen S, et al. Quantifying unmet need for hypertension care in South Africa through a care cascade: Evidence from the SANHANES, 2011-2012. Vol. 2, BMJ Global Health. 2017. doi 10.1136/bmjgh-2017-000348
23. Seedat YK, Rayner BL, Veriava Y. South African hypertension practice guideline 2014. Vol. 25, Cardiovascular Journal of Africa. 2014. doi 10.5830/CVJA-2014-062
