# Supplementary material for: The longitudinal care cascade for hypertension in people with and without HIV in South Africa: A clinic-based observational study
Source: PLoS Med. 2026 Sep 11;23(9):e1004957. doi: 10.1371/journal.pmed.1004957 (PMC13567817; doi:10.1371/journal.pmed.1004957)
Supplement: Supplementary file 3 [file pmed.1004957.s005.pdf]

```
import delimited "C:\Users\SiphamandlaGumede\OneDrive - Wits Health Consortium\Ezintsha PC\Desktop\PHD\iHEART SA\SRPs\Spha 2024\Dataset and analysis\iheart dataset all patients.csv"


gen agecat=.
replace agecat=1 if ageinyears <30
replace agecat=2 if ageinyears >=30 & ageinyears <=39
replace agecat=3 if ageinyears >=40 & ageinyears <=49
replace agecat=4 if ageinyears >=50 & ageinyears <=59
replace agecat=5 if ageinyears >=60 & ageinyears!=.

label define ageinyears 1 "between 18-29 years" 2 "Between 30-39" 3 "Between 40-49" 4 "Between 50-59 years" 5 "60+ years", replace
label values agecat ageinyears

ta agecat

gen agecat4=.
replace agecat4=1 if ageinyears <40
replace agecat4=2 if ageinyears >=40 & ageinyears <=59
replace agecat4=3 if ageinyears >=60 & ageinyears!=.

label define ageinyears 1 "between 18-39 years" 2 "Between 40-59" 3 "60+ years", replace
label values agecat4 ageinyears

ta agecat4

gen agecat_collapsed=.
replace agecat_collapsed=2 if ageinyears <39
replace agecat_collapsed=1 if ageinyears >=40 & ageinyears <=49
replace agecat_collapsed=3 if ageinyears >=50 & ageinyears <=59
replace agecat_collapsed=4 if ageinyears >=60 & ageinyears!=.

label define ageinyears 2 "between 18-39 years" 1 "Between 40-49" 3 "Between 50-59 years" 4 "60+ years", replace
label values agecat_collapsed ageinyears

ta agecat_collapsed

*tab agecat_collapsed Diagnosedbutuntreated, row

*bys agecat_collapsed: egen total = count(Diagnosedbutuntreated)
*bys agecat_collapsed: egen events = total(Diagnosedbutuntreated)

*list agecat_collapsed events total if _n == 1, sepby(agecat_collapsed)



gen sex2=.
replace sex2=1 if sex=="Female"
replace sex2=2 if sex=="Male"

label define sex 1 "Female" 2 "Male", replace
label values sex2 sex

ta sex2

gen nationality2=.
replace nationality2=1 if nationality=="South Africa"
replace nationality2=2 if nationality=="Zimbabwe"
replace nationality2=3 if nationality=="Botswana"
replace nationality2=3 if nationality=="DRC"
replace nationality2=3 if nationality=="Ethiopia"
```

```
replace nationality2=3 if nationality=="Lesotho"
replace nationality2=3 if nationality=="Malawi"
replace nationality2=3 if nationality=="Mozambique"
replace nationality2=3 if nationality=="Nigeria"
replace nationality2=3 if nationality=="Other"
replace nationality2=3 if nationality=="Somalia"
replace nationality2=3 if nationality=="Swaziland"
replace nationality2=3 if nationality=="Tanzania"
replace nationality2=3 if nationality=="Zambia"

label define nationality 1 "South Africa" 2 "Zimbabwe" 3 "Other", replace
label values nationality2 nationality

ta nationality2

gen bpcat=.
replace bpcat=1 if systolicbpmeasurementatinitialvi <=129
replace bpcat=2 if systolicbpmeasurementatinitialvi >=130 & systolicbpmeasurementatinitialvi <=139
replace bpcat=3 if systolicbpmeasurementatinitialvi >=140 & systolicbpmeasurementatinitialvi!=.

label define systolicbpmeasurementatinitialvi 1 "Normal" 2 "High-normal" 3 "High", replace
label values bpcat systolicbpmeasurementatinitialvi

ta bpcat


gen bpcat2=.
replace bpcat2=1 if systolicbpmeasurementatcurrentvi <=129
replace bpcat2=2 if systolicbpmeasurementatcurrentvi >=130 & systolicbpmeasurementatcurrentvi <=139
replace bpcat2=3 if systolicbpmeasurementatcurrentvi >=140 & systolicbpmeasurementatcurrentvi!=.

label define systolicbpmeasurementatcurrentvi 1 "Normal" 2 "High-normal" 3 "High", replace
label values bpcat2 systolicbpmeasurementatcurrentvi

gen filereviewdone=.

replace filereviewdone=1 if previouslydiagnosedwithbp=="Yes"
replace filereviewdone=1 if previouslydiagnosedwithbp=="No"
replace filereviewdone=0 if previouslydiagnosedwithbp=="."

label define previouslydiagnosedwithbp 1 "Yes" 0 "No", replace
label values filereviewdone previouslydiagnosedwithbp

ta filereviewdone

gen HIVstatus2=.
replace HIVstatus2=1 if anyothercormobiditieschoicehiv=="Checked"
replace HIVstatus2=0 if anyothercormobiditieschoicehiv=="Unchecked"

label define anyothercormobiditieschoicehiv 1 "HIV+" 0 "HIV-", replace
label values HIVstatus2 anyothercormobiditieschoicehiv

ta HIVstatus2

ta agecat_collapsed hivstatus if consentedtoaccessingclinicalinfo=="Yes" & hivstatus!="Unknown" &
filereviewdone==1, row col
ta sex2 hivstatus if consentedtoaccessingclinicalinfo=="Yes" & hivstatus!="Unknown" & filereviewdone==
1, row col
ta nationality2 hivstatus if consentedtoaccessingclinicalinfo=="Yes" & hivstatus!="Unknown" &
filereviewdone==1, row col

su ageinyears if consentedtoaccessingclinicalinfo=="Yes" & filereviewdone==1, d
su ageinyears if hivstatus=="HIV positive" & consentedtoaccessingclinicalinfo=="Yes" & filereviewdone
```

```
==1, d
su ageinyears if hivstatus=="HIV negative" & consentedtoaccessingclinicalinfo=="Yes" & filereviewdone==1, d


su systolicbpmeasurementatinitialvi if consentedtoaccessingclinicalinfo=="Yes" & filereviewdone==1, d
su systolicbpmeasurementatinitialvi if hivstatus=="HIV positive" & consentedtoaccessingclinicalinfo=="Yes" & filereviewdone==1, d
su systolicbpmeasurementatinitialvi if hivstatus=="HIV negative" & consentedtoaccessingclinicalinfo=="Yes" & filereviewdone==1, d

su diastolicbpmeasurementatinitialv if consentedtoaccessingclinicalinfo=="Yes" & filereviewdone==1, d
su diastolicbpmeasurementatinitialv if hivstatus=="HIV positive" & consentedtoaccessingclinicalinfo=="Yes" & filereviewdone==1, d
su diastolicbpmeasurementatinitialv if hivstatus=="HIV negative" & consentedtoaccessingclinicalinfo=="Yes" & filereviewdone==1, d

ta bpcat hivstatus if consentedtoaccessingclinicalinfo=="Yes" & hivstatus!="Unknown" & filereviewdone==1, row col

drop if hivstatus=="Unknown"
ta filereviewdone hivstatus
ta hivstatus if consentedtoaccessingclinicalinfo=="Yes" & filereviewdone==1
ta bpcat hivstatus if consentedtoaccessingclinicalinfo=="Yes" & filereviewdone==1
ta bpcat hivstatus if consentedtoaccessingclinicalinfo=="Yes" & filereviewdone==1 & previouslydiagnosedwithbp=="No"
ta bpcat hivstatus if consentedtoaccessingclinicalinfo=="Yes" & filereviewdone==1 & previouslydiagnosedwithbp=="Yes"
ta bpcat hivstatus if consentedtoaccessingclinicalinfo=="Yes" & filereviewdone==1 & previouslydiagnosedwithbp=="Yes" & previouslyinitiatedonhtntherapy=="No"
ta bpcat hivstatus if consentedtoaccessingclinicalinfo=="Yes" & filereviewdone==1 & bloodpressuremedicinesprescribed=="No"
ta bpcat hivstatus if consentedtoaccessingclinicalinfo=="Yes" & filereviewdone==1 & previouslydiagnosedwithbp=="Yes" & bloodpressuremedicinesprescribed=="No"
ta bpcat hivstatus if consentedtoaccessingclinicalinfo=="Yes" & filereviewdone==1 & previouslydiagnosedwithbp=="Yes" & bloodpressuremedicinesprescribed=="Yes"
ta bpcat hivstatus if consentedtoaccessingclinicalinfo=="Yes" & filereviewdone==1 & bloodpressuremedicinesprescribed=="Yes"

ta bpcat2 anyothercormobiditieschoicehiv if previouslydiagnosedwithbp=="Yes" & bloodpressuremedicinesprescribed=="Yes"
ta bpcat2 anyothercormobiditieschoicehiv if previouslydiagnosedwithbp=="No" & bloodpressuremedicinesprescribed=="Yes"
ta bpcat2 HIVstatus2 if previouslydiagnosedwithbp=="Yes" & bloodpressuremedicinesprescribed=="Yes" & eventname=="Visit 2"
ta bpcat2 anyothercormobiditieschoicehiv

ta bpcat hivstatus if consentedtoaccessingclinicalinfo=="Yes" & filereviewdone==1 & previouslydiagnosedwithbp=="No"
ta bpcat hivstatus if consentedtoaccessingclinicalinfo=="Yes" & filereviewdone==1 & previouslydiagnosedwithbp=="Yes" & bloodpressuremedicinesprescribed=="No"
ta bpcat hivstatus if consentedtoaccessingclinicalinfo=="Yes" & filereviewdone==1 & bloodpressuremedicinesprescribed=="No"
ta bpcat hivstatus if consentedtoaccessingclinicalinfo=="Yes" & filereviewdone==1 & previouslydiagnosedwithbp=="Yes"
ta bpcat hivstatus if consentedtoaccessingclinicalinfo=="Yes" & filereviewdone==1
ta bpcat hivstatus if consentedtoaccessingclinicalinfo=="Yes" & filereviewdone==1 & bloodpressuremedicinesprescribed=="Yes"

ta bpcat2 anyothercormobiditieschoicehiv if previouslydiagnosedwithbp=="Yes" & bloodpressuremedicinesprescribed=="Yes"
ta bpcat2 anyothercormobiditieschoicehiv if previouslydiagnosedwithbp=="Yes" & bloodpressuremedicinesprescribed=="No"
```

```
ta bpcat2 HIVstatus2 if previouslydiagnosedwithbp=="Yes" & bloodpressuremedicinesprescribed=="No"
ta bpcat2 HIVstatus2 if previouslydiagnosedwithbp=="Yes" & previouslyinitiatedonhtntherapy=="Yes" & bloodpressuremedicinesprescribed=="No"
ta bpcat2 HIVstatus2 if previouslydiagnosedwithbp=="Yes" & previouslyinitiatedonhtntherapy=="Yes" & bloodpressuremedicinesprescribed=="Yes" & bloodpressureorhypertensionliste=="Yes"
ta bpcat HIVstatus2 if previouslydiagnosedwithbp=="Yes" & previouslyinitiatedonhtntherapy=="Yes" & bloodpressuremedicinesprescribed=="Yes" & bloodpressureorhypertensionliste=="Yes"

ta bpcat2 HIVstatus2 if previouslydiagnosedwithbp=="Yes" & bloodpressuremedicinesprescribed=="Yes" & eventname=="Visit 4"
ta bpcat2 HIVstatus2 if previouslydiagnosedwithbp=="Yes" & bloodpressuremedicinesprescribed=="Yes" & eventname=="Visit 5"
ta bpcat2 HIVstatus2 if previouslydiagnosedwithbp=="Yes" & bloodpressuremedicinesprescribed=="Yes" & eventname=="Visit 7"
ta bpcat2 HIVstatus2 if previouslydiagnosedwithbp=="Yes" & bloodpressuremedicinesprescribed=="Yes" & eventname=="Visit 10"

ta bpcat hivstatus if eventname=="Initial Visit"

ta bpcat2 hivstatus if consentedtoaccessingclinicalinfo=="Yes" & filereviewdone==1 & bloodpressuremedicinesprescribed=="Yes"
ta bpcat2 hivstatus if filereviewdone==1 & bloodpressuremedicinesprescribed=="Yes"
ta bpcat2 HIVstatus2 if filereviewdone==1 & bloodpressuremedicinesprescribed=="Yes"
ta bpcat hivstatus if consentedtoaccessingclinicalinfo=="Yes" & filereviewdone==1 & previouslydiagnosedwithbp=="Yes"
ta previouslyinitiatedonhtntherapy

ta bpcat hivstatus if previouslydiagnosedwithbp=="Yes" & previouslyinitiatedonhtntherapy=="Yes" & bloodpressuremedicinesprescribed=="Yes" & bloodpressureorhypertensionliste=="Yes"
ta bpcat hivstatus if previouslydiagnosedwithbp=="Yes" & previouslyinitiatedonhtntherapy=="Yes" & bloodpressuremedicinesprescribed=="Yes"
ta bpcat hivstatus if previouslyinitiatedonhtntherapy=="Yes" & bloodpressuremedicinesprescribed=="Yes"
ta bpcat hivstatus if previouslydiagnosedwithbp=="Yes" & previouslyinitiatedonhtntherapy=="Yes"
ta bpcat hivstatus if previouslyinitiatedonhtntherapy=="Yes"
ta bpcat2 hivstatus if filereviewdone==1 & bloodpressuremedicinesprescribed=="Yes"
ta bpcat2 HIVstatus2 if filereviewdone==1 & bloodpressuremedicinesprescribed=="Yes"

ta bpcat2 HIVstatus2 if previouslydiagnosedwithbp=="Yes" & previouslyinitiatedonhtntherapy=="Yes" & bloodpressuremedicinesprescribed=="No" & eventname=="Visit 3"
ta bpcat2 HIVstatus2 if previouslydiagnosedwithbp=="Yes" & previouslyinitiatedonhtntherapy=="Yes" & bloodpressuremedicinesprescribed=="No" & eventname=="Visit 5"
ta bpcat2 HIVstatus2 if previouslydiagnosedwithbp=="Yes" & previouslyinitiatedonhtntherapy=="Yes" & bloodpressuremedicinesprescribed=="No" & eventname=="Visit 2" & & eventname=="Visit 3"

ta bpcat2 HIVstatus2 if previouslydiagnosedwithbp=="Yes" & previouslyinitiatedonhtntherapy=="Yes" & eventname=="Visit 9"

ta bpcat2 HIVstatus2 if previouslydiagnosedwithbp=="Yes" & bloodpressuremedicinesprescribed=="Yes" & eventname=="Visit 7"
ta bpcat hivstatus if consentedtoaccessingclinicalinfo=="Yes" & filereviewdone==1 & previouslydiagnosedwithbp=="Yes" & previouslyinitiatedonhtntherapy=="No"

************Test of association*****************
ttest ageinyears if consentedtoaccessingclinicalinfo=="Yes" & hivstatus!="Unknown" & filereviewdone==1 , by ( hivstatus )
ta agecat hivstatus if consentedtoaccessingclinicalinfo=="Yes" & hivstatus!="Unknown" & filereviewdone==1, row col chi2
ta sex2 hivstatus if consentedtoaccessingclinicalinfo=="Yes" & hivstatus!="Unknown" & filereviewdone==1, row col chi2
ta nationality2 hivstatus if consentedtoaccessingclinicalinfo=="Yes" & hivstatus!="Unknown" & filereviewdone==1, row col chi2
ttest systolicbpmeasurementatinitialvi if consentedtoaccessingclinicalinfo=="Yes" & hivstatus!="Unknown" & filereviewdone==1, by ( hivstatus )
```

```
ttest diastolicbpmeasurementatinitialv if consentedtoaccessingclinicalinfo=="Yes" & hivstatus!="Unknown" & filereviewdone==1, by ( hivstatus )
ta bpcat2 hivstatus if consentedtoaccessingclinicalinfo=="Yes" & hivstatus!="Unknown" & filereviewdone==1, row col chi2


*****************logistic or relative regression**************
poisson progressed i.agecat i.sex2 i.nationality2, vce(robust) irr
poisson progressed i.agecat i.sex2 i.nationality2 if hivstatus=="HIV negative", vce(robust) irr
poisson progressed i.agecat i.sex2 i.nationality2 if hivstatus=="HIV positive", vce(robust) irr

poisson regressed i.agecat i.sex2 i.nationality2, vce(robust) irr
poisson regressed i.agecat i.sex2 i.nationality2 if hivstatus=="HIV negative", vce(robust) irr
poisson regressed i.agecat i.sex2 i.nationality2 if hivstatus=="HIV positive", vce(robust) irr

poisson progressed i.HIVstatus2 i.agecat i.sex2 i.nationality2, vce(robust) irr
poisson regressed i.HIVstatus2 i.agecat i.sex2 i.nationality2, vce(robust) irr

*******************new regression models**************************
* 1. Inspect coding (always run these first)
describe bpcat previouslydiagnosedwithbp
tabulate bpcat, missing
tabulate previouslydiagnosedwithbp, missing

* --------------------------------------------------
* 2. Create Undiagnosed flag
* Assumptions below:
* - bpcat is numeric and "high" is coded as 3. If 'tabulate bpcat' shows a different code for "high",
*   replace 3 with that code in the replace(...) line.
* - previouslydiagnosedwithbp is string with values like "Yes"/"No"
* --------------------------------------------------
******outcome variable: 1) Undiagnosed, 2) Diagnosed but untreated, 3) Treated Uncontrolled and 4) Treated Control. For regression: 1) Treated uncontrolled regress to untreated, 2) Treated controlled regress to untreated and 3) Treated controlled regress to uncontrol.


* create binary an Undiagnosed variable (byte to save space)
gen byte Undiagnosed = .
replace Undiagnosed = 1 if bpcat == 3 & previouslydiagnosedwithbp == "No"
replace Undiagnosed = 0 if bpcat == 3 & previouslydiagnosedwithbp == "Yes"
tab Undiagnosed if bpcat == 3, missing

gen byte Diagnosedbutuntreated = .
replace Diagnosedbutuntreated = 1 if previouslydiagnosedwithbp == "Yes" ///
    & previouslyinitiatedonhtntherapy == "No"
replace Diagnosedbutuntreated = 0 if previouslydiagnosedwithbp == "Yes" ///
    & previouslyinitiatedonhtntherapy == "Yes"
tab Diagnosedbutuntreated previouslydiagnosedwithbp, missing
tab Diagnosedbutuntreated previouslyinitiatedonhtntherapy, missing

gen byte Diagnosedandtreated = .
replace Diagnosedandtreated = 1 if previouslydiagnosedwithbp == "Yes" ///
    & previouslyinitiatedonhtntherapy == "Yes"
replace Diagnosedandtreated = 0 if previouslydiagnosedwithbp == "Yes" ///
    & previouslyinitiatedonhtntherapy == "No"
tab Diagnosedandtreated previouslydiagnosedwithbp, missing
tab Diagnosedandtreated previouslyinitiatedonhtntherapy, missing


gen byte Treatedandcontrolled = .
replace Treatedandcontrolled = 1 if previouslyinitiatedonhtntherapy == "Yes" ///
    & systolicbpmeasurementatcurrentvi < 140 ///
    & diastolicbpmeasurementatcurrentv < 90
```

```
replace Treatedandcontrolled = 0 if previouslyinitiatedonhtntherapy == "Yes" ///
    & (systolicbpmeasurementatcurrentvi >= 140 ///
       | diastolicbpmeasurementatcurrentv >= 90)
tab Treatedandcontrolled if previouslyinitiatedonhtntherapy == "Yes", missing

tab Treatedandcontrolled if previouslyinitiatedonhtntherapy == "Yes" ///
    & systolicbpmeasurementatcurrentvi < 140 ///
    & diastolicbpmeasurementatcurrentv < 90

count if previouslyinitiatedonhtntherapy == "Yes" ///
    & systolicbpmeasurementatcurrentvi < 140 ///
    & diastolicbpmeasurementatcurrentv < 90
describe systolicbpmeasurementatcurrentvi diastolicbpmeasurementatcurrentv
destring systolicbpmeasurementatcurrentvi diastolicbpmeasurementatcurrentv, replace
summ systolicbpmeasurementatcurrentvi if previouslyinitiatedonhtntherapy == "Yes"
summ diastolicbpmeasurementatcurrentv if previouslyinitiatedonhtntherapy == "Yes"

count if previouslyinitiatedonhtntherapy == "Yes" ///
    & !missing(systolicbpmeasurementatcurrentvi, diastolicbpmeasurementatcurrentv)
tab previouslyinitiatedonhtntherapy ///
    if systolicbpmeasurementatcurrentvi < 140 ///
    & diastolicbpmeasurementatcurrentv < 90


count if previouslyinitiatedonhtntherapy == "Yes" ///
    & systolicbpmeasurementatcurrentvi < 140 ///
    & diastolicbpmeasurementatcurrentv < 90

describe systolicbpmeasurementatcurrentvi diastolicbpmeasurementatcurrentv

*********************888working on collapsing dataset*******transposing*********
describe dateofvisit v105

gen dateofvisit_d = date(dateofvisit, "YMD")
gen v105_d = date(v105, "YMD")

format dateofvisit_d v105_d %td
describe dateofvisit_d v105_d
list dateofvisit dateofvisit_d ///
     v105 v105_d in 1/10

gen byte visit_type = .
replace visit_type = 0 if !missing(dateofvisit_d) & missing(v105_d)
replace visit_type = 1 if !missing(v105_d)

label define visit_type 0 "Baseline" 1 "Follow-up"
label values visit_type visit_type


tab visit_type

list visitdate v105 in 1/10

bysort recordid (visit_type): gen baseline_diagnosed = previouslydiagnosedwithbp if visit_type == 0
bysort recordid (visit_type): gen baseline_treated   = previouslyinitiatedonhtntherapy if visit_type == 0

bysort recordid (visit_type): replace baseline_diagnosed = baseline_diagnosed[_n-1] if missing(baseline_diagnosed)
bysort recordid (visit_type): replace baseline_treated   = baseline_treated[_n-1]   if missing(baseline_treated)

tab baseline_diagnosed
```

```
tab baseline_treated

bysort recordid (visit_type): gen followup_sbp = systolicbpmeasurementatcurrentvi if visit_type == 1
bysort recordid (visit_type): gen followup_dbp = diastolicbpmeasurementatcurrentv  if visit_type == 1

bysort recordid (visit_type): replace followup_sbp = followup_sbp[_n+1] if missing(followup_sbp)
bysort recordid (visit_type): replace followup_dbp = followup_dbp[_n+1] if missing(followup_dbp)

summ followup_sbp followup_dbp

keep if visit_type == 0

gen byte Treatedandcontrolled2 = .

replace Treatedandcontrolled2 = 1 if baseline_treated == "Yes" ///
    & followup_sbp < 140 ///
    & followup_dbp < 90

replace Treatedandcontrolled2 = 0 if baseline_treated == "Yes" ///
    & (followup_sbp >= 140 | followup_dbp >= 90)

tab Treatedandcontrolled2 if baseline_treated == "Yes", missing

*****************
gen followup_treated = previouslyinitiatedonhtntherapy
tab baseline_treated followup_treated

tab bloodpressuremedicinesprescribed, missing
tab bloodpressuremedicinesprescribed visit_type, missing

gen byte followup_treated2 = .
replace followup_treated2 = 1 if bloodpressuremedicinesprescribed == "Yes"
replace followup_treated2 = 0 if bloodpressuremedicinesprescribed == "No"

tab followup_treated2, missing

gen byte Regressed_untreated = .

replace Regressed_untreated = 1 if baseline_treated == "Yes" ///
    & followup_treated2 == 0

replace Regressed_untreated = 0 if baseline_treated == "Yes" ///
    & followup_treated2 == 1

tab Regressed_untreated if baseline_treated == "Yes", missing
tab baseline_treated followup_treated2 if baseline_treated == "Yes"

*************************
gen byte baseline_controlled = .
replace baseline_controlled = 1 if baseline_treated == "Yes" ///
    & systolicbpmeasurementatinitialvi < 140 ///
    & diastolicbpmeasurementatinitialv < 90

replace baseline_controlled = 0 if baseline_treated == "Yes" ///
    & (systolicbpmeasurementatinitialvi >= 140 ///
       | diastolicbpmeasurementatinitialv >= 90)

tab baseline_controlled if baseline_treated == "Yes", missing

gen byte followup_controlled = .

replace followup_controlled = 1 if followup_sbp < 140 ///
    & followup_dbp < 90
```

```
replace followup_controlled = 0 if followup_sbp >= 140 ///
    | followup_dbp >= 90

tab followup_controlled, missing

gen byte Regressed_uncontrolled = .
replace Regressed_uncontrolled = 1 if baseline_controlled == 1 ///
    & followup_controlled == 0
replace Regressed_uncontrolled = 0 if baseline_controlled == 1 ///
    & followup_controlled == 1

tab Regressed_uncontrolled if baseline_controlled == 1, missing
tab baseline_controlled followup_controlled







*gen byte Regressed_untreated = .

*replace Regressed_untreated = 1 if baseline_treated == "Yes" ///
    & followup_treated == "No"

*replace Regressed_untreated = 0 if baseline_treated == "Yes" ///
    & followup_treated == "Yes"

*tab Regressed_untreated if baseline_treated == "Yes", missing
*tab baseline_treated followup_treated if baseline_treated == "Yes"
ta Undiagnosed
ta Diagnosedbutuntreated
ta Diagnosedandtreated
ta Treatedandcontrolled2
ta Regressed_untreated
ta Regressed_uncontrolled


tab HIVstatus2 Undiagnosed, row col
tab agecat_collapsed Undiagnosed, missing row col
tab agecat_collapsed Undiagnosed, row col
tab sex2 Undiagnosed, row col
tab nationality2 Undiagnosed, row col

tab HIVstatus2 Diagnosedbutuntreated, row col
tab agecat_collapsed Diagnosedbutuntreated, missing row col
tab agecat_collapsed Diagnosedbutuntreated, row col
tab sex2 Diagnosedbutuntreated, row col
tab nationality2 Diagnosedbutuntreated, row col

tab HIVstatus2 Diagnosedandtreated, row col
tab agecat_collapsed Diagnosedandtreated, missing row col
tab agecat_collapsed Diagnosedandtreated, row col
tab sex2 Diagnosedandtreated, row col
tab nationality2 Diagnosedandtreated, row col

tab HIVstatus2 Treatedandcontrolled2, row col
tab agecat_collapsed Treatedandcontrolled2, missing row col
tab agecat_collapsed Treatedandcontrolled2, row col
tab sex2 Treatedandcontrolled2, row col
tab nationality2 Treatedandcontrolled2, row col
```

```
tab HIVstatus2 Regressed_untreated, row col
tab agecat_collapsed Regressed_untreated, missing row col
tab agecat_collapsed Regressed_untreated, row col
tab sex2 Regressed_untreated, row col
tab nationality2 Regressed_untreated, row col

tab HIVstatus2 Regressed_uncontrolled, row col
tab agecat_collapsed Regressed_uncontrolled, missing row col
tab agecat_collapsed Regressed_uncontrolled, row col
tab sex2 Regressed_uncontrolled, row col
tab nationality2 Regressed_uncontrolled, row col

tab agecat_collapsed Diagnosedbutuntreated, row
tab agecat_collapsed Diagnosedbutuntreated, row
tab agecat_collapsed Diagnosedbutuntreated, row
tab agecat_collapsed Diagnosedbutuntreated, row


******************* Unadjusted: Undiagnosed******
poisson Undiagnosed i.HIVstatus2, vce(robust) irr

poisson Undiagnosed i.HIVstatus2 ///
    if bpcat == 3, vce(robust) irr

poisson Undiagnosed i.agecat_collapsed ///
    if bpcat == 3, vce(robust) irr

poisson Undiagnosed i.sex2 ///
    if bpcat == 3, vce(robust) irr

poisson Undiagnosed i.nationality2 ///
    if bpcat == 3, vce(robust) irr

poisson Undiagnosed i.agecat_collapsed ///
    if bpcat == 3, vce(robust) irr


******************* Unadjusted: Diagnosed and treated******
poisson Diagnosedandtreated i.HIVstatus2, vce(robust) irr

poisson Diagnosedandtreated i.HIVstatus2 ///
    if bpcat == 3, vce(robust) irr

poisson Diagnosedandtreated i.agecat_collapsed ///
    if bpcat == 3, vce(robust) irr

poisson Diagnosedandtreated i.sex2 ///
    if bpcat == 3, vce(robust) irr

poisson Diagnosedandtreated i.nationality2 ///
    if bpcat == 3, vce(robust) irr

    ******************* Unadjusted: achieved control************
poisson Treatedandcontrolled2 i.HIVstatus2, vce(robust) irr

poisson Treatedandcontrolled2 i.HIVstatus2 ///
    if bpcat == 3, vce(robust) irr

poisson Treatedandcontrolled2 i.agecat_collapsed ///
    if bpcat == 3, vce(robust) irr

poisson Treatedandcontrolled2 i.sex2 ///
    if bpcat == 3, vce(robust) irr
```

```
poisson Treatedandcontrolled2 i.nationality2 ///
    if bpcat == 3, vce(robust) irr

    ******************** Unadjusted: Regressed Untreated************
poisson Regressed_untreated i.HIVstatus2, vce(robust) irr

poisson Regressed_untreated i.HIVstatus2 ///
    if bpcat == 3, vce(robust) irr

poisson Regressed_untreated i.agecat_collapsed ///
    if bpcat == 3, vce(robust) irr

poisson Regressed_untreated i.sex2 ///
    if bpcat == 3, vce(robust) irr

poisson Regressed_untreated i.nationality2 ///
    if bpcat == 3, vce(robust) irr

    ******************** Unadjusted: Regressed_uncontrolled************
poisson Regressed_uncontrolled i.HIVstatus2, vce(robust) irr

poisson Regressed_uncontrolled i.HIVstatus2, vce(robust) irr

poisson Regressed_uncontrolled i.agecat_collapsed, vce(robust) irr

poisson Regressed_uncontrolled i.sex2, vce(robust) irr

poisson Regressed_uncontrolled i.nationality2, vce(robust) irr


******************

poisson Undiagnosed i.HIVstatus2 i.agecat_collapsed i.sex2 i.nationality2 ///
    if bpcat == 3, vce(robust) irr

poisson Diagnosedbutuntreated i.HIVstatus2 i.agecat_collapsed i.sex2 i.nationality2 ///
    if previouslydiagnosedwithbp == "Yes", vce(robust) irr

poisson Diagnosedbutuntreated i.HIVstatus2 i.agecat4 i.sex2 i.nationality2 ///
    if previouslydiagnosedwithbp == "Yes", vce(robust) irr

poisson Diagnosedandtreated i.HIVstatus2 i.agecat_collapsed i.sex2 i.nationality2 ///
    if previouslydiagnosedwithbp == "Yes", vce(robust) irr

poisson Treatedandcontrolled2 i.HIVstatus2 i.agecat_collapsed i.sex2 i.nationality2 ///
    if baseline_treated == "Yes", vce(robust) irr

poisson Regressed_untreated i.HIVstatus2 i.agecat_collapsed i.sex2 i.nationality2 ///
    if baseline_treated == "Yes", vce(robust) irr

poisson Regressed_untreated i.HIVstatus2 i.agecat_collapsed i.sex2 i.nationality2 ///
    if baseline_treated == "Yes", vce(robust) irr

poisson Regressed_uncontrolled i.HIVstatus2 i.agecat_collapsed i.sex2 i.nationality2 ///
    if baseline_controlled == 1, vce(robust) irr

***********************************
gen clinicid = .

replace clinicid = 1 if healthfacilityname == "Crown Gardens clinic"
replace clinicid = 2 if healthfacilityname == "Esselen clinic"
replace clinicid = 3 if healthfacilityname == "Glenanda clinic"
```

```
replace clinicid = 4 if healthfacilityname == "Jeppe clinic"
replace clinicid = 5 if healthfacilityname == "Kibler clinic"
replace clinicid = 6 if healthfacilityname == "Malvern clinic"
replace clinicid = 7 if healthfacilityname == "Rosettenville clinic"
replace clinicid = 8 if healthfacilityname == "South Hills clinic"
replace clinicid = 9 if healthfacilityname == "Yeoville clinic"

poisson Undiagnosed i.HIVstatus2 i.agecat_collapsed i.sex2 i.nationality2 ///
    if bpcat == 3, vce(cluster clinicid) irr

poisson Undiagnosed i.HIVstatus2 i.agecat_collapsed i.sex2 i.nationality2 ///
    if bpcat == 3, vce(cluster clinicid) irr

poisson Diagnosedbutuntreated i.HIVstatus2 i.agecat_collapsed i.sex2 i.nationality2 ///
    if previouslydiagnosedwithbp == "Yes", vce(cluster clinicid) irr

*poisson Diagnosedbutuntreated i.HIVstatus2 i.agecat4 i.sex2 i.nationality2 ///
    if previouslydiagnosedwithbp == "Yes", vce(cluster clinicid) irr

poisson Diagnosedandtreated i.HIVstatus2 i.agecat_collapsed i.sex2 i.nationality2 ///
    if previouslydiagnosedwithbp == "Yes", vce(cluster clinicid) irr

poisson Treatedandcontrolled2 i.HIVstatus2 i.agecat_collapsed i.sex2 i.nationality2 ///
    if baseline_treated == "Yes", vce(cluster clinicid) irr

poisson Regressed_untreated i.HIVstatus2 i.agecat_collapsed i.sex2 i.nationality2 ///
    if baseline_treated == "Yes", vce(cluster clinicid) irr

poisson Regressed_untreated i.HIVstatus2 i.agecat_collapsed i.sex2 i.nationality2 ///
    if baseline_treated == "Yes", vce(cluster clinicid) irr

poisson Regressed_uncontrolled i.HIVstatus2 i.agecat_collapsed i.sex2 i.nationality2 ///
    if baseline_controlled == 1, vce(cluster clinicid) irr


tab agecat Undiagnosed ///
    Diagnosedbutuntreated ///
    Diagnosedandtreated ///
    Treatedandcontrolled2 ///
    Regressed_untreated ///
    Regressed_uncontrolled

table agecat ///
    Undiagnosed ///
    Diagnosedbutuntreated ///
    Diagnosedandtreated ///
    Treatedandcontrolled2 ///
    Regressed_untreated ///
    Regressed_uncontrolled, ///

table agecat, ///
    statistic(sum Undiagnosed) ///
    statistic(sum Diagnosedbutuntreated) ///
    statistic(sum Diagnosedandtreated) ///
    statistic(sum Treatedandcontrolled2) ///
    statistic(sum Regressed_untreated) ///
    statistic(sum Regressed_uncontrolled)


gen one = 1

table agecat, ///
    statistic(sum one) ///
```

```
    statistic(sum Undiagnosed) ///
    statistic(sum Diagnosedbutuntreated) ///
    statistic(sum Diagnosedandtreated) ///
    statistic(sum Treatedandcontrolled2) ///
    statistic(sum Regressed_untreated) ///
    statistic(sum Regressed_uncontrolled)

drop one

table agecat, ///
    statistic(mean Undiagnosed) ///
    statistic(mean Diagnosedbutuntreated) ///
    statistic(mean Diagnosedandtreated) ///
    statistic(mean Treatedandcontrolled2) ///
    statistic(mean Regressed_untreated) ///
    statistic(mean Regressed_uncontrolled)

foreach var in Undiagnosed Diagnosedbutuntreated Diagnosedandtreated ///
               Treatedandcontrolled2 Regressed_untreated Regressed_uncontrolled {

    table agecat `var', statistic(freq)
}

collect layout (agecat) (`var' result)

collect preview


foreach var in Undiagnosed Diagnosedbutuntreated Diagnosedandtreated ///
               Treatedandcontrolled2 Regressed_untreated Regressed_uncontrolled {
    tab agecat `var', missing
}



gen outcome = .
replace outcome = 1 if Undiagnosed == 1
replace outcome = 2 if Diagnosedbutuntreated == 1
replace outcome = 3 if Diagnosedandtreated == 1
replace outcome = 4 if Treatedandcontrolled2 == 1
replace outcome = 5 if Regressed_untreated == 1
replace outcome = 6 if Regressed_uncontrolled == 1

label define outcome_lbl ///
1 "Undiagnosed" ///
2 "Diagnosed but untreated" ///
3 "Diagnosed and treated" ///
4 "Treated and controlled" ///
5 "Regressed untreated" ///
6 "Regressed uncontrolled"

label values outcome outcome_lbl
tab agecat outcome, missing




******************************
ssc install estout, replace
eststo clear

eststo Undiagnosed: poisson Undiagnosed ///
    i.HIVstatus2 i.agecat i.sex2 i.nationality2 ///
```

```
    if bpcat == 3, vce(robust) irr

eststo DxUntreated: poisson Diagnosedbutuntreated ///
    i.HIVstatus2 i.agecat i.sex2 i.nationality2 ///
    if previouslydiagnosedwithbp == "Yes", vce(robust) irr

eststo DxTreated: poisson Diagnosedandtreated ///
    i.HIVstatus2 i.agecat i.sex2 i.nationality2 ///
    if previouslydiagnosedwithbp == "Yes", vce(robust) irr

eststo TreatedControlled: poisson Treatedandcontrolled2 ///
    i.HIVstatus2 i.agecat i.sex2 i.nationality2 ///
    if baseline_treated == "Yes", vce(robust) irr

eststo RegressedUntreated: poisson Regressed_untreated ///
    i.HIVstatus2 i.agecat i.sex2 i.nationality2 ///
    if baseline_treated == "Yes", vce(robust) irr

eststo RegressedUncontrolled: poisson Regressed_uncontrolled ///
    i.HIVstatus2 i.agecat i.sex2 i.nationality2 ///
    if baseline_controlled == 1, vce(robust) irr

esttab Undiagnosed DxUntreated DxTreated TreatedControlled ///
       RegressedUntreated RegressedUncontrolled, ///
    eform b(2) se(2) ///
    star(* 0.05 ** 0.01 *** 0.001) ///
    label

esttab Undiagnosed DxUntreated DxTreated TreatedControlled ///
       RegressedUntreated RegressedUncontrolled ///
    using "HTN_cascade_models.doc", replace ///
    eform b(2) se(2) ///
    star(* 0.05 ** 0.01 *** 0.001) ///
    label ///
    title("Poisson regression models for hypertension care cascade outcomes")

esttab Undiagnosed DxUntreated DxTreated TreatedControlled ///
       RegressedUntreated RegressedUncontrolled ///
    using "HTN_cascade_models_v2.doc", replace ///
    eform ci ///
    star(* 0.05 ** 0.01 *** 0.001) ///
    label ///
    stats(N, labels("Observations"))

pwd

esttab Undiagnosed DxUntreated DxTreated TreatedControlled ///
       RegressedUntreated RegressedUncontrolled ///
    using "C:\Users\SiphamandlaGumede\OneDrive - Wits Health Consortium\Ezintsha
PC\Desktop\PHD\iHEART SA\SRPs\Spha 2024\HTN_cascade_models.doc", replace ///
    eform ci ///
    star(* 0.05 ** 0.01 *** 0.001) ///
    label ///
    stats(N, labels("Observations"))

esttab Undiagnosed DxUntreated DxTreated TreatedControlled ///
       RegressedUntreated RegressedUncontrolled ///
    using "HTN_cascade_models.xls", replace ///
    eform ci ///
    star(* 0.05 ** 0.01 *** 0.001) ///
    label ///
    stats(N, labels("Observations"))
```

```
esttab Undiagnosed DxUntreated DxTreated TreatedControlled ///
       RegressedUntreated RegressedUncontrolled ///
    using "HTN_cascade_models.doc", replace ///
    eform b(2) se(2) ///
    star(* 0.05 ** 0.01 *** 0.001) ///
    label ///
    stats(N, labels("Observations")) ///
    title("Poisson regression models for hypertension care cascade outcomes")


esttab Undiagnosed DxUntreated DxTreated TreatedControlled ///
       RegressedUntreated RegressedUncontrolled ///
    using "HTN_cascade_models.doc", replace ///
    eform ///
    ci ///
    star(* 0.05 ** 0.01 *** 0.001) ///
    label ///
    stats(N, labels("Observations"))




**poisson Treatedandcontrolled i.HIVstatus2 i.agecat i.sex2 i.nationality2 ///
    if previouslydiagnosedwithbp == "Yes", vce(robust) irr

**poisson Treatedandcontrolled i.HIVstatus2 i.agecat i.sex2 i.nationality2 ///
    if previouslyinitiatedonhtntherapy == "Yes", vce(robust) irr





ta Undiagnosed

* create binary an Diagnosed but untretaed variable=1 (byte to save space)
gen byte Diagnosedbutuntreated=.

replace Diagnosedbutuntreated = 1 if previouslydiagnosedwithbp == "Yes" ///
    & previouslyinitiatedonhtntherapy=="No" ///
    & !missing(previouslydiagnosedwithbp) ///
    & !missing(previouslyinitiatedonhtntherapy)


***Now set Diagnosed but untreated variable = 0 for diagnosed = "Yes"
replace Diagnosedbutuntreated = 0 if previouslydiagnosedwithbp == "Yes" ///
  & previouslyinitiatedonhtntherapy=="Yes" ///
  & !missing(previouslydiagnosedwithbp) ///
  & !missing(previouslyinitiatedonhtntherapy)

ta Diagnosedbutuntreated

* create binary an Diagnosed and treated variable=1 (byte to save space)
gen byte Diagnosedandtreated=.

replace Diagnosedandtreated = 1 if previouslydiagnosedwithbp == "Yes" ///
    & previouslyinitiatedonhtntherapy=="Yes" ///
    & !missing(previouslydiagnosedwithbp) ///
    & !missing(previouslyinitiatedonhtntherapy)


***Now set Diagnosed and treated variable = 0 for diagnosed = "Yes"
replace Diagnosedandtreated = 0 if previouslydiagnosedwithbp == "Yes" ///
```

```
  & previouslyinitiatedonhtntherapy=="No" ///
  & !missing(previouslydiagnosedwithbp) ///
  & !missing(previouslyinitiatedonhtntherapy)

ta Diagnosedandtreated

* create binary Treated and controlled variable=1 (byte to save space)
gen byte Treatedandcontrolled=.

replace Treatedandcontrolled = 1 if previouslyinitiatedonhtntherapy == "Yes" ///
    & systolicbpmeasurementatcurrentvi <140 ///
    & diastolicbpmeasurementatcurrentv <90 ///
    & !missing(previouslyinitiatedonhtntherapy) ///
    & !missing(systolicbpmeasurementatcurrentvi) ///
    & !missing(diastolicbpmeasurementatcurrentv)


***Now set Diagnosed and treated variable = 0 for controlled = "No"
replace Treatedandcontrolled = 0 if previouslyinitiatedonhtntherapy == "Yes" ///
    & systolicbpmeasurementatcurrentvi >= 140 ///
    & diastolicbpmeasurementatcurrentv >= 90 ///
    & !missing(previouslyinitiatedonhtntherapy) ///
    & !missing(systolicbpmeasurementatcurrentvi) ///
    & !missing(diastolicbpmeasurementatcurrentv)

ta Treatedandcontrolled

* create binary Treated and uncontrolled variable=1 (byte to save space)
gen byte Treatedanduncontrolled=.

replace Treatedanduncontrolled = 1 if previouslyinitiatedonhtntherapy == "Yes" ///
    & systolicbpmeasurementatcurrentvi >= 140 ///
    & diastolicbpmeasurementatcurrentv >= 90 ///
    & !missing(previouslyinitiatedonhtntherapy) ///
    & !missing(systolicbpmeasurementatcurrentvi) ///
    & !missing(diastolicbpmeasurementatcurrentv)


***Now set Diagnosed and treated variable = 0 for controlled = "Yes"
replace Treatedanduncontrolled = 0 if previouslyinitiatedonhtntherapy == "Yes" ///
    & systolicbpmeasurementatcurrentvi < 140 ///
    & diastolicbpmeasurementatcurrentv < 90 ///
    & !missing(previouslyinitiatedonhtntherapy) ///
    & !missing(systolicbpmeasurementatcurrentvi) ///
    & !missing(diastolicbpmeasurementatcurrentv)

ta Treatedanduncontrolled

******************Regression model:Transitions**************
* create binary Treated, uncontrolled and regressed to untreated variable=1 (byte to save space)
gen byte Uncontrolledtountreated=.

replace Uncontrolledtountreated = 1 if bloodpressuremedicinesprescribed == "No" ///
    & systolicbpmeasurementatcurrentvi >= 140 ///
    & diastolicbpmeasurementatcurrentv >= 90 ///
    & !missing(bloodpressuremedicinesprescribed) ///
    & !missing(systolicbpmeasurementatcurrentvi) ///
    & !missing(diastolicbpmeasurementatcurrentv)


***Now set Treated, uncontrolled and did not regress
replace Uncontrolledtountreated = 0 if bloodpressuremedicinesprescribed == "Yes" ///
    & systolicbpmeasurementatcurrentvi >= 140 ///
```

```
    & diastolicbpmeasurementatcurrentv >= 90 ///
    & !missing(bloodpressuremedicinesprescribed) ///
    & !missing(systolicbpmeasurementatcurrentvi) ///
    & !missing(diastolicbpmeasurementatcurrentv)

ta Uncontrolledtountreated

* create binary Treated, controlled and regressed to untreated variable=1 (byte to save space)
gen byte Controlledtountreated=.

replace Controlledtountreated = 1 if bloodpressuremedicinesprescribed == "No" ///
    & systolicbpmeasurementatcurrentvi < 140 ///
    & diastolicbpmeasurementatcurrentv < 90 ///
    & !missing(bloodpressuremedicinesprescribed) ///
    & !missing(systolicbpmeasurementatcurrentvi) ///
    & !missing(diastolicbpmeasurementatcurrentv)


***Now set Treated, controlled and did not regress to untreated
replace Controlledtountreated = 0 if bloodpressuremedicinesprescribed == "Yes" ///
    & systolicbpmeasurementatcurrentvi < 140 ///
    & diastolicbpmeasurementatcurrentv < 90 ///
    & !missing(bloodpressuremedicinesprescribed) ///
    & !missing(systolicbpmeasurementatcurrentvi) ///
    & !missing(diastolicbpmeasurementatcurrentv)

ta Controlledtountreated

* create binary Treated, controlled and regressed to uncontrol variable=1 (byte to save space)
gen byte Controlledtouncontrol=.

replace Controlledtouncontrol = 1 if bloodpressuremedicinesprescribed == "Yes" ///
    & systolicbpmeasurementatcurrentvi >= 140 ///
    & diastolicbpmeasurementatcurrentv >= 90 ///
    & !missing(bloodpressuremedicinesprescribed) ///
    & !missing(systolicbpmeasurementatcurrentvi) ///
    & !missing(diastolicbpmeasurementatcurrentv)


***Now set Treated, controlled and did not regress to uncontrol
replace Controlledtouncontrol = 0 if bloodpressuremedicinesprescribed == "Yes" ///
    & systolicbpmeasurementatcurrentvi < 140 ///
    & diastolicbpmeasurementatcurrentv < 90 ///
    & !missing(bloodpressuremedicinesprescribed) ///
    & !missing(systolicbpmeasurementatcurrentvi) ///
    & !missing(diastolicbpmeasurementatcurrentv)

ta Controlledtouncontrol

*****regression model***********
poisson Undiagnosed2 i.HIVstatus2 i.agecat i.sex2 i.nationality2, vce(robust) irr
logit Undiagnosed2 i.HIVstatus2 i.agecat, or
****tabi 2998 3804 \ 6515 4200, chi2
****tabi 2526 3381\2998 3804, chi2
****tabi 1125 2141\2526 3381, chi2

**************************
bys recordid: gen nrows = _N
tab nrows

* how many unique IDs?
egen recordid_tag = tag(recordid)
count if recordid_tag==1
```

```
****************************
gen initial_visit_date = date(dateofvisit, "YMD")
gen followup_date = date(v105, "YMD")
format initial_visit_date followup_date %td

gen followup_days = followup_date - initial_visit_date


count  // before
drop if missing(followup_days) | followup_days <= 0
count  // after

* how many rows have a followup_date and how many have followup_days?
count if !missing(v105)
count if !missing(followup_days)


* (if you don't want to collapse yet, run the two 'egen ...' commands above and then restore original
state)

* show problematic conversions for a sample
list recordid dateofvisit initial_visit_date v105 followup_date if initial_visit_date==. |
followup_date==. in 1/40

* show variable types / formats
describe dateofvisit initial_visit_date v105 followup_date


su followup_days
tabstat followup_days, by(hivstatus) stat(median mean min max)
bys hivstatus: summarize followup_days, detail
ttest followup_days if consentedtoaccessingclinicalinfo=="Yes" & filereviewdone==1, by ( hivstatus )

drop if followup_days < 0
drop if missing(followup_days)

***************************

* 0) Save a copy before you change to participant-level
preserve
save "backup_rowdata.dta", replace

* ---i have: id, initial_visit_date, followup_date, followup_days, hivstatus

* 1) Create participant-level baseline and last-observed dates
bys recordid: egen baseline = min(initial_visit_date)
* Use the latest non-missing followup_date if present, otherwise use the latest visit date
gen tmp_lastdate = followup_date
replace tmp_lastdate = initial_visit_date if missing(tmp_lastdate)
bys recordid: egen lastobs = max(tmp_lastdate)
drop tmp_lastdate

* 2) Compute participant-level follow-up (days)
gen followup_days_pid = lastobs - baseline
format baseline lastobs %td

* 3) Check for negative or missing values and drop them
count if missing(followup_days_pid)
count if followup_days_pid < 0
* drop if missing(followup_days_pid)
```

```
* If none are inconsistent you can take the first (or min/max) value:
* Option A: if hivstatus is stable, take the first recorded value (after sorting by date)
bys recordid (initial_visit_date): gen hiv_pid = hivstatus[1]

* Option B: more robust: take the modal or max value if coded 0/1
* egen hiv_pid = mode(hivstatus), by(id)   // mode is available with egenmore; otherwise:
* bys recordid: egen hiv_min = min(hivstatus)
* bys recordid: egen hiv_max = max(hivstatus)
* if hiv_min==hiv_max then hiv is consistent; else you'll need to inspect those ids
* list recordid if hiv_min!=hiv_max
* gen hiv_pid = hiv_max   // choose convention (e.g., if any record positive → treat as positive)
* drop hiv_min hiv_max n_hivvals

* 5) Keep one record per participant (tag the first occurrence)
egen tag = tag(recordid)
keep if tag==1
drop tag

* Now you have one row per participant with baseline, lastobs, followup_days_pid, hiv_pid

count  // before
drop if missing(followup_days_pid) | followup_days_pid <= 0
count  // after

* 6) Summary statistics (median etc.) by participant-level HIV status
tabstat followup_days_pid, by(hiv_pid) stat(n median mean sd min max)
by hiv_pid, sort: summarize followup_days_pid, detail

tabstat followup_days_pid, by(hiv_pid) stat(median mean min max)
bys hiv_pid: summarize followup_days_pid, detail
ttest followup_days_pid if consentedtoaccessingclinicalinfo=="Yes" & filereviewdone==1, by ( hiv_pid )

tabstat followup_days_pid, by(hiv_pid) stat(median mean min max)
bys hiv_pid: summarize followup_days_pid if consentedtoaccessingclinicalinfo=="Yes", detail
ttest followup_days_pid if consentedtoaccessingclinicalinfo=="Yes", by ( hiv_pid )

ranksum followup_days_pid, by(hiv_pid)
tabstat followup_days_pid, by(hiv_pid) stat(n median p25 p75)


* Optional: convert to months for reporting
gen followup_months_pid = followup_days_pid/30.44
tabstat followup_months_pid, by(hiv_pid) stat(median mean n)

* (Optional) Save the collapsed participant-level dataset
save "participant_level_followup.dta", replace

count  // before
drop if missing(followup_days_pid) | followup_days_pid <= 0
count  // after

* to return to the original row dataset
* restore

***********************

* ensure sorted by id and date
bys recordid (initial_visit_date):

* baseline = first (min) visit date per id
egen baseline2 = min(initial_visit_date), by(recordid)

* lastobs = last (max) follow-up or visit date per id
```

```
egen lastobs2  = max(followup_date), by(recordid)

* if you prefer to use visit_date_s for last observation (if followup_date_s is not present)
* egen lastobs = max(visit_date_s), by(id)

* create follow-up in days, only where both baseline and lastobs exist
gen followup_days2 = lastobs2 - baseline2 if !missing(baseline2, lastobs2)

* drop participants with missing follow-up (optional)
drop if missing(followup_days2)

tabstat followup_days2, by(hivstatus) stat(median mean sd n)



********
preserve
* (if you already have numeric %td variables, skip conversion lines)
* gen initial_visit_date = date(dateofvisit, "YMD")
* gen followup_date       = date(v73, "YMD")
* format initial_visit_date followup_date %td

* baseline = earliest visit
bys recordid: egen baseline3 = min(initial_visit_date)

* lastobs = latest followup_date if present; else latest visit date
gen tmp_lastdate3 = v73
replace tmp_lastdate3 = initial_visit_date if missing(tmp_lastdate)
bys id: egen lastobs3 = max(tmp_lastdate)
drop tmp_lastdate

gen followup_days_pid3 = lastobs3 - baseline3
format baseline lastobs %td

* show how many participants have missing/zero/negative follow-up
bys recordid: keep if _n==1
count
count if missing(followup_days_pid3)
count if followup_days_pid3 == 0
count if followup_days_pid3 < 0

* inspect examples
list id baseline3 lastobs3 followup_days_pid3 if missing(followup_days_pid3) in 1/30
list id baseline3 lastobs3 followup_days_pid3 if followup_days_pid3==0 in 1/30
list id baseline3 lastobs3 followup_days_pid3 if followup_days_pid3<0 in 1/30

* when satisfied, drop unwanted participants
*drop if missing(followup_days_pid3) | followup_days_pid3 <= 0

* compute median by HIV (make sure hiv_pid exists and is participant-level)
* create participant-level HIV status (choose one rule: baseline status, any-positive, last-recorded)
* Example: treat anyone ever positive as positive:
bys recordid: egen hiv_max = max(hivstatus)
tabstat followup_days_pid, by(hiv_max) stat(n median mean sd min max)
```
