# Supplementary material for: The longitudinal care cascade for hypertension in people with and without HIV in South Africa: A clinic-based observational study
Source: PLoS Med. 2026 Sep 11;23(9):e1004957. doi: 10.1371/journal.pmed.1004957 (PMC13567817; doi:10.1371/journal.pmed.1004957)
Supplement: Supplementary file 4 [file pmed.1004957.s006.pdf]

STROBE Statement — checklist of items that should be included in reports of cohort studies

Manuscript: The longitudinal care cascade for hypertension in people with and without HIV in South Africa: a clinic-based observational study (Gumede et al.)

Page and line numbers refer to the manuscript as submitted (continuous line numbering).

Item No	Recommendation	Reported on page / line No.
**Title and abstract**		
**1a**	Indicate the study's design with a commonly used term in the title or the abstract	*Title (p.1, line 1: "longitudinal care cascade"); Abstract Methods and Findings (p.2, lines 54–55: "longitudinal secondary analysis")*
**1b**	Provide in the abstract an informative and balanced summary of what was done and what was found	*Abstract, Background/Methods and Findings/Conclusions (pp.2–3, lines 44–83)*
**Introduction**		
**2**	Background/rationale: Explain the scientific background and rationale for the investigation being reported	*Background (p.5–6, lines 82–109)*
**3**	Objectives: State specific objectives, including any prespecified hypotheses	*Background, closing paragraph (p.6, lines 110–114); prespecified hypothesis restated in Methods (p.6, lines 127–129)*
**Methods**		
**4**	Study design: Present key elements of study design early in the paper	*Methods, Data source (p.6, lines 125–129); STROBE reporting statement (p.7, lines 131–133)*
**5**	Setting: Describe the setting, locations, and relevant dates, including periods of recruitment, exposure, follow-up, and data collection	*Study setting (p.6, lines 117–123); recruitment/follow-up dates (p.7, lines 138–147)*
**6a**	Cohort study: Give the eligibility criteria, and the sources and methods of selection of participants. Describe methods of follow-up	*Parent trial eligibility (p.7, lines 131–132); Sample size, inclusion and exclusion criteria (p.12, lines 236–240); follow-up methods (p.9, lines 182–189)*
**6b**	Cohort study: For matched studies, give matching criteria and number of exposed and unexposed	*Not applicable – participants were not individually matched by exposure (HIV status)*
**7**	Variables: Clearly define all outcomes, exposures, predictors, potential confounders, and effect modifiers. Give diagnostic criteria, if applicable	*Defining longitudinal HTN care cascade, incl. BP/diagnosis/treatment definitions (pp.8–11, lines 156–221; Table 1); Sociodemographic covariates (p.12, lines 241–244)*
**8**	Data sources/measurement: For each variable of interest, give sources of data and details of methods of assessment. Describe comparability of assessment methods if there is more than one group	*BP/diagnosis ascertainment (p.8, lines 157–166); antihypertensive medication ascertainment (p.8, lines 167–178); S1 Appendix and S2 Table (p.43, lines 809–823)*

Item No	Recommendation	Reported on page / line No.
**9**	Bias: Describe any efforts to address potential sources of bias	*Limitations paragraphs on recording/misclassification bias, clustering, and sampling bias (pp.32–35, lines 554–623)*
**10**	Study size: Explain how the study size was arrived at	*All eligible participants with completed medical file review were included (p.12, lines 236–240; p.14, lines 280–282); formal minimum sample size calculation reported in S1 Protocol (p.43, lines 824–828)*
**11**	Quantitative variables: Explain how quantitative variables were handled in the analyses. If applicable, describe which groupings were chosen and why	*Age categorised as 18–39, 40–49, 50–59, 60+ (p.12, lines 242–244); BP categories defined (p.8, lines 164–166)*
**12a**	Statistical methods: Describe all statistical methods, including those used to control for confounding	*Statistical analyses (p.12–13, lines 245–256)*
**12b**	Describe any methods used to examine subgroups and interactions	*Not applicable – no subgroup or interaction analyses were conducted; only prespecified covariate-adjusted models were run (p.13, lines 268–270)*
**12c**	Explain how missing data were addressed	*Complete-case analysis approach (p.13, lines 265–267); Table 2 legend (p.15–16, lines 306–308); Table 3 legend (p.27, line 395)*
**12d**	Cohort study: If applicable, explain how loss to follow-up was addressed	*Variable follow-up and denominators explained in Methods (p.7, lines 138–147; p.9, lines 182–189; p.13, lines 257–260) and discussed as a limitation (p.33, lines 576–590)*
**12e**	Describe any sensitivity analyses	*Not applicable – no sensitivity analyses were conducted; prespecified analyses only (p.13, lines 268–270); potential future sensitivity approaches noted in Limitations (p.32, lines 567–569)*
**Results**		
**13a**	Report numbers of individuals at each stage of study—eg numbers potentially eligible, examined for eligibility, confirmed eligible, included in the study, completing follow-up, and analysed	*Results, opening paragraph (p.14, lines 280–282); S1 Fig flow diagram (p.43, lines 803–808)*
**13b**	Give reasons for non-participation at each stage	*S1 Fig flow diagram and legend (p.43, lines 803–808)*
**13c**	Consider use of a flow diagram	*S1 Fig (referenced p.14, line 282; p.12, line 240; detailed p.43, lines 803–808)*
**14a**	Descriptive data: Give characteristics of study participants (eg demographic, clinical, social) and information on exposures and potential confounders	*Sample characteristics (p.14–16, lines 283–298); Table 2 (p.15)*
**14b**	Indicate number of participants with missing data for each variable of interest	*Table 2 legend (p.15–16, lines 304–308); Table 3 legend (p.27, line 395)*

Item No	Recommendation	Reported on page / line No.
**14c**	Cohort study: Summarise follow-up time (eg, average and total amount)	*Median/mean follow-up duration by HIV status (p.14, lines 288–290; Table 2, p.15)*
**15**	Outcome data: Cohort study—Report numbers of outcome events or summary measures over time	*Movement in the longitudinal HTN care cascade, Progression (p.16–17, lines 313–341; Fig. 2) and Regression (p.17–18, lines 342–362; Fig. 3)*
**16a**	Main results: Give unadjusted and confounder-adjusted estimates and their precision (eg, 95% CI). Make clear which confounders were adjusted for and why they were included	*Regression analysis narrative (p.18–19, lines 363–384); Table 3a unadjusted (pp.20–26) and Table 3b adjusted (pp.20–27) risk ratios; adjustment variables explained (p.27, lines 445–446 and Table 3b legend, p.27, line 1473)*
**16b**	Report category boundaries when continuous variables were categorized	*Age category boundaries (p.12, lines 242–244); BP category boundaries (p.8, lines 164–166); Table 3a/3b column headers (pp.20–27)*
**16c**	If relevant, consider translating estimates of relative risk into absolute risk for a meaningful time period	*Not applicable – results are reported as risk ratios only (Tables 3a–3b, pp.20–27); absolute risk over a fixed period was not estimated given variable follow-up duration (see Limitations, p.33, lines 576–590)*
**17**	Other analyses: Report other analyses done—eg analyses of subgroups and interactions, and sensitivity analyses	*Not applicable – only the prespecified primary analyses were conducted; no additional post hoc or data-driven analyses were undertaken (p.13, lines 268–270)*
**Discussion**		
**18**	Key results: Summarise key results with reference to study objectives	*Discussion, opening paragraph (p.28, lines 461–472)*
**19**	Limitations: Discuss limitations of the study, taking into account sources of potential bias or imprecision. Discuss both direction and magnitude of any potential bias	*Limitations (pp.32–35, lines 554–623)*
**20**	Interpretation: Give a cautious overall interpretation of results considering objectives, limitations, multiplicity of analyses, results from similar studies, and other relevant evidence	*Discussion (pp.29–34, lines 473–617); Conclusion paragraph (p.35, lines 624–633)*
**21**	Generalisability: Discuss the generalisability (external validity) of the study results	*Discussion (p.28, lines 471–472); Limitations, single-setting/clinic-attending population (p.34, lines 603–608)*
**Other information**		
**22**	Funding: Give the source of funding and the role of the funders for the present study and, if applicable, for the original study on which the present article is based	*Funding (p.35–36, lines 642–648)*

Note: An explanation and elaboration article discusses each checklist item and gives methodological background and published examples of transparent reporting. The STROBE checklist is best used in conjunction with this article (freely available on the websites of PLOS Medicine at http://www.plosmedicine.org/, Annals of Internal Medicine at http://www.annals.org/, and Epidemiology at http://www.epidem.com/). Information on the STROBE Initiative is available at http://www.strobe-statement.org.
