# Supplementary material for: The longitudinal care cascade for hypertension in people with and without HIV in South Africa: A clinic-based observational study
Source: PLoS Med. 2026 Sep 11;23(9):e1004957. doi: 10.1371/journal.pmed.1004957 (PMC13567817; doi:10.1371/journal.pmed.1004957)
Supplement: Supplementary file 5 [file pmed.1004957.s008.pdf]

Ezintsha

Date: 31 May 2026

Manuscript Title: The longitudinal care cascade for hypertension: a clinic-based study of people with and without HIV in South Africa

Dear PLOS Medicine editor,

Please find our detailed point-by-point responses to all comments from the Editor and Reviewers.

We have carefully considered all comments and suggestions and have revised the manuscript accordingly. A detailed response to each comment is provided below. Changes made in the revised manuscript have been highlighted using track changes to facilitate review.

The revised manuscript and all responses have been reviewed and approved by all co-authors.

Sincerely,

On behalf of all authors

Dr. Siphamandla Gumede
BSc, MSc (Med), PhD
C: +27 72 362 1793 |**O**: +27 11 084 4930|**E:** sgumede@ezintsha.org |**W:** www.ezintsha.org
32 Princess of Wales Terrace, Building C, Ground Floor, Parktown, 2193, South Africa
Ezintsha is a Division of Wits Health Consortium, University of the Witwatersrand, Johannesburg
Ezintsha

Point by Point responses to editors' and reviewers' comments

Journal Requirements:

1) Your current Financial Disclosure states "This work was supported by the National Heart, Lung, and Blood Institute (NHLBI) of the National Institutes of Health (NIH). Funding was provided through the Small Research Projects grant R01 5U24HL154426, awarded to [SG].NHLBI: https://zaf01.safelinks.protection.outlook.com/?url=https%3A%2F%2Fwww.nhlbi.nih.gov%2F&data=05%7C02%7Csgumede%40ezintsha.org%7Ccd3c7964d6154035a5bd08de9f6ef2eb%7Ca855971766f040d8803d6e989abac34c%7C0%7C0%7C639123496047181978%7CUnknown%7CTWFpbGZsb3d8eyJFbXB0eU1hcGkiOnRydWUsIlYiOiIwLjAuMDAwMCIsIlAiOiJXaW4zMiIsIkFOIjoiTWFpbCIsIldUIjoyfQ%3D%3D%7C0%7C%7C%7C&sdata=rmKaeMtSyjsKdEGY%2F1mD3ZIUQNKWu1WSQvWqsr%2BO4ZE%3D&reserved=0 NIH: https://zaf01.safelinks.protection.outlook.com/?url=https%3A%2F%2Fwww.nih.gov%2F&data=05%7C02%7Csgumede%40ezintsha.org%7Ccd3c7964d6154035a5bd08de9f6ef2eb%7Ca855971766f040d8803d6e989abac34c%7C0%7C0%7C639123496047208778%7CUnknown%7CTWFpbGZsb3d8eyJFbXB0eU1hcGkiOnRydWUsIlYiOiIwLjAuMDAwMCIsIlAiOiJXaW4zMiIsIkFOIjoiTWFpbCIsIldUIjoyfQ%3D%3D%7C0%7C%7C%7C&sdata=KKlZ8Cs4Yi6Uo9ABf7aMjcl1kd84lJePkOWthTkygUU%3D&reserved=0. The funders had no role in the study design; data collection, analysis, or interpretation; the decision to publish; or the preparation of the manuscript. The content is solely the responsibility of the authors and does not necessarily represent the official views of the National Institutes of Health."

However, your funding information on the submission form indicates that Grant Recipient is 'Not applicable' for funder name "National Heart, Lung, and Blood Institute". Please indicate by return email the full and correct funding information for your study and confirm the order in which funding contributions should appear. Please be sure to indicate whether the funders played any role in the study design, data collection and analysis, decision to publish, or preparation of the manuscript.

Response: Thank you for your query regarding the funding information and financial disclosure.

We would like to clarify that this work was supported by the National Heart, Lung, and Blood Institute (NHLBI) of the National Institutes of Health (NIH) under grant number **R01 5U24HL154426**, awarded to **[SG]**. The designation of "Not applicable" for the grant recipient in the submission system was entered in error.

We confirm that the correct funding statement should read as follows:

"This work was supported by the National Heart, Lung, and Blood Institute (NHLBI) of the National Institutes of Health (NIH) under grant number R01 5U24HL154426, awarded to [SG]. The funders had no role in the study design; data collection, analysis, or interpretation; the decision to publish; or the preparation of the manuscript. The content is solely the responsibility of the authors and does not necessarily represent the official views of the National Institutes of Health."

There is a single funding source for this study, and therefore no prioritization of multiple funders is required.

We will ensure that the submission system is updated accordingly to reflect the correct grant recipient information.

Please let us know if any additional clarification is required.

2) Please upload a copy of Figure [1,2,3] which you refer to in your text on page [6,12,13]. Or, if the figure is no longer to be included as part of the submission please remove all reference to it within the text.

Response: Figures 1, 2, 3 (and a new Figure 4) are intended to remain part of the submission and have now been uploaded separately as part of the revised manuscript files. References to these figures within the manuscript text have been retained and checked for consistency.

NOTE - Please provide separate figure files [Figures 1-3] in .tif or .eps format. For more information about figure files please see our guidelines: https://zaf01.safelinks.protection.outlook.com/?url=https%3A%2F%2Fjournals.plos.org%2Fplosmedicine%2Fs%2Ffigures&data=05%7C02%7Csgumede%40ezintsha.org%7Ccd3c7964d6154035a5bd08de9f6ef2eb%7Ca855971766f040d8803d6e989abac34c%7C0%7C0%7C639123496047224663%7CUnknown%7CTWFpbGZsb3d8eyJFbXB0eU1hcGkiOnRydWUsIlYiOiIwLjAuMDAwMCIsIlAiOiJXaW4zMiIsIkFOIjoiTWFpbCIsIldUIjoyfQ%3D%3D%7C0%7C%7C%7C&sdata=sJZbKeAm2hxw0lHp4kXK%2FZspPQK%2ByesBqDyKeU01IFM%3D&reserved=0 , https://zaf01.safelinks.protection.outlook.com/?url=https%3A%2F%2Fjournals.plos.org%2Fplosmedicine%2Fs%2Ffigures%23loc-file-requirements&data=05%7C02%7Csgumede%40ezintsha.org%7Ccd3c7964d6154035a5bd08de9f6ef2eb%7Ca855971766f040d8803d6e989abac34c%7C0%7C0%7C639123496047239243%7CUnknown%7CTWFpbGZsb3d8eyJFbXB0eU1hcGkiOnRydWUsIlYiOiIwLjAuMDAwMCIsIlAiOiJXaW4zMiIsIkFOIjoiTWFpbCIsIldUIjoyfQ%3D%3D%7C0%7C%7C%7C&sdata=HDieQqKCB80rCE%2B%2ByAus9CvyusoFI44wqxs2xgm1aCA%3D&reserved=0

Response: Thank you for this reminder. We have prepared and uploaded figures as separate figure files in the required format and resolution in accordance with the PLOS Medicine figure guidelines. Where applicable, the figures were processed using the PLOS NAAS tool to ensure compliance with journal requirements prior to resubmission.

3)Please ensure that all Figure files have corresponding citations and legends within the manuscript. Currently, [Table 2] in your submission does not have an in-text citation. Kindly include appropriate intext citation.

Response: We reviewed all figures and tables to ensure that corresponding in-text citations and legends are included within the manuscript. An in-text citation for Table 2 (now table 3) has now been added to the Results section.

Revised text:
"The full adjusted regression results are presented in table 3. To facilitate interpretation of patterns across outcomes, fig. 4 provides a heatmap visualization of the adjusted risk ratios and their."

4)We have noticed that you have uploaded Supporting Information files [S1 Fig], but you have not included a list of legends. Please add a full list of legends for your Supporting Information files after the references list.

Response: A supporting information legends section has now been added after the references section of the manuscript. In addition, the participant flow description in the result section now includes an in-text citation to S1 Fig.

Revised text: S1 Fig. Flow diagram illustrating participants with known HIV status who were included in the study from August 2022 to May 2024. Legends: Flow diagram showing participant screening, eligibility assessment, consent, and chart review completion for inclusion in the study. Of 37,620 individuals screened in the iHEART-SA study, 23,855 participants with known HIV status and completed chart review records were included in the analysis. Reasons for exclusion at each stage are shown.

Comments from the reviewers:

Reviewer #1:

Detailed feedback on the manuscript is provided below. My primary focus has been methodological, but I have made a few broader points as well. All items are major unless indicated otherwise.

1. [MINOR] I suggest the authors follow appropriate reporting guidelines for their study (e.g., https://zaf01.safelinks.protection.outlook.com/?url=https%3A%2F%2Fwww.equator-network.org%2Freporting-guidelines%2Fstrobe%2F&data=05%7C02%7Csgumede%40ezintsha.org%7C701c27ba1211402807a008de9ecb12ab%7Ca855971766f040d8803d6e989abac34c%7C0%7C0%7C639122792985928967%7CUnknown%7CTWFpbGZsb3d8eyJFbXB0eU1hcGkiOnRydWUsIlYiOiIwLjAuMDAwMCIsIlAiOiJXaW4zMiIsIkFOIjoiTWFpbCIsIldUIjoyfQ%3D%3D%7C0%7C%7C%7C&sdata=as7Y8eKduGVl88fA5eTFWrjdpf0OMd1ytvqQpxtmcQc%3D&reserved=0) and include the completed checklist as a supplement.

Response: We appreciate the reviewer's suggestion regarding reporting standards. To improve reporting transparency and completeness, the manuscript was reviewed and revised in line with the STROBE reporting guidelines for observational studies. A completed STROBE checklist has been included as part of the supplementary materials accompanying the revised manuscript.

2. The population for this study is patients attending primary care (not the general population), but this important qualification is omitted by the authors at key points in the manuscript e.g., the first sentence of the Discussion. While this can be inferred from the rest of the manuscript I think it's still potentially misleading to suggest the manuscript is presenting the hypertension care cascade for the general population.

Response: We have revised the manuscript to consistently clarify the facility-based nature of the study population. Specifically, we have:

- Revised the opening sentence of the Discussion to explicitly refer to "adults attending primary healthcare clinics."
- Updated relevant sections of the manuscript (Abstract, Methods, and Discussion) to ensure consistent language indicating that the study reflects a clinic-attending population.
- Strengthened the limitations section to explicitly state that the findings may not be generalizable to the broader population, particularly individuals not engaged in routine care.

The revised text in the limitations section now reads:

"The study sample was drawn from a single urban setting in South Africa and was restricted to individuals attending primary healthcare clinics; therefore, the findings may not be generalizable to other settings or to the broader population, particularly individuals not engaged in routine care."

These revisions clarify the scope of inference and reduce the potential for overgeneralisation.

3. [MINOR] I'm not sure if all the details on the original iHEART-SA trial in three paras of the Methods (lines 101-116) are necessary. I found it a little confusing and distracting as I was not sure if the info on the different populations applied directly to the current study or not. I surmise some details are superfluous given that all patients irrespective of HIV status were included per lines 117-118 and Figure S2. I suggest

the authors consolidate these three paras into one, only present what is directly pertinent to the current study, and point any interested readers to other publication(s) for further detail.

Response: We agree that the original description of the parent iHEART-SA trial included more detail than was necessary for the present secondary analysis. To improve clarity and focus, we substantially revised and condensed the introductory Methods section to retain only information directly relevant to the current study, including the study setting, participant population, follow-up period, and data sources.

The revised text also clarifies that the study population reflects individuals actively engaged in care within public primary healthcare clinics rather than the general population, and explains that the predominance of PLHIV reflects routine service utilization patterns within participating clinics rather than sampling intended to achieve balanced representation by HIV status. Additional methodological details regarding the parent iHEART-SA trial are now referenced through the appropriate supporting publication.

4. Was there a minimum follow-up or were all patients included (including those with only one visit)? Suggest authors make this clear in the Methods.

Response: We revised the methods section to clarify that participants were included in the longitudinal analyses if they had baseline data and at least one follow-up clinical record available during the study period.

Revised text: Participants were included in the longitudinal analyses if they had baseline data and at least one follow-up clinical record available during the study period.

5. I found the section "Defining longitudinal HTN care cascade" (line 124) a bit repetitive and confusing. For example, diagnosed with controlled BP is defined twice - once at line 135 and again at line 152. More troublingly, two conflicting definitions of antihypertensive treatment are provided at lines 132 and 149 (discussed in more detail below). Suggest the authors remove repetition and ensure consistency in this section, focusing specifically on the exact definitions used in this research.

Response: We agree that the original "Defining longitudinal HTN care cascade" section contained repetition and inconsistencies, particularly in the definitions of antihypertensive treatment and BP control.

We have substantially revised this section to improve clarity, consistency, and alignment with the analytic approach. Specifically, we have:

- Removed duplicated definitions and consolidated all cascade-related definitions into a single structured section.
- Clearly distinguished between **baseline cascade stage definitions** and **longitudinal outcomes (progression and regression)**.
- Standardised the definition of antihypertensive treatment and clarified how treatment status is assessed at baseline and follow-up.
- Defined BP control once and applied it consistently across all cascade stages and outcomes.
- Retained and clarified denominators for each stage and outcome to ensure transparency and interpretability.

The revised section now presents mutually exclusive baseline categories followed by clearly defined longitudinal outcomes, with consistent terminology and no repetition. These changes ensure that the

cascade definitions are precise, internally consistent, and directly aligned with the analytical framework used in this study.

6. In addition to the previous point, I am concerned about some of the definitions used in the longitudinal hypertension care cascade. There are many medicines that exhibit an antihypertensive effect but not all are routinely used for hypertension. As such, it is important that the authors present the precise search strategy they used to identify hypertension-defining medicines (e.g., ATC codes, active ingredients) either in the body of the manuscript or as a supplement. The illustrative list presented at lines 133-134 lacks precision and is not sufficient.

Response: In the revised manuscript, we have clarified how treatment status was determined and added a supplementary table (S2 Table) listing the medications used to define antihypertensive treatment. Treatment status was based on medications documented in the medical record using predefined medication fields in the chart review tool.

Revised text: "Antihypertensive treatment was defined based on medications documented in the medical record at the relevant timepoint. Medication information was collected using predefined medication fields in the iHEART-SA file review instrument, which captured antihypertensive agents routinely prescribed within South African public sector primary healthcare services during the study period (S1 Appendix).".

We believe these revisions improve the transparency and reproducibility of the analysis.

7. Relatedly to the two points above, in that same sentence (lines 133-134) I am a little confused by what is meant by "… documented in the medical record as taking … at any timepoint." Do the clinicians keep the list of current medicines up to date so it is clear what medicines a patient is on at each visit or was this inferred from prescribing records? I note it is difficult to ascertain whether a patient is on a medicine from prescribing records because single prescriptions can cover long time periods that may include multiple presentations for care. I am also concerned about the conflicting definition at line 152 that suggests patients are only considered treated if they are on drug at baseline. Surely this can change over time if a patient initiates therapy after the baseline visit? I suggest the authors carefully clarify this.

Response: We have clarified that treatment status was assessed at specific timepoints based on documentation in routine medical records, rather than being defined as "ever treated." At baseline, treatment status was determined using medication information recorded at the enrolment visit and used to classify participants into initial cascade stages. During follow-up, treatment status was reassessed at each available timepoint based on documented medication use, allowing for changes in treatment status over time, including initiation and discontinuation.

Revised text: "Antihypertensive treatment was defined based on medications documented in the medical record at the relevant timepoint. Medication information was collected using predefined medication fields in the iHEART-SA file review instrument, which captured antihypertensive agents routinely prescribed within South African public sector primary healthcare services during the study period (S1 Appendix). Treatment status at baseline was determined using medication information recorded at the enrolment visit and used to classify participants into initial cascade stages. During follow-up, treatment status was reassessed at each available visit based on recorded medication use. As detailed prescription duration data were not available, treatment status reflects documented medication use at each visit rather than inferred continuity between visits".

These revisions improve the clarity and internal consistency of the treatment definition and better reflect the longitudinal nature of the analysis.

8. I think it is worth stating explicitly that patients can enter the cohort in any state depending on their BP and antihypertensive prescribing status at their first (baseline) visit.

Response: We have revised the methods section to state clearly that participants were classified into cascade stages at baseline based on their blood pressure measurements, diagnosis status, and treatment documentation, and could therefore enter the study at any stage of the cascade (Inserted in Table 1). This clarification improves transparency regarding the study design and the interpretation of transition outcomes.

9. [MINOR] I think the authors meant "stage" instead of "outcome" at "Estimates were reported for each outcome…" (line 162).

Response: We have revised the wording in the methods section to refer to “cascade stages and outcomes” to accurately distinguish between baseline classification and longitudinal outcomes.

10. The paras under the section "Sample size, inclusion, and exclusion criteria" are results that should be included in the Results section, not the Methods. Instead, the authors should provide any sample size considerations and inclusion/exclusion criteria not already discussed or remove this section from the Methods entirely.

Response: We revised the methods section to retain only the study eligibility and inclusion criteria in the methods section and moved participant screening, enrolment, and inclusion numbers to the results section alongside the study flow description. This restructuring improves the distinction between study design and study findings and aligns the manuscript more closely with reporting standards for observational studies.

11. In the "Statistical analyses" section I am not sure what is meant by "For the tests of associations between HIV status and covariates, the Pearson chi-square test was used in case of dichotomous and categorical variables, and a t-test in case of continuous covariables." Generally, p-values should not be used in descriptive tables (see section 14a of the expanded STROBE guidelines https://zaf01.safelinks.protection.outlook.com/?url=https%3A%2F%2Fjournals.plos.org%2Fplosmedicine%2Farticle%3Fid%3D10.1371%2Fjournal.pmed.0040297&data=05%7C02%7Csgumede%40ezintsha.org%7C701c27ba1211402807a008de9ecb12ab%7Ca855971766f040d8803d6e989abac34c%7C0%7C0%7C639122792985944752%7CUnknown%7CTWFpbGZsb3d8eyJFbXB0eU1hcGkiOnRydWUsIlYiOiIwLjAuMDAwMCIsIlAiOiJXaW4zMiIsIkFOIjoiTWFpbCIsIldUIjoyfQ%3D%3D%7C0%7C%7C%7C&sdata=G3MZ3JGlEKLLk2GDD1uJmi2mHYeAqPS7zm7M6bUOjUM%3D&reserved=0). If the authors are talking about transition probabilities, HIV status is already included in the Poisson models, isn't it? Suggest the authors consider this and adjust methods and results accordingly.

Response: We appreciate this observation and have revised the statistical analyses section accordingly. The original wording implied formal hypothesis testing within descriptive baseline tables, which was not the intention of the analysis.

To improve clarity and align with STROBE recommendations, we removed references to Pearson chi-square and t-tests and now present baseline characteristics using descriptive statistics only. The revised text specifies that frequencies, proportions, medians, and interquartile ranges were used to summarise participant characteristics and transitions across the HTN care cascade.

Inferential analyses examining progression and regression across the cascade are now described exclusively through the Poisson regression models, with adjusted risk ratios and 95% confidence intervals reported.

12. It appears the authors are using Poisson regression to obtain relative risk estimates for their binary outcomes. While this approach can be appropriate, extra care must be taken. There are plenty of resources available on this, but the authors could start with this one https://zaf01.safelinks.protection.outlook.com/?url=https%3A%2F%2Fbmcmedresmethodol.biomedcentral.com%2Farticles%2F10.1186%2Fs12874-018-0519-5&data=05%7C02%7Csgumede%40ezintsha.org%7C701c27ba1211402807a008de9ecb12ab%7Ca855971766f040d8803d6e989abac34c%7C0%7C0%7C639122792985959961%7CUnknown%7CTWFpbGZsb3d8eyJFbXB0eU1hcGkiOnRydWUsIlYiOiIwLjAuMDAwMCIsIlAiOiJXaW4zMiIsIkFOIjoiTWFpbCIsIldUIjoyfQ%3D%3D%7C0%7C%7C%7C&sdata=%2BchP8OpxbhT1x69Oe6vi51oNgqx9hih5yHw2JEOzIfs%3D&reserved=0. Please provide more detail on this including an appropriate citation.

Response: We appreciate this methodological observation and have revised the statistical analyses section to provide additional detail regarding the regression approach used in the study. Specifically, we clarified that modified Poisson regression models with clustered robust standard errors at the clinic level were used to estimate adjusted risk ratios (aRRs) for binary longitudinal HTN care cascade outcomes.

This approach was selected because several outcomes were relatively common, and adjusted risk ratios were considered more interpretable than odds ratios derived from logistic regression models in this context. We also added an appropriate methodological reference supporting this analytical approach *[Zou G. A Modified Poisson Regression Approach to Prospective Studies with Binary Data. Am J Epidemiol. 2004;159(7). doi 10.1093/aje/kwh090]*

13. In terms of the variables included in the statistical models, I am not yet sufficiently clear on exactly what was included and when. The authors say "…with each sociodemographic characteristic as the main exposure." (line 195) but weren't all variables included in an appropriate multivariable model? How was clustering accounted for given nine clinics contributed data? Generally speaking, enough detail should be included in the Methods so an interested reader can replicate the analyses. Including abridged modelling code as a supplement can also help when analyses for multiple endpoints are being undertaken.

Response: We acknowledge the reviewer's observation and agree that additional detail regarding the modelling strategy was needed. The statistical analyses section has been revised to clearly describe the variables included in the multivariable regression models and how the analyses were conducted.

Modified poisson regression models with clustered robust standard errors at the clinic level were used to estimate adjusted risk ratios for progression and regression outcomes across the longitudinal HTN care cascade. Models included HIV status as the primary exposure variable and were adjusted for age, sex, and nationality. To account for potential within-clinic correlation arising from the inclusion of participants across nine primary healthcare clinics, clinic-level clustered standard errors were used.

We also clarified that analyses for each cascade outcome were restricted to participants eligible for the relevant transition stage, improving transparency and reproducibility of the analytical approach.

14. [MINOR] I don't understand why there are extra boxes in Figure 1 below the main sequence for regressions. Aren't these regressing backwards through the four main sequence boxes?

Response: Fig. 1 has been revised to more clearly depict regression as movement back to earlier cascade stages during longitudinal follow-up. The figure legend was also updated to clarify this interpretation.

15. [MINOR] While the approach taken by the authors is arguably valid (notwithstanding my concerns raised in other points), another approach would have been to use Markov chain methods to model state transitions. I suggest the authors consider this as an optional alternative approach. However, this is not necessarily required for this research to be publishable.

Response: We thank the reviewer for this thoughtful suggestion. Markov chain modelling is indeed a well-established approach for analysing state transitions in longitudinal health data, and we agree it represents a valid alternative framework for examining hypertension cascade transitions. However, given that: (1) our primary aim was to describe observed transitions across the cascade rather than to model transition probabilities; (2) The structure of the available follow-up data and variable follow-up intervals were not optimised for a formal Markov chain approach. We therefore retained the current analytical strategy, which we believe appropriately addresses the study objectives. Nevertheless, we have added a sentence to the Discussion acknowledging Markov chain methods as a potential avenue for future longitudinal HTN cascade analyses. We have, however, added a sentence in the "conclusion" acknowledging Markov chain methods as a potential avenue for future research.

Revised text: Future studies integrating more detailed patient- and provider-level data and applying alternative longitudinal state-transition approaches, such as Markov chain modelling, may provide additional insight into movement across HTN care cascade stages over time.

16. The authors could more explicitly consider clinical vs. statistical significance. This is particularly important in analyses of large datasets where statistical significance/narrow CIs that don't cross critical values are seen with trivially small effects e.g., "aRR 1.004; 95% CI 1.001-1.007" at line 270. Suggest the authors more clearly consider the magnitude of the effect and its meaningfulness when reporting and discussing results.

Response: We appreciate this important point regarding the distinction between statistical and clinical significance, particularly in the context of large sample sizes where small effect estimates may achieve statistical significance.

In response, we revised the Results and Discussion sections to place greater emphasis on the magnitude and clinical relevance of associations rather than statistical significance alone. We also removed interpretation of very small effect estimates that were unlikely to be clinically meaningful despite narrow confidence intervals.

These revisions help focus the interpretation on findings with clearer practical and clinical importance

17. I suggest including in Table 1 the mean and median number of visits in addition to the number of days. Indeed, the number of visits is more informative than the follow-up in days because new information on each patient's health status is only acquired when they present for a visit.

Response: We appreciate this helpful suggestion and agree that the number of clinic visits provides important context for interpreting longitudinal transitions across the HTN care cascade. We revised Table 1 to include the proportion of participants who had more than one visit, as well as summary measures for the number of documented clinic visits, in addition to follow-up duration in days.

Revised Table 1 additions:

Table 1: Descriptive characteristics for the study participants

Participant characteristics	All	PLHIV	PNLHIV
**Participants who had their medical records reviewed**	**23,855**	**18,720**	**5,135**
**Median follow-up duration in days (IQR)**	337 (168-532)	336 (168-525)	358 (169-559.5)
**Mean follow-up duration in days (SD)**	369 (1.8)	368 (2.0)	372 (3.7)
**Had ≥1 follow-up visit, n (%)**	15,553 (65.2%)	12,174 (65.0%)	3,379 (65.8%)
**Number of documented clinic visits incl. initial visit, median (IQR)**	2 (1–4)	2 (1–4)	3 (1–5)
**Number of documented clinic visits incl. initial visit, mean (SD)**	2.8 (2.3)	2.7 (2.1)	3.5 (2.7)

18. [MINOR] The BP severity brackets in Table 1's note overlap and do not match the text. Suggest the authors correct the table notes.

Response: We appreciate the reviewer identifying this inconsistency. The BP category thresholds in the Table 1 footnote have been corrected to remove overlapping ranges and aligned with the definitions presented in the Methods section. The revised categories now use consistent and non-overlapping BP thresholds throughout the manuscript.

Revised text: BP classification*: Normal BP: SBP 120-129 mmHg or DBP 80-85 mmHg; High-normal BP: SBP 130-139 mmHg or 86-89 mmHg; High BP/HTN: SBP of ≥140 mmHg or DBP of ≥90 mmHg

19. Also in Table 1, for the number of days follow-up the SD and IQR do not appear to be plausible when considered together. Suggest the authors double check the numbers here.

Response: We thank the reviewer for identifying this inconsistency. Upon review, we confirmed that the standard deviation values for follow-up duration in days were incorrectly reported in Table 1. These values have now been corrected in the revised manuscript.

Table 1: Descriptive characteristics for the study participants

Participant characteristics	All	PLHIV	PNLHIV
**Participants who had their medical records reviewed**	**23,855**	**18,720**	**5,135**
**Median follow-up duration in days (IQR)**	337 (168-532)	336 (168-525)	358 (169-559.5)
**Mean follow-up duration in days (SD)**	376 (231)~~369 (1.8)~~	370 (229)~~368 (2.0)~~	395 (234)~~372 (3.7)~~

20. The information in the Table 2 notes is important and should also be included in the body of the manuscript where these results are reported.

Response: To improve clarity, we added explanatory text to the Results section immediately preceding Table 2 (now table 3), clarifying that denominators differed across models because analyses were restricted to participants eligible for each cascade transition stage and to observations with complete covariate data.

21. In the Discussion the following assertion was made but I don't think the authors included a statistical comparison of follow up between the two groups "… there was no statistically significant difference in follow-up duration between PLHIV and PNLHIV…"?

Response: We agree that the original wording implied a formal statistical comparison that had not been performed. We therefore revised the Discussion section to use more cautious descriptive language regarding follow-up duration between PLHIV and PNLHIV.

Revised text: Follow-up duration was generally comparable between PLHIV and PNLHIV, indicating that the observed differences in HTN care cascade outcomes are unlikely to be attributable to differential observation time

Reviewer #2:

1. The introduction notes that PLHIV may have higher rates of HTN diagnosis and treatment due to frequent clinical contact (lines 62-66), yet the paper's central finding is the opposite. While this contradiction is handled in the discussion, it may be worth more explicitly highlighting this contrast in the introduction. The framing that disparities may widen for non-PWH compared to PWH sounds slightly odd, so should be rephrased.

Response: We agree that the original framing in the Introduction may have implied an expected advantage in hypertension care outcomes among people living with HIV, which is not necessarily supported by the existing evidence and may not reflect the complexity of hypertension management within routine HIV care. To address this, we revised the Introduction to avoid implying a predetermined direction of effect and instead emphasize the uncertainty surrounding the relationship between HIV status and longitudinal hypertension care outcomes. The revised text acknowledges that engagement with HIV services may create opportunities for hypertension screening and management, while noting that this may not necessarily translate into improved progression and retention across stages of the hypertension care cascade.

Revised text:
““The relationship between HIV status and longitudinal hypertension care outcomes remains unclear. While engagement with HIV services may create opportunities for hypertension screening and management, this does not necessarily translate into improved progression and retention across stages of the hypertension care cascade.”

2. The enrollment criteria between PWH and non-PWH is a methodological concern. The parent trial's effectiveness population were enrolled precisely because they had elevated BP. Because non-PWH were enrolled without a BP eligibility requirement, it means the denominators and the underlying populations being compared are different. Authors should more clearly explain how this affects the comparative findings and comparisons between these two groups remain valid despite different enrollment pathways.

Response: We thank the reviewer for this important methodological observation and agree that the enrolment pathways and derivation of the analytic population required clearer explanation.

We would like to clarify that recruitment into the parent iHEART-SA study was not based on elevated blood pressure status. Adults attending participating public primary healthcare clinics were enrolled irrespective of BP level, and participants were subsequently classified across stages of the HTN care cascade using BP measurements, diagnosis status, and treatment information obtained through routine medical record

review. Comparisons between PLHIV and PNLHIV were therefore conducted within the same underlying clinic-attending population using the same longitudinal follow-up framework and outcome definitions.

To address this concern, we revised the methods section to clarify how the analytic cohort for this secondary analysis was derived and strengthened the limitations section to further contextualize the clinic-based nature of the study population and the interpretation of comparative findings.

Revised text (Methods):
"The parent study enrolled adults aged ≥18 years attending participating clinics, irrespective of HIV status or BP level. In this secondary analysis, participants were subsequently classified across stages of the HTN care cascade using BP measurements, diagnosis status, and treatment information obtained through routine medical record review."

Revised text (Limitations):
"The study sample was drawn from a single urban setting in South Africa and was restricted to individuals attending primary healthcare clinics; therefore, the findings may not be generalizable to other settings or to the broader population, particularly individuals not engaged in routine care."

3. Authors should discuss explicitly whether non-PWH in this sample are representative of the broader non-PWH population with hypertension or whether they represent a selected subgroup (e.g., those already engaging with the health system for other chronic conditions).

Response: We agree that the PNLHIV group included in this clinic-based cohort may not be representative of the broader population of PNLHIV with hypertension.

To address this concern, we revised the discussion section to more explicitly acknowledge that PNLHIV in the study population likely represent a selected subgroup already engaged in healthcare services for chronic or other medical conditions. We further note that this may partially explain the higher observed prevalence of HTN and cardiometabolic risk factors among PNLHIV within this cohort.

Revised text:
"Consequently, PNLHIV in this cohort may represent a selected subgroup already engaged in healthcare services for chronic or other medical conditions, rather than the broader population of PNLHIV in the community. This may partially explain the higher observed prevalence of HTN and cardiometabolic risk factors within this group."

4. While the paper acknowledges the wide variation in follow-up time (12-36 months) and no statistically significant differences are noted, it is also true that participants with longer follow-up had more opportunity to both progress and regress. This muddies the implications for rates of progression and regression. A sensitivity analysis is warranted, or this effect should be included in the limitations.

Response: Although follow-up duration was generally comparable between PLHIV and PNLHIV, we acknowledge that participants with longer observation periods had greater opportunity to transition between cascade stages. Rather than undertake additional time-to-event modelling, which was beyond the scope of the present descriptive longitudinal analysis, we strengthened the limitations section to more explicitly acknowledge the implications of variable follow-up duration for interpretation of progression and regression outcomes.

Revised text:
"Participants with longer follow-up had greater opportunity to experience both progression and regression

across stages of the HTN care cascade, which may have influenced the observed frequency of transitions. Consequently, progression and regression outcomes should be interpreted as reflecting observed longitudinal movement during available follow-up rather than standardized transition rates over fixed time intervals.”

5. I appears that the parent trial was actively implementing the HTN integration intervention across the study period. This could bias the estimates in either direction. Authors should account for this in the secondary analysis or explain this in the limitations section.

Response: We agree that the stepped-wedge implementation design of the parent iHEART-SA trial may have influenced observed progression and regression across the HTN care cascade over time.

The present study was designed as a secondary longitudinal analysis focused on describing movement across HTN care cascade stages rather than evaluating intervention effectiveness. Accordingly, intervention exposure and study period were not explicitly modelled in the present analyses. However, we acknowledge that implementation activities associated with the iHEART-SA intervention may have affected HTN diagnosis, treatment, follow-up, and BP control during the study period.

To address this concern, we strengthened the limitations section to explicitly acknowledge the potential influence of intervention rollout and evolving clinic practices on observed cascade transitions.

Revised text:
“In addition, this secondary analysis was conducted within the broader iHEART-SA stepped-wedge implementation trial, during which clinics transitioned from usual care to an integrated HTN/HIV intervention at different timepoints. As the present analysis did not explicitly model intervention exposure or study period, it is possible that implementation activities influenced observed progression and regression across the HTN care cascade. Consequently, observed longitudinal transitions may partly reflect the effects of ongoing intervention rollout and changing clinic practices over time.”

6. Lines 124-141 define HTN and the cascade categories narratively and lines 142-160 appears to re-state the same information in numbered list format. The redundancy should be resolved. Additionally, the definitions of progression and regression (currently at lines 166-175) should be moved before the numbered cascade stage definitions for logical flow.

Response: The reviewer raises an important point regarding redundancy and flow within the methods section. We revised this section by consolidating overlapping descriptions of HTN care cascade stages and repositioning the definitions of progression and regression earlier in the section to improve readability and conceptual clarity.

7. Consider reformatting the six cascade stage definitions as a table for clarity. The current presentation is difficult to follow because each definition includes denominator information embedded in the text. A table with columns for Stage, Definition, and Denominator would improve readability. Additionally, Figure 1 should also be repositioned to appear adjacent to these definitions rather than at the end of the methods section, where it is currently placed.

Response: We reformatted the cascade stage definitions into a table summarizing the stage, operational definition, and corresponding denominator for each transition outcome. This restructuring improves clarity and reduces the amount of explanatory text embedded within the methods section.

We also repositioned Figure 1 to appear adjacent to the cascade stage definitions to improve the conceptual flow between the textual definitions and the visual representation of progression and regression across the HTN care cascade.

8. The single BP reading issue requires clearer explanation. Single readings are subject to white-coat effect and measurement error, and could meaningfully inflate HTN prevalence and subsequent findings. Lines 125-131 acknowledge that HTN diagnosis ideally requires two consecutive elevated readings, but in this trial only a single reading per visit was available. At which specific timepoint was the single reading used for baseline HTN classification? How were multiple readings across visits handled?

Response: In the present study, baseline HTN classification was determined using BP measurements, diagnosis status, and treatment documentation recorded at the enrolment visit. Although HTN diagnosis ideally requires repeated elevated BP measurements, routine clinical records generally contained only a single BP value per visit, and information regarding repeat confirmatory measurements at the same visit was not consistently available.

During follow-up, BP measurements and treatment status were reassessed at each available clinical visit using routinely documented data. Thus, longitudinal progression and regression across the HTN care cascade were based on sequential BP measurements obtained across multiple visits during routine care rather than repeated measurements within a single visit.

We revised the Methods section to clarify these procedures and further acknowledge the potential for measurement variability and white-coat effects as study limitations.

9. The choice of Poisson regression for binary outcomes rather than logistic regression should be briefly justified.

Response: We added a brief justification for the use of modified Poisson regression models in the statistical analysis section. This approach was selected because several study outcomes were common, and adjusted risk ratios were considered more interpretable than odds ratios for longitudinal HTN care cascade outcomes. We also clarified that cluster-robust standard errors were used to account for clustering at the clinic level.

Revised text:
"Modified Poisson regression models with clinic-clustered robust standard errors were used to estimate adjusted risk ratios (aRRs) for binary progression and regression outcomes across the longitudinal HTN care cascade. This approach was selected because several outcomes were common, and risk ratios were considered more interpretable than odds ratios for the study objectives."

10. ART adherence and retention in HIV care are strong proxies for engagement with the health system, yet they are not included as covariates. The authors should either include these covariates or explain in the limitations why they were unavailable and what bias their absence may introduce.

Response: We acknowledge that ART adherence and retention in HIV care are important proxies for healthcare engagement and may influence longitudinal HTN care cascade outcomes. However, these measures were not systematically available within the secondary routine data used for this study. We therefore added a limitation acknowledging that residual confounding related to differential healthcare

engagement may have influenced the observed associations between HIV status and HTN care cascade transitions.

Revised text:
"Measures of ART adherence and retention in HIV care were not consistently available within the routine clinical records used for this secondary analysis and therefore could not be included as covariates in the regression models. These factors may represent important markers of healthcare engagement among PLHIV and could influence opportunities for HTN screening, treatment, follow-up, and BP control."

11. Missing data handling should be described. The denominators in Table 2 footnotes differ from those in the main results (e.g., age and undiagnosed: n=3,811 vs. the outcome total N=3,950), but the approach to missing values is not explained. Was complete case analysis used? Was missingness related to key variables?

Response: We agree that the handling of missing data required clearer description. Regression analyses were conducted using complete-case data, and participants with missing values for variables included in a given model were excluded from that specific analysis. As a result, denominators varied slightly across outcomes and regression models, which explains the differences observed between overall outcome totals and model-specific sample sizes reported in Table 2.

To improve transparency, we added a clarification to the statistical analysis section describing the complete-case approach and the reason for varying denominators across analyses.

Revised text:
"Analyses were conducted using complete-case data for variables included in each model. Consequently, denominators varied slightly across regression analyses because participants with missing data for specific covariates or outcome classifications were excluded from the relevant model."

12. Table 2 is large and difficult to navigate. Consider moving it to supplementary material and presenting a simplified summary table or a visualization such as a forest plot or annotated heatmap that allows quick identification of meaningful associations.

Response: We thank the reviewer for this helpful suggestion. We agree that the regression results are extensive and that visual presentation can facilitate interpretation of key findings. Rather than moving Table 2 to the supplementary material, we elected to retain it in the main manuscript because it contains the primary adjusted regression results underlying the study's conclusions. However, to improve readability and allow rapid identification of meaningful associations, we developed and included a companion visual summary (Fig. 4) presenting adjusted risk ratios and confidence intervals across outcomes using a heatmap format. We believe that the combination of the detailed regression table and the visual summary provides both transparency and accessibility while preserving the complete reporting of the primary analyses.

13. Figure 2 needs significant revision. 1) The complementary bars for "undiagnosed" and "diagnosed" (which sum to 100%) are both shown which is visually redundant. The figure should be simplified. 2) The second bar is labeled "participants diagnosed with BP" which should read "participants diagnosed with HTN." "Diagnosed with BP" is not clinically meaningful. 3) Since denominators for PWH and non-PWH differ substantially, percentages alone can be misleading, so absolute counts (or denominators) should be added.

Response: We have revised Figures 2 and 3 accordingly.

1. We simplified the cascade figures by presenting only the primary cascade stages of interest and removing complementary categories that are mathematically redundant. This improves readability and reduces visual clutter.
2. The labeling has been corrected throughout the figures and legends. Specifically, the incorrect label "participants diagnosed with BP" has been replaced with "participants diagnosed with HTN."
3. To improve interpretability given the differing denominators between PLHIV and PNLHIV, we added participant counts and denominators (n/N) above each bar and clarified the relevant denominators in the figure legends. This allows readers to interpret both the relative percentages and the underlying sample sizes at each cascade stage.

We believe these revisions have substantially improved the clarity and interpretability of the figures.

14. It is plausiable that vertical HIV programs explain poorer PWH outcomes but it is unclear why it is presented as the only narrative. Authors may consider exploring other angles, such as drug interactions between ART and anti-HTN meds affecting BP control, pill burden leading patients to deprioritize HTN treatment, providers not routinely checking BP during routine HIV visits.

Response: We revised the discussion to incorporate a broader range of plausible mechanisms that may contribute to these findings.

Specifically, we now discuss additional factors including medication burden associated with lifelong ART use, potential prioritization of HIV treatment over HTN management, possible pharmacologic interactions between ART and antihypertensive medications, and inconsistent BP monitoring during routine HIV-focused clinical encounters.

Revised text: Several factors may contribute to the poorer longitudinal HTN care cascade outcomes observed among PLHIV. Although differentiated and vertically structured HIV programs may inadvertently limit integration of HTN management within routine HIV care [22,23], additional mechanisms are also plausible. PLHIV often experience higher medication burden due to lifelong ART use, which may affect adherence to antihypertensive therapy or lead to prioritization of HIV treatment over HTN management [25,26]. Potential pharmacologic interactions between ART regimens and antihypertensive medications may also complicate BP control [26]. In addition, BP monitoring and HTN management may not be consistently prioritized during routine HIV-focused clinical encounters, particularly in resource-constrained settings where consultation time and staffing are limited [27]. These factors likely interact in complex ways and may collectively contribute to observed differences in progression and regression across the HTN care cascade.

15. The Zimbabwean nationality finding (lower regression risk, aRR 0.72) is intriguing and has potential policy implications. It warrants more substantive discussion, particularly in the context of South Africa's large migrant population.

Response: we expanded the discussion section to further contextualize this finding within the literature on migrant health and chronic disease care in Southern Africa. The revised discussion now considers how continuity of care, healthcare engagement, mobility, documentation challenges, and experiences of social exclusion or discrimination within healthcare settings may influence longitudinal HTN care cascade outcomes among migrant populations. At the same time, we emphasize that these findings should be interpreted cautiously, as the present study was not specifically designed to evaluate migrant health outcomes or the structural determinants influencing healthcare access among non-national populations.

Revised text:

These findings should also be interpreted within the broader context of migration and healthcare access in South Africa. Migrant populations may experience complex and sometimes competing influences on healthcare engagement, including barriers related to mobility, continuity of care, documentation challenges, and experiences of social exclusion or discrimination within healthcare settings. At the same time, migrant individuals successfully linked to chronic care services may represent a relatively stable subgroup with sustained healthcare utilization once engaged in care. Differences in healthcare-seeking behaviours and patterns of clinic engagement may therefore contribute to the observed association. However, the present study was not specifically designed to evaluate migrant health outcomes or the broader structural determinants shaping healthcare access among migrant populations."

A thorough proofread is recommended. Examples of minor issues are:

1. Reference formatting at line 58-59 appears to cite "[3,3-7]" — reference 3 appears to be listed twice.

Response: We thank the reviewer for identifying this referencing error. The duplicate citation was unintended and has been corrected in the revised manuscript.

2. Some phrasing implies that HIV status is driving cascade differences. Given the observational nature of the study and the uncontrolled confounding, the language should consistently reflect association rather than causation.

Response: we carefully reviewed the manuscript and revised wording throughout to avoid causal language where inappropriate. Terms implying causation were replaced with language reflecting association, including revisions such as "associated with," "linked to," or "may reflect," where applicable.

Examples of revised wording include:

- "HIV status was associated with progression and regression across stages of the HTN care cascade…"
- "These findings may partly reflect differences in healthcare engagement and service integration…"

These revisions were made throughout the Abstract, Results, and Discussion sections to ensure consistency with the observational study design.

3. The methods section should specify the name of the institutional review board that provided ethical approval along with the reference number.

Response: We revised the methods section to explicitly state the name of the institutional ethics committee and the corresponding approval numbers for both the parent iHEART-SA trial and the present secondary analysis. The Ethical Approval section was also updated for clarity.

Revised text:
"The parent iHEART-SA trial received ethical approval from the Human Research Ethics Committee (Medical) of the University of the Witwatersrand (M211160), and the present secondary analysis was approved by the same committee (M2411128)."

4. Figure 2 labeling inconsistencies in the bar labels. Some use "elevated BP," others use "high BP." This should be standardized.

Response: We identified and corrected inconsistencies in the terminology used within Fig. 2. The labels have now been standardized throughout the figure to use consistent BP classification terminology. Specifically, “elevated BP” and “high BP” labels were harmonized to ensure consistency with the definitions used in the methods section and across the manuscript.

5. Figure 3 would benefit from confidence intervals or p-values being added into the figure rather than only available in Table 2.

Response: We appreciate this helpful suggestion. Figure 3 was designed to provide a visual summary of the direction and magnitude of associations across HTN care cascade outcomes, while detailed numerical estimates, confidence intervals, and p-values are presented in Table 2. We considered adding additional statistical annotations directly to the figure; however, given the number of outcomes and comparisons presented, this substantially reduced figure readability. We therefore retained the current figure format and ensured that all corresponding confidence intervals and p-values remain clearly available in Table 2.

Reviewer #3:

Overall, the manuscript is of good quality. However, I recommend a careful revision of the language and formatting, as some inaccuracies remain. In addition, the quality of Figures 2 and 3 should be improved, and the issues with the axis labels should be corrected.

Response: We thank the reviewer for the positive assessment of the manuscript and for these helpful suggestions. We have carefully reviewed the manuscript for language, formatting, and presentation consistency and corrected typographical, grammatical, and formatting issues identified during the revision process. We have also revised Figures 2 and 3 to improve clarity and visual quality, including correction of axis labels, simplification of the figure presentation, and addition of participant counts and denominators to facilitate interpretation. We believe these revisions have improved the overall readability and presentation of the manuscript.

Reviewer #4:

1. The paper addresses an important question, but the analytic population is still not explained clearly enough. The study uses secondary data from the iHEART-SA trial, which was conducted across nine public clinics and had a stepped-wedge cluster design. That gives this analysis real value. However, I found it difficult at times to follow exactly who contributed to each stage of the cascade and each regression model.

The manuscript would benefit from one short, very clear paragraph in the Methods that explains, in plain language:

* What counted as baseline,
* What counted as follow-up,
* Who was included in each denominator,
* and whether the same participant could contribute to more than one transition outcome.

Some of this information is present, but it is spread across several sections and remains harder to follow than it should be for a paper of this importance.

Response: We thank the reviewer for this helpful suggestion. We agree that, although the relevant methodological details were included in the manuscript, the longitudinal analytic framework could be presented more clearly and cohesively.

In response, we added a concise clarifying paragraph in the Methods section to summarize:
(1) the definition of baseline and follow-up,
(2) how participants contributed to specific progression and regression outcomes across the HTN care cascade,
(3) how denominators were determined for each transition analysis, and
(4) the possibility for participants to contribute to more than one longitudinal transition outcome during follow-up.

This revision was intended to improve readability and provide a clearer overview of the longitudinal analytic population and cascade transition framework.

Revised text:

"For this longitudinal analysis, baseline was defined as the participant's enrolment visit into the iHEART-SA study, at which BP measurements, HTN diagnosis status, and antihypertensive treatment information were recorded from routine clinical records. Follow-up consisted of all subsequent documented clinic visits available through medical record review until May 2025. Participants contributed to specific progression and regression analyses according to their baseline HTN care cascade stage, and denominators therefore differed across transition outcomes. Because participants could experience multiple changes in HTN care status over time, an individual could contribute to more than one longitudinal transition outcome during follow-up."

2. Hypertension definition needs to be presented more honestly and consistently This was one of the main areas where I felt the paper needed more clarity. The manuscript says that hypertension at baseline was defined using two consecutive high BP measurements, or a documented diagnosis, or treatment history. But it then states that, in practice, only a single BP reading per visit was available in the medical record, and there was no information on whether a confirmatory measurement had been taken. That means the paper should state more directly what definition was actually used in the analysis. At the moment, the wording gives the impression that a more rigorous BP definition was applied than the available data could support. Related to this, the BP thresholds are not presented consistently. In the Methods, high BP/HTN is defined as SBP ≥140 mmHg or DBP ≥90 mmHg, but in Table 1 footnote, it is written as SBP >140 mmHg or DBP >90 mmHg. (e.g., lines 129-131), vs. Table 1 footnote). The boundaries for normal and high-normal BP also differ slightly between sections. These definitions need to match throughout the paper.

Respond: We revised the methods section to more explicitly distinguish between the ideal clinical definition of HTN and the measurements that were actually available within the routine medical record data used for this analysis. The revised text now clarifies that HTN classification was operationally based on single documented BP measurements per visit in combination with documented HTN diagnosis and antihypertensive treatment information, as repeat confirmatory BP measurements were not consistently available in the medical records.

We also carefully reviewed and standardized BP threshold definitions throughout the manuscript, including the Methods section, Table 1 footnotes, and related figure labels. Definitions for normal BP, high-normal BP, and high BP/HTN are now presented consistently throughout, and all threshold notation has been harmonized using "≥140 mmHg" and "≥90 mmHg" rather than ">140 mmHg" or ">90 mmHg."

Revised text: HTN was defined using routinely documented BP measurements, documented HTN diagnosis, or antihypertensive treatment recorded in the medical record at the relevant timepoint. In practice, routine medical records generally contained only a single BP reading per visit, and information on whether repeat confirmatory measurements were obtained was not consistently available. Consequently, HTN classification in this analysis was based on single documented BP measurements in combination with diagnosis and treatment documentation recorded during routine care. Although clinical guidelines generally recommend confirmation using repeated BP measurements, this was not possible within the available routine data.

3. Unequal follow-up time needs more careful handling The paper is framed as a longitudinal analysis, but follow-up time varied considerably across participants. Some people contributed close to three years of observation, while others contributed only about twelve months.

This is important because the longer someone is followed, the more opportunity they have both to progress and to regress across the cascade. The manuscript does acknowledge this in the limitations, but I do not think that is enough on its own. The authors should explain more clearly why the analytic approach is still appropriate despite unequal follow-up time, or consider incorporating follow-up duration more directly into the analysis. The statement that follow-up duration did not differ significantly between PLHIV and PNLHIV is helpful, but it does not fully resolve the issue. Unequal follow-up can still affect the probability of observing transition events overall.

Response: We agree that unequal observation time could influence the likelihood of observing progression and regression events across the HTN care cascade.

The primary objective of this analysis was to characterize observed longitudinal movement across HTN care cascade stages during available follow-up rather than estimate standardized transition rates over fixed time intervals. Consequently, the analytic approach focused on whether participants experienced progression or regression during follow-up irrespective of exact observation duration. We have clarified this rationale in the Statistical Analysis section.

We also expanded the limitations section to more explicitly acknowledge that participants with longer follow-up had greater opportunity to experience both progression and regression events, which may have influenced the observed frequency of transitions.

Revised text: "Because the primary objective was to characterize observed progression and regression across the HTN care cascade during available follow-up rather than estimate standardized transition rates over fixed time intervals, analyses focused on whether transitions occurred during follow-up irrespective of exact observation duration."

4. The issue of clustering by clinic should be addressed explicitly Because this analysis comes from a parent study conducted across nine clinics in a cluster stepped-wedge design, it is important to know whether the regression models accounted for clustering at the clinic level. The statistical methods section says that Poisson regression was used and adjusted for age, sex, and nationality, but it does not say whether clinic-

level clustering or correlation was taken into account. That is important because participants from the same clinic are unlikely to be independent. If clustering was not accounted for, the confidence intervals may be too narrow. At a minimum, the manuscript should state clearly what was done here.

Response: We thank the reviewer for highlighting the importance of accounting for within-clinic correlation given the clustered stepped-wedge design of the parent iHEART-SA trial.

In the revised manuscript, we clarified that regression analyses accounted for clustering at the clinic level through the use of robust standard errors clustered by clinic. This approach was used to account for potential non-independence of participants within clinics and to provide appropriately estimated confidence intervals.

Revised text:

Modified Poisson regression models with clustered robust standard errors at the clinic level were used to estimate adjusted risk ratios (aRRs) for binary progression and regression outcomes across the longitudinal HTN care cascade.

5. Table 2 still needs a careful check

T Table 2 still needs another careful review for presentation accuracy and consistency.

For example, for age 18-39 years under the "undiagnosed" outcome, the confidence interval is shown as 1.28-139, which is clearly a typographical or formatting error. In the narrative Results section, the same estimate appears as 1.28-1.39, which is much more plausible.This may look minor, but tables like this need to be exact. More broadly, I encourage the authors to recheck all point estimates, confidence intervals, denominators, and footnotes in Table 2. Some of the adjusted risk ratios are extremely close to 1.00, and while that may be statistically correct, the paper should help the reader understand whether these findings are clinically meaningful.

Response: The confidence interval for participants aged 18–39 years under the “undiagnosed HTN” outcome incorrectly appeared as “1.28–139” due to a typographical error and has been corrected to “1.22,1.46” (values changed after revisions) to align with the results narrative.

In addition, we conducted a comprehensive review of Table 2 to verify the accuracy and consistency of all adjusted risk ratios, confidence intervals, denominators, and footnotes. Minor formatting inconsistencies were corrected throughout the table.

We also agree that some statistically significant associations were modest in magnitude and should be interpreted cautiously with respect to clinical significance. To address this, we revised the Discussion to more carefully contextualize findings with adjusted risk ratios close to 1.00 and avoid overinterpretation of small effect sizes.

6. Some outcome definitions are still difficult to interpret The idea of looking at both progression and regression across the cascade is a strength of the paper. However, the way some outcomes are defined still makes the analysis difficult to interpret. For example, "treated and controlled" is presented as a progression outcome, while "regressed to untreated" and "regressed to uncontrolled" are treated as regression outcomes based on later status. The manuscript says that participants could experience both progression and regression during follow-up. That may reflect real clinical care, but it also means the reader needs a clearer explanation of how people were counted. It would help to state plainly:

*	whether control at any follow-up visit counted,

*	whether the last observed visit was used,

*	and whether the same person could contribute to both a progression and a regression outcome.

Without that clarification, the interpretation of the results remains more difficult than it needs to be.

Response: we revised the Methods section to more explicitly clarify how transition outcomes were defined and counted during follow-up. The revised text now specifies that progression and regression outcomes were based on any observed change in HTN care cascade status during available follow-up visits rather than status at the final recorded visit. We also clarified that participants could contribute to both progression and regression outcomes over time if multiple changes across cascade stages were observed during follow-up.

Revised text:

"Progression and regression across the HTN care cascade were assessed longitudinally during follow-up. These outcomes were defined based on any observed change in HTN care cascade status during available follow-up visits rather than status at the final recorded visit. Participants were classified as having experienced a progression or regression outcome if the relevant transition was documented at any point during routine follow-up care. Consequently, participants could contribute to both progression and regression outcomes over time if multiple changes across cascade stages were observed during follow-up."

We believe these revisions improve the clarity and interpretability of the longitudinal cascade framework and provide a clearer explanation of how participants contributed to transition outcomes during follow-up.

7. Some results outcomes interpretation is certain than the data allow The central message of the paper is important and clearly worth sharing: PLHIV were more likely to remain undiagnosed, less likely to achieve BP control once treated, and more likely to regress to untreated or uncontrolled hypertension.

Where I would encourage more caution is in the discussion of why this may be happening. The manuscript mentions competing clinical priorities, therapeutic inertia, pill burden, and prioritisation of ART adherence. These are all plausible explanations, and many readers will find them believable. But they were not directly measured in this study. I suggest rephrasing these sections so they read as possible explanations rather than implied findings from the analysis itself.

Response: We carefully revised the Discussion section to ensure that mechanisms such as competing clinical priorities, therapeutic inertia, medication burden, and prioritisation of ART adherence are framed as plausible or potential explanations rather than direct findings from the analysis itself. We also reviewed the manuscript more broadly to avoid overly causal or definitive language where unsupported by the available data.

Examples of revised wording include:

- "Several factors may potentially contribute to the poorer longitudinal HTN care cascade outcomes observed among PLHIV…"
- "PLHIV may experience higher medication burden due to lifelong ART use, which could affect adherence to antihypertensive therapy…"

These revisions were made to ensure that interpretation of the findings remains appropriately cautious and aligned with the observational nature of the study.

8. Discussion is relevant, but it could be more focused The discussion is thoughtful and engages with the broader literature on integrated HIV/NCD care, which is valuable. At the same time, some sections could be tighter. In particular, the discussion comparing integrated, vertical, and semi-vertical service models is interesting, but it goes somewhat beyond what this dataset can directly answer. I think the paper would be stronger if the discussion stayed a little closer to three things:

* What this study clearly shows,
* what it cannot explain,
* and what the most reasonable service-delivery implications are.

That would make the message sharper and more persuasive.

Response: we revised and streamlined the Discussion section to maintain a clearer emphasis on:
(1) the key longitudinal HTN care cascade findings observed in this study,
(2) the limitations of the analysis in explaining the mechanisms underlying these differences, and
(3) the most relevant implications for HTN management within routine HIV care services.

We also revised the section discussing integrated, vertical, and differentiated service-delivery approaches to ensure that these are presented as contextual implications informed by existing literature rather than conclusions directly derived from the present analysis. In particular, we reduced broader conceptual discussion regarding optimal service-delivery models and more clearly acknowledged that the study was not designed to directly compare these approaches.

Revised text:

"These findings have important implications for chronic disease care in high HIV-burden settings. PLHIV in this study experienced lower HTN diagnosis, poorer BP control, and higher regression across the HTN care cascade, suggesting persistent gaps in longitudinal HTN management within routine HIV care services. Prior studies suggest that integrating NCD services into HIV care may improve screening, follow-up, and continuity of chronic disease management [19,41–43]. In addition, emerging evidence from community- and home-based HTN care models in South Africa demonstrates the potential of differentiated and patient-centred approaches to improve BP control outside traditional facility-based care platforms [44]. However, the present study was not designed to directly compare service-delivery models, and further research is needed to determine which strategies are most effective for sustaining longitudinal HTN control in routine care settings".

9. The conclusion should be slightly softened The conclusion is broadly aligned with the results, and the concern about persistent gaps in longitudinal hypertension care is well supported.

However, the statement about the need for "integrated and targeted chronic care models that ensure treatment continuity and sustained control" is a little stronger than the evidence presented here can support. The study shows important weaknesses in current care, but it does not directly test which model of care would solve them. A slightly more cautious wording would keep the conclusion firmly anchored in the data.

Response: we revised the concluding statement to avoid implying that the present analysis directly identified or evaluated specific chronic care models capable of improving longitudinal HTN outcomes. The

revised conclusion now more cautiously emphasizes the observed gaps in HTN care and the importance of strengthening HTN screening, diagnosis, and sustained BP control within routine chronic disease and HIV care services.

Revised text:

“These findings underscore persistent gaps in longitudinal HTN care and highlight the importance of strengthening HTN screening, diagnosis, and sustained BP control within routine chronic disease and HIV care services.”

10. Data availability statements need to be aligned with PLOS requirements This is an important issue for the journal. In the submission system, the authors appear to indicate that all data are fully available without restriction, but the detailed data statement says the data are not publicly available and may only be accessed upon request and approval. In the manuscript itself, the data availability statement says simply that the data are available from the corresponding author on reasonable request.

These statements are not consistent with one another and should be corrected before the paper can move forward.

Response: we revised the manuscript Data Availability Statement to more clearly reflect the controlled-access nature of the dataset, given the ethical and confidentiality restrictions associated with participant-level clinical data. We will also ensure that the corresponding selections within the submission system are updated to match the revised statement.

Revised Data Availability Statement:

“The data underlying the findings of this study are not publicly available due to ethical and confidentiality restrictions related to participant-level clinical data. De-identified data may be made available upon reasonable request to the corresponding author, subject to approval by the Human Research Ethics Committee (Medical) of the University of the Witwatersrand and any applicable data-sharing agreements.”

11. A careful editorial cleanup is still needed There are still a number of visible wording and formatting errors in the manuscript, for example:

* "between August 2022 and May 2024. with follow-up through May 2025" in the abstract,
* "suggesFted" in the introduction,
* and spacing problems such as "servicesrather."

Response: we conducted a thorough editorial review of the manuscript to correct typographical, grammatical, spacing, and formatting inconsistencies throughout the text.

The examples highlighted by the reviewer, including punctuation, spacing, and typographical errors in the abstract and main text, were corrected in the revised manuscript. We additionally reviewed the manuscript more broadly to improve overall clarity, consistency, and readability prior to resubmission.

* We ask every co-author listed on the manuscript to fill in a contributing author statement, making sure to declare all competing interests. If any of the co-authors have not filled in the statement, we will remind them to do so when the paper is revised. If all statements are not completed in a timely fashion this could hold up the re-review process. If new competing interests are declared later in the revision process, this may also hold up the submission. Should there be a problem getting one of your co-authors to fill in a

statement we will be in contact. Please do not add or remove authors without first discussing this with the handling editor. You can see our competing interests policy here: https://zaf01.safelinks.protection.outlook.com/?url=http%3A%2F%2Fjournals.plos.org%2Fplosmedicine%2Fs%2Fcompeting-interests&data=05%7C02%7Csgumede%40ezintsha.org%7C701c27ba1211402807a008de9ecb12ab%7Ca855971766f040d8803d6e989abac34c%7C0%7C0%7C639122792985978227%7CUnknown%7CTWFpbGZsb3d8eyJFbXB0eU1hcGkiOnRydWUsIlYiOiIwLjAuMDAwMCIsIlAiOiJXaW4zMiIsIkFOIjoiTWFpbCIsIldUIjoyfQ%3D%3D%7C0%7C%7C%7C&sdata=n%2BJpkpVlmw1QJdJ77bfjdovEeWwG4wwW2bbU2%2Bx6Wn0%3D&reserved=0.

Response: We acknowledge this requirement and will ensure that all co-authors complete the contributing author statements and competing interests declarations through the submission system in a timely manner. No changes to authorship are planned without prior discussion with the handling editor.

* Please upload any figures associated with your paper as individual TIF or EPS files with 300dpi resolution at resubmission; please read our figure guidelines for more information on our requirements: https://zaf01.safelinks.protection.outlook.com/?url=http%3A%2F%2Fjournals.plos.org%2Fplosmedicine%2Fs%2Ffigures&data=05%7C02%7Csgumede%40ezintsha.org%7C701c27ba1211402807a008de9ecb12ab%7Ca855971766f040d8803d6e989abac34c%7C0%7C0%7C639122792986000893%7CUnknown%7CTWFpbGZsb3d8eyJFbXB0eU1hcGkiOnRydWUsIlYiOiIwLjAuMDAwMCIsIlAiOiJXaW4zMiIsIkFOIjoiTWFpbCIsIldUIjoyfQ%3D%3D%7C0%7C%7C%7C&sdata=PkHU8%2FFTyFt7Pze%2Bqvw6X%2B8qUj8MiMU12%2BBRpNLbE9I%3D&reserved=0. While revising your submission, we strongly recommend that you use PLOS's NAAS tool (https://zaf01.safelinks.protection.outlook.com/?url=https%3A%2F%2Fngplosjournals.pagemajik.ai%2Fartanalysis&data=05%7C02%7Csgumede%40ezintsha.org%7C701c27ba1211402807a008de9ecb12ab%7Ca855971766f040d8803d6e989abac34c%7C0%7C0%7C639122792986021310%7CUnknown%7CTWFpbGZsb3d8eyJFbXB0eU1hcGkiOnRydWUsIlYiOiIwLjAuMDAwMCIsIlAiOiJXaW4zMiIsIkFOIjoiTWFpbCIsIldUIjoyfQ%3D%3D%7C0%7C%7C%7C&sdata=632H54DDbSIE4LIuMqcFg9lDxD5B5jgyrHTZRd9Xpqk%3D&reserved=0) to test your figure files. NAAS can convert your figure files to the TIFF file type and meet basic requirements (such as print size, resolution), or provide you with a report on issues that do not meet our requirements and that NAAS cannot fix.

Response: Figures have been uploaded separately as individual high-resolution figure files. All figures are submitted in the required TIFF or EPS format at 300 dpi resolution. Where applicable, the figures were processed using the PLOS NAAS tool to ensure compliance with journal requirements prior to resubmission.

After uploading your figures to PLOS's NAAS tool - https://zaf01.safelinks.protection.outlook.com/?url=https%3A%2F%2Fngplosjournals.pagemajik.ai%2Fartanalysis&data=05%7C02%7Csgumede%40ezintsha.org%7C701c27ba1211402807a008de9ecb12ab%7Ca855971766f040d8803d6e989abac34c%7C0%7C0%7C639122792986038400%7CUnknown%7CTWFpbGZsb3d8eyJFbXB0eU1hcGkiOnRydWUsIlYiOiIwLjAuMDAwMCIsIlAiOiJXaW4zMiIsIkFOIjoiTWFpbCIsIldUIjoyfQ%3D%3D%7C0%7C%7C%7C&sdata=4qn3nmY9XldcuzLcLoxbBjd1VGWqxfXrfypngqmpC1k%3D&reserved=0, NAAS will process the files provided and display the results in the "Uploaded Files" section of the page as the processing is complete.

If the uploaded figures meet our requirements (or NAAS is able to fix the files to meet our requirements), the figure will be marked as "fixed" above. If NAAS is unable to fix the files, a red "failed" label will appear above.

When NAAS has confirmed that the figure files meet our requirements, please download the file via the download option, and include these NAAS processed figure files when submitting your revised manuscript.

Response: We have used the PLOS NAAS tool to assess all figure files prior to resubmission. Where applicable, the NAAS-processed figure files that met the journal's formatting requirements were downloaded and included with the revised manuscript submission.

* Please ensure that the paper adheres to the PLOS Data Availability Policy (see https://zaf01.safelinks.protection.outlook.com/?url=http%3A%2F%2Fjournals.plos.org%2Fplosmedicine%2Fs%2Fdata-availability&data=05%7C02%7Csgumede%40ezintsha.org%7C701c27ba1211402807a008de9ecb12ab%7Ca855971766f040d8803d6e989abac34c%7C0%7C0%7C639122792986054329%7CUnknown%7CTWFpbGZsb3d8eyJFbXB0eU1hcGkiOnRydWUsIlYiOiIwLjAuMDAwMCIsIlAiOiJXaW4zMiIsIkFOIjoiTWFpbCIsIldUIjoyfQ%3D%3D%7C0%7C%7C%7C&sdata=1Q%2BseMrU2cF0KDDU3awUhnrSkdnXPuH7G81Ak%2BOebLo%3D&reserved=0), which requires that all data underlying the study's findings be provided in a repository or as Supporting Information. For data residing with a third party, authors are required to provide instructions with contact information (web or email address) for obtaining the data. Please note that a study author cannot be the contact person for the data. PLOS journals do not allow statements supported by "data not shown" or "unpublished results." For such statements, authors must provide supporting data or cite public sources that include it.

Response: Thank you for this important clarification regarding the PLOS Data Availability Policy. In response, we revised the Data Availability Statement to ensure consistency with journal requirements and to clarify the controlled-access nature of the dataset due to ethical and confidentiality restrictions associated with participant-level clinical data.

The revised statement now specifies that de-identified data may be made available to qualified researchers upon reasonable request and subject to approval by the Human Research Ethics Committee (Medical) of the University of the Witwatersrand, rather than through direct author-mediated access. We will also ensure that the corresponding information in the submission system is updated to align with the revised manuscript statement.

* We expect all researchers with submissions to PLOS in which author-generated code underpins the findings in the manuscript to make all author-generated code available without restrictions upon publication of the work. In cases where code is central to the manuscript, we may require the code to be made available as a condition of publication. Authors are responsible for ensuring that the code is reusable and well documented. Please make any custom code available, either as part of your data deposition or as a supplementary file. Please add a sentence to your data availability statement regarding any code used in the study, e.g. "The code used in the analysis is available from Github [URL] and archived in Zenodo [DOI link]" Please review our guidelines at https://zaf01.safelinks.protection.outlook.com/?url=https%3A%2F%2Fjournals.plos.org%2Fplosmedicine%2Fs%2Fmaterials-software-and-code-sharing&data=05%7C02%7Csgumede%40ezintsha.org%7C701c27ba1211402807a008de9ecb12ab%7Ca855971766f040d8803d6e989abac34c%7C0%7C0%7C639122792986070251%7CUnknown%7CTWFpbGZsb3d8eyJFbXB0eU1hcGkiOnRydWUsIlYiOiIwLjAuMDAwMCIsIlAiOiJXaW4zMiIsIkFOIjoiTWFpbCIsIldUIjoyfQ%3D%3D%7C0%7C%7C%7C&sdata=oODjuJIsBzaMZUDSRLIOn2ZkivhCNNN0MrNMdTEB3ww%3D&reserved=0 and ensure that your code is shared in a way that follows best practice and facilitates reproducibility and reuse. Because Github depositions can

be readily changed or deleted, we encourage you to make a permanent DOI'd copy (e.g. in Zenodo) and provide the URL.

Response: We have revised the Data Availability Statement to include information regarding the availability of the statistical analysis code used in the study.

The Stata code/dofile used for data cleaning and statistical analyses has been made available as Supporting Information accompanying the manuscript to facilitate reproducibility and reuse in line with PLOS requirements.

* Please provide a web address or email address for data access requests.

Response: We have revised the Data Availability Statement to include an institutional web address and email address for data access requests in line with PLOS data-sharing requirements. The revised statement now specifies that requests for access to de-identified data should be directed through the Human Research Ethics Committee (Medical) of the University of the Witwatersrand rather than through the study authors directly.

* COIs: Please declare membership of the PLOS Medicine Editorial Board where relevant.

Response: Thank you for this note. We confirm that none of the authors are members of the PLOS Medicine Editorial Board.

* Please outline consent procedures (informed consent; written or oral) for participants in the iHEART-SA trial.

Response: We have revised the Ethics Statement section to more clearly describe participant consent procedures for the iHEART-SA trial. Specifically, we now state that written informed consent was obtained from all participants prior to enrolment in the parent study. We also clarified that ethical approval for both the parent trial and the present secondary analysis was obtained from the Human Research Ethics Committee (Medical) of the University of the Witwatersrand.

* Please ensure that the study is reported according to the STROBE guideline and include the completed checklist as Supporting Information.

Response: The manuscript was reviewed and revised in accordance with the STROBE reporting guideline for observational studies. A completed STROBE checklist has been included as Supporting Information accompanying the revised manuscript. We have also added a statement in the methods section indicating that the study is reported according to the STROBE guideline.

FORMATTING - GENERAL

* Abstract: Please structure your abstract using the PLOS Medicine headings (Background, Methods and Findings, Conclusions). Please combine the Methods and Findings sections into one section.

Response: We have revised the abstract to conform to the PLOS Medicine format by using the required headings: Background, Methods and Findings, and Conclusions. The Methods and Findings sections have been combined into a single section, and the abstract has been reviewed to ensure that it remains consistent with PLOS Medicine reporting requirements.

* At this stage, we ask that you include a short, non-technical Author Summary of your research to make findings accessible to a wide audience that includes both scientists and non-scientists. The Author Summary should immediately follow the Abstract in your revised manuscript. This text is subject to editorial change and should be distinct from the scientific abstract. Ideally each sub-heading should contain

2-3 single sentence, concise bullet points containing the most salient points from your study. In the final bullet point of 'What Do These Findings Mean?', please include the main limitations of the study in non-technical language. Please see our author guidelines for more information: https://zaf01.safelinks.protection.outlook.com/?url=https%3A%2F%2Fjournals.plos.org%2Fplosmedicine%2Fs%2Frevising-your-manuscript%23loc-author-summary&data=05%7C02%7Csgumede%40ezintsha.org%7C701c27ba1211402807a008de9ecb12ab%7Ca855971766f040d8803d6e989abac34c%7C0%7C0%7C639122792986085958%7CUnknown%7CTWFpbGZsb3d8eyJFbXB0eU1hcGkiOnRydWUsIlYiOiIwLjAuMDAwMCIsIlAiOiJXaW4zMiIsIkFOIjoiTWFpbCIsIldUIjoyfQ%3D%3D%7C0%7C%7C%7C&sdata=alZTSI8n8sjrmEG9LQmie1JF8tGN02IsTV45M4kFh%2BA%3D&reserved=0.

Response: We have added a non-technical Author Summary immediately following the Abstract in the revised manuscript. The Author Summary is structured according to the requested headings ("Why Was This Study Done?", "What Did the Researchers Do and Find?", and "What Do These Findings Mean?") and consists of concise bullet points written for a broad audience. We ensured that the summary is distinct from the scientific abstract, highlights the key findings and public health relevance of the study, and includes a plain-language description of the main study limitations in the final bullet point.

* Please express the main results with 95% CIs as well as p values. When reporting p values please report as p<0.001 and where higher as the exact p value p=0.002, for example. Throughout, suggest reporting statistical information as follows to improve clarity for the reader "22% (95% CI [13%,28%]; p</=)". Please be sure to define all numerical values at first use.

Response: Thank you for this helpful recommendation. We reviewed the Abstract, Results, Tables, and Figure legends and revised the reporting of statistical results to improve clarity and consistency. Where appropriate, key estimates are now presented with their corresponding 95% confidence intervals and p-values. We also standardized p-value reporting throughout the manuscript, reporting values as p < 0.001 where applicable and as exact p-values when ≥ 0.001. In addition, we ensured that percentages and other numerical values are clearly defined at first use and that statistical results are reported consistently throughout the manuscript.

* Please include page numbers and line numbers in the manuscript file. Use continuous line numbers (do not restart the numbering on each page).

Response: The revised manuscript has been formatted to include page numbers and continuous line numbering throughout, in accordance with the journal's submission requirements.

* Please cite the reference numbers in square brackets. Citations should precede punctuation.

Response: We reviewed the manuscript and revised citation formatting throughout to ensure consistency with journal style requirements. Reference citations are now presented in square brackets and positioned before punctuation where applicable

FIGURES AND TABLES

* Please provide titles and legends for all figures and tables (including those in Supporting Information files).

Response: We reviewed all figures, tables, and Supporting Information files to ensure that each has an appropriate title and accompanying legend. Figure and table legends were revised where necessary to improve clarity, and all abbreviations used within figures, tables, and Supporting Information files have been defined in the corresponding legends.

* Please define all abbreviations used in each figure/table (including those in Supporting Information files).

Response: We reviewed all figures, tables, and Supporting Information files to ensure that abbreviations are defined in the corresponding legends and footnotes. Additional definitions were added where necessary to improve clarity and ensure consistency throughout the manuscript and supplementary materials.

* Please consider avoiding the use of red and green in order to make your figure more accessible to those with color blindness.

Response: We reviewed the colour schemes used in all figures and revised them, where necessary, to avoid red–green colour combinations and improve accessibility for readers with colour vision deficiencies. The revised figures use colour palettes that enhance readability while maintaining clear distinction between categories and outcomes.

SUPPLEMENTARY MATERIAL

* Please note that supplementary material will be posted as supplied by the authors. Therefore, please amend it according to the relevant comments outlined here.

Response: We reviewed all Supporting Information files and revised them, where necessary, to address the comments raised during peer review. We also checked the supplementary materials for consistency with the revised manuscript, including titles, formatting, and data presentation prior to resubmission.

* Please cite your Supporting Information as outlined here:
https://zaf01.safelinks.protection.outlook.com/?url=https%3A%2F%2Fjournals.plos.org%2Fplosmedicine%2Fs%2Fsupporting-information&data=05%7C02%7Csgumede%40ezintsha.org%7C701c27ba1211402807a008de9ecb12ab%7Ca855971766f040d8803d6e989abac34c%7C0%7C0%7C639122792986102136%7CUnknown%7CTWFpbGZsb3d8eyJFbXB0eU1hcGkiOnRydWUsIlYiOiIwLjAuMDAwMCIsIlAiOiJXaW4zMiIsIkFOIjoiTWFpbCIsIldUIjoyfQ%3D%3D%7C0%7C%7C%7C&sdata=SLgEla4z5wzRRxEBSYhpvMZ9Zs23WYAuLMade3i%2Fjcs%3D&reserved=0

Response: Thank you for this guidance. We reviewed the Supporting Information files and ensured that they are labelled and cited in the manuscript in accordance with PLOS Medicine requirements. References to all supplementary materials have been added at the appropriate locations in the text, and a Supporting Information section has been included to describe each file.

REFERENCES

* PLOS uses the numbered citation (citation-sequence) method and first six authors, et al.

Response: We reviewed the reference list and citation formatting throughout the manuscript to ensure compliance with PLOS Medicine reference style. References are now presented using the numbered citation-sequence format, and the reference list has been formatted to include the first six authors followed by “et al.” where applicable.

* Please ensure that journal name abbreviations match those found in the National Center for Biotechnology Information (NCBI) databases (https://zaf01.safelinks.protection.outlook.com/?url=http%3A%2F%2Fwww.ncbi.nlm.nih.gov%2Fnlmcatalog%2Fjournals&data=05%7C02%7Csgumede%40ezintsha.org%7C701c27ba1211402807a008de9ecb12ab%7Ca855971766f040d8803d6e989abac34c%7C0%7C0%7C639122792986117461%7CUnknown%7CTWFpbGZsb3d8eyJFbXB0eU1hcGkiOnRydWUsIlYiOiIwLjAuMDAwMCIsIlAiOiJXaW4zMiIsIkFOIjoiTWFpbCIsIldUIjoyfQ%3D%3D%7C0%7C%7C%7C&sdata=VEq279Xt4nsbRgQWrkKCcypqUl%2By9RJBhtLgE69uHlc%3D&reserved=0), and are appropriately formatted and capitalised.

Response: We reviewed the reference list and ensured that all journal titles are abbreviated in accordance with the National Center for Biotechnology Information (NCBI) journal abbreviation standards. We also checked the formatting and capitalization of all references to ensure consistency with PLOS Medicine reference style.

* Where website addresses are cited, please include the complete URL and specify the date of access (e.g. [accessed: 12/06/2023]).

Response: We reviewed all citations in the manuscript and reference list to ensure that complete URLs are provided and that the date on which each website was accessed is reported in accordance with PLOS Medicine requirements.

* Please also see https://zaf01.safelinks.protection.outlook.com/?url=https%3A%2F%2Fjournals.plos.org%2Fplosmedicine%2Fs%2Fsubmission-guidelines%23loc-references&data=05%7C02%7Csgumede%40ezintsha.org%7C701c27ba1211402807a008de9ecb12ab%7Ca855971766f040d8803d6e989abac34c%7C0%7C0%7C639122792986141929%7CUnknown%7CTWFpbGZsb3d8eyJFbXB0eU1hcGkiOnRydWUsIlYiOiIwLjAuMDAwMCIsIlAiOiJXaW4zMiIsIkFOIjoiTWFpbCIsIldUIjoyfQ%3D%3D%7C0%7C%7C%7C&sdata=%2B%2B1ZE61jE8dzbcu5bbUIfTPthD7NedYxfL0ARgLQmdY%3D&reserved=0 for further details on reference formatting.

STUDY TYPE-SPECIFIC REQUESTS - OBSERVATIONAL STUDIES

* Abstract: Please include the study design, population and setting, number of participants, years during which the study took place (enrollment and follow up), length of follow up, and main outcome measures.

Response: We revised the abstract to ensure that it includes the study design, study population, setting, sample size, enrolment period, follow-up period, and primary outcome measures in accordance with PLOS Medicine reporting requirements. Specifically, we clarified that the analysis was a longitudinal secondary analysis of data from the iHEART-SA trial conducted across nine public primary healthcare clinics in

Johannesburg, South Africa, with participant enrolment between August 2022 and May 2024 and follow-up through May 2025. We also ensured that the primary outcomes of progression and regression across the HTN care cascade were clearly described.

* Please ensure that the study is reported according to the STROBE (or appropriate STROBE extension) guideline (available from: https://zaf01.safelinks.protection.outlook.com/?url=https%3A%2F%2Fwww.equator-network.org%2Freporting-guidelines%2Fstrobe&data=05%7C02%7Csgumede%40ezintsha.org%7C701c27ba1211402807a008de9ecb12ab%7Ca855971766f040d8803d6e989abac34c%7C0%7C0%7C639122792986166566%7CUnknown%7CTWFpbGZsb3d8eyJFbXB0eU1hcGkiOnRydWUsIlYiOiIwLjAuMDAwMCIsIlAiOiJXaW4zMiIsIkFOIjoiTWFpbCIsIldUIjoyfQ%3D%3D%7C0%7C%7C%7C&sdata=Nvhe%2FDZnfMbD9xL79uX6eodxls8f1z7weweUn2bbyMY%3D&reserved=0) and include the completed STROBE (or STROBE extension) checklist as Supporting Information. Please add the following statement, or similar, to the Methods: "This study is reported as per the Strengthening the Reporting of Observational Studies in Epidemiology (STROBE) guideline (S1 Checklist)." When completing the checklist, please use section and paragraph numbers, rather than page numbers.

Response: The manuscript was reviewed and revised in accordance with the STROBE reporting guideline for observational studies. A completed STROBE checklist has been included as Supporting Information accompanying the revised manuscript. We have also added a statement in the Methods section indicating that the study is reported according to the STROBE guideline.

* For all observational studies, in the manuscript text, please indicate: (1) the specific hypotheses you intended to test, (2) the analytical methods by which you planned to test them, (3) the analyses you actually performed, and (4) when reported analyses differ from those that were planned, transparent explanations for differences that affect the reliability of the study's results. If a reported analysis was performed based on an interesting but unanticipated pattern in the data, please be clear that the analysis was data driven.

Response: Thank you for this important comment regarding analytical transparency. We reviewed the manuscript to ensure that the study objectives, outcomes, analytical approach, and reported analyses are clearly described. The introduction and methods sections specify the study objectives, longitudinal HTN care cascade outcomes, and statistical methods used to evaluate these outcomes. We have also clarified that the analyses presented were prespecified as part of the secondary analysis objectives and that no additional post hoc or data-driven analyses were undertaken.

* Please state in the Methods section whether the study had a prospective protocol or analysis plan. If a prospective analysis plan (from your funding proposal, IRB or other ethics committee submission, study protocol, or other planning document written before analyzing the data) was used in designing the study, please include the relevant document(s) with your revised manuscript as a Supporting Information file to be published alongside your study and cite it in the Methods section. A legend for this file should be included at the end of your manuscript. If no such document exists, please make sure that the Methods section transparently describes when analyses were planned, and when/why any data-driven changes to analyses took place. Changes in the analysis, including those made in response to peer review comments, should be identified as such in the Methods section of the paper, with rationale.

Response: The objectives, outcomes, and analytical approach for this secondary analysis were specified in a written protocol prior to conducting the analyses. In response to this request, we have included the secondary analysis protocol as supporting information (S1 Protocol) and cited it in the methods section. We also clarified that the analyses reported in the manuscript were prespecified and correspond to those outlined in the protocol.
