# Supplementary material for: The longitudinal care cascade for hypertension in people with and without HIV in South Africa: A clinic-based observational study
Source: PLoS Med. 2026 Sep 11;23(9):e1004957. doi: 10.1371/journal.pmed.1004957 (PMC13567817; doi:10.1371/journal.pmed.1004957)
Supplement: Supplementary file 6 [file pmed.1004957.s009.pdf]

Date: 14 August 2026

Manuscript Title: The longitudinal care cascade for hypertension: a clinic-based study of people with and without HIV in South Africa

Dear PLOS Medicine editor,

Please find our detailed point-by-point responses to all comments from the Editor and Reviewers.

We have carefully considered all comments and suggestions and have revised the manuscript accordingly. A detailed response to each comment is provided below. Changes made in the revised manuscript have been highlighted using track changes to facilitate review.

The revised manuscript and all responses have been reviewed and approved by all co-authors.

Sincerely,

On behalf of all authors

Dr. Siphamandla Gumede
BSc, MSc (Med), PhD
C: +27 72 362 1793 |**O**: +27 11 084 4930|**E:** sgumede@ezintsha.org |**W:** www.ezintsha.org
32 Princess of Wales Terrace, Building C, Ground Floor, Parktown, 2193, South Africa
Ezintsha is a Division of Wits Health Consortium, University of the Witwatersrand, Johannesburg

Point by Point responses to editors' and reviewers' comments

1) We have noticed that you have uploaded "S1 Protocol,S1 STATA Dofile, S1 STROBE Checklist" files as supporting information but you have not included them in the list of legends. Please add a citation of this SI files into the list of legends of your supporting information files.

Response: We have revised the Supporting Information section to ensure that all Supporting Information files are appropriately cited and formatted. Specifically, we have added entries for S1 Protocol, S1 Stata Dofile, S1 Checklist, and S2 Table each with an appropriate title and accompanying legend describing the content and purpose of the file. These are cited in the manuscript where relevant.

2) Some material included in your submission may be copyrighted. According to PLOS's copyright policy, authors who use figures or other material (e.g., graphics, clipart, maps) from another author or copyright holder must demonstrate or obtain permission to publish this material under the Creative Commons Attribution 4.0 International (CC BY 4.0) License used by PLOS journals. Please closely review the details of PLOS's copyright requirements here: PLOS Licenses and Copyright. If you need to request permissions from a copyright holder, you may use PLOS's Copyright Content Permission form.

Response: We have addressed the concern by removing the previously used downloaded map/base layer and redesigning the map. The revised map does not use the previously downloaded proprietary or copyrighted base layer. We have also reviewed the revised figure to ensure that it does not incorporate any proprietary mapping imagery or copyrighted map tiles.

Potential Copyright Issues:

[S1 Protocol] contains branding/a logo. We are not permitted to publish this under our CC BY 4.0 license, even with permission. We ask that you please remove or replace it.

Response: We have removed the Ezintsha logo, which previously appeared in each page of **S1 Protocol**. The revised protocol has been uploaded with this resubmission and no longer contains any institutional logos or branding graphics.

[S1 Protocol]: please (a) provide a direct link to the base layer of the map (i.e., the country or region border shape) and ensure this is also included in the figure legend; and (b) provide a link to the terms of use / license information for the base layer image or shapefile. We cannot publish proprietary or copyrighted maps (e.g. Google Maps, Mapquest) and the terms of use for your map base layer must be compatible with our CC BY 4.0 license.

Response: We have addressed the concern by removing the previously used downloaded map/base layer and redesigning the map. The revised map does not use the previously downloaded proprietary or copyrighted base layer. We have also reviewed the revised figure to ensure that it does not incorporate any proprietary mapping imagery or copyrighted map tiles.

Note: if you created the map in a software program like R or ArcGIS, please locate and indicate the source of the basemap shapefile onto which data has been plotted.

If your map was obtained from a copyrighted source please amend the figure so that the base map used is from an openly available source. Alternatively, please provide explicit written permission from the copyright holder granting you the right to publish the material under our CC BY 4.0 license.

Response: We have addressed the concern by removing the previously used downloaded map/base layer and redesigning the map. The revised map does not use the previously downloaded proprietary or copyrighted base layer. We have also reviewed the revised figure to ensure that it does not incorporate any proprietary mapping imagery or copyrighted map tiles.

Pleaser remove the horizontal lines in Figures 2 and 3.

Response: We have removed the horizontal grid lines from Figures 2 and 3.

In Figure 4, as the coloured scale bar includes purple, please remove the background purple in the table that consists of formatting.

Response: We have removed the purple background formatting from **Figure 4**

Requests from Editors:

GENERAL EDITORIAL REQUESTS

* Your title must comply with PLOS Medicine's style. Your title must be nondeclarative and not a question. It should begin with main concept if possible. "Effect of" should be used only if causality can be inferred, i.e., for an RCT. Please revise your title to place the study design (only) ("A randomized controlled trial," "A retrospective study," "A modelling study," etc.) in the subtitle (ie, after a colon).

* Please confirm that your abstract complies with our requirements, including format (three sections: Background, Methods and Findings, and Conclusions) and providing all the information relevant to this study type https://zaf01.safelinks.protection.outlook.com/?url=https%3A%2F%2Fjournals.plos.org%2Fplosmedicine%2Fs%2Fsubmission-guidelines%23loc-abstract&data=05%7C02%7Csgumede%40ezintsha.org%7Ca1eade7ec32e43e685f208def1ae69f3%7Ca855971766f040d8803d6e989abac34c%7C0%7C0%7C639213928606277656%7CUnknown%7CTWFpbGZsb3d8eyJFbXB0eU1hcGkiOnRydWUsIlYiOiIwLjAuMDAwMCIsIlAiOiJXaW4zMiIsIkFOIjoiTWFpbCIsIldUIjoyfQ%3D%3D%7C0%7C%7C%7C&sdata=klypkhcKLbivBv3k5p25ONFDQDqw7uIBc888sHYacDQ%3D&reserved=0

* Please ensure that the Introduction ends with a clear description of the study question or hypothesis.

Response: We have revised the final paragraph of the Introduction to make the study question explicit. The revised text now clearly states that we examined whether progression and regression across stages of the HTN care cascade differed by HIV status.

Section revised: Introduction

Revised text:

In this study, we use a large clinical dataset from the Integrating HIV and Heart Health in South Africa (iHEART-SA) trial to provide a comprehensive description and comparison of progression and regression across stages of the HTN care cascade among PLHIV and PNLHIV in Johannesburg, South Africa. We examined whether progression and regression across the HTN care cascade differed by HIV status.

* Please ensure that all abbreviations are defined at first use throughout the text.

Response: We have carefully reviewed the manuscript to ensure that all abbreviations are defined at their first use in the main text. We have also checked the Abstract, tables, figures, and Supporting Information to ensure consistent use and definition of abbreviations throughout.

* Please confirm that all numbers presented in the abstract are present and identical to numbers presented in the main manuscript text.

Response: We have carefully reviewed all numerical values reported in the Abstract against the corresponding results in the main manuscript text and confirmed that the reported numbers are consistent and identical throughout.

GENERAL

* Please include a statement regarding use or non-use of AI tools and technologies at the end of the Methods. Please specify the name(s) and version(s) of any tool(s) used and a description of their use, including how the AI-assisted or generated content was evaluated by authors, and a clear statement of which aspects of the study, article contents, data, or supporting files were affected or generated by AI tool usage. Please also include a statement specifying who retains responsibility for the final manuscript. See our guidelines for more information: https://zaf01.safelinks.protection.outlook.com/?url=https%3A%2F%2Fjournals.plos.org%2Fplosmedicine%2Fs%2Fethical-publishing-practice%23loc-artificial-intelligence-tools-and-technologies&data=05%7C02%7Csgumede%40ezintsha.org%7Ca1eade7ec32e43e685f208def1ae69f3%7Ca855971766f040d8803d6e989abac34c%7C0%7C0%7C639213928606289927%7CUnknown%7CTWFpbGZsb3d8eyJFbXB0eU1hcGkiOnRydWUsIlYiOiIwLjAuMDAwMCIsIlAiOiJXaW4zMiIsIkFOIjoiTWFpbCIsIldUIjoyfQ%3D%3D%7C0%7C%7C%7C&sdata=3LAwVS3i0IXBeXobBB9282bAzV08uju0lJlbAZkA1Fs%3D&reserved=0.

Response: We have added a statement at the end of the methods describing the use of artificial intelligence (AI) tools during manuscript preparation, in accordance with the *PLOS Medicine* guidelines. The statement specifies the AI tool used, its purpose, the aspects of the study and manuscript that were not assisted by AI, how AI-assisted content was reviewed by the authors, and confirms that the authors retain full responsibility for the final manuscript.

Section revised: Methods

Artificial intelligence (AI) tools and technologies

OpenAI ChatGPT (GPT-5.5) was used to assist with language editing, improving clarity, and formatting. Claude (Anthropic; Claude Sonnet 4.6) was used to cross-check the consistency of data against the study's Stata dofile script. AI tools were not used for study design, data collection, data management, statistical analyses, interpretation of the findings, or generation or modification of the study results. All AI-assisted content was critically reviewed, verified against the original analyses and source materials, and revised by the authors as necessary. The authors retain full responsibility for the accuracy, integrity, and final content of the manuscript.

* Please remove the 'conclusions' subheading from the discussion. Please also remove any other subheadings from the discussion.

Response: We thank the Editor for this suggestion. We have removed the "Conclusions" subheading, along with all other subheadings, from the Discussion section in accordance with the journal's formatting requirements.

* In the last sentence of the Abstract Methods and Findings section, please describe the main limitation(s) of the study's methodology.

Response: In accordance with the *PLOS Medicine* abstract requirements, we have added a concluding sentence to the Methods and Findings section summarizing the main limitations of the study.

Section revised: Abstract – Methods and Findings

Revised text:

The main study limitations were the use of routinely collected clinical data, variable follow-up, and treatment ascertainment based on documented medication use.

* In the author summary, in the final bullet point of 'What Do These Findings Mean?', please include the main limitations of the study in non-technical language.

Response: We have revised the final bullet point under "What Do These Findings Mean?" in the Author Summary to include the principal study limitations in clear, non-technical language, while retaining the key public health implications of the findings.

Revised text:

• Because this study used routine healthcare records and followed people for different lengths of time, it cannot fully explain why these differences occurred. Even so, the findings highlight the need to strengthen hypertension care within routine chronic disease services and to better understand why people move forward or backward along the hypertension care pathway.

FUNDING STATEMENT

* The funding statement should include: specific grant numbers, initials of authors who received each award, URLs to sponsors' websites. Also, please state whether any sponsors or funders (other than the named authors) played any role in study design, data collection and analysis, the decision to publish, or preparation of the manuscript. If they had no role in the research, include this sentence: "The funders had no role in study design, data collection and analysis, decision to publish, or preparation of the manuscript."

* Please ensure that the funding statement in the manuscript matches that in the metadata. They are presently discrepant.

Response: We have revised the funding statement in accordance with the *PLOS Medicine* author guidelines. Specifically, the revised statement now includes the funding agency, the award number, the initials of the author who received the award (SG), and the funder's website. We have also included the standard NIH disclaimer and the required statement clarifying that the funders had no role in the study design, data collection and analysis, decision to publish, or preparation of the manuscript. In addition, we have reviewed and updated the funding information in the submission metadata to ensure that it is fully consistent with the Funding statement in the manuscript.

Revised text:

This work was supported by the National Heart, Lung, and Blood Institute of the National Institutes of Health through the Small Research Projects (SRP) grant (Award No. R01 5U24HL154426) awarded to SG.

Information about the funder is available at https://www.nhlbi.nih.gov/. The content is solely the responsibility of the authors and does not necessarily represent the official views of the National Institutes of Health. The funders had no role in study design, data collection and analysis, decision to publish, or preparation of the manuscript.

COMPETING INTERESTS STATEMENT

* All authors must declare their relevant competing interests per the PLOS policy, which can be seen here: https://zaf01.safelinks.protection.outlook.com/?url=https%3A%2F%2Fjournals.plos.org%2Fplosmedicine%2Fs%2Fcompeting-interests&data=05%7C02%7Csgumede%40ezintsha.org%7Ca1eade7ec32e43e685f208def1ae69f3%7Ca855971766f040d8803d6e989abac34c%7C0%7C0%7C639213928606302716%7CUnknown%7CTWFpbGZsb3d8eyJFbXB0eU1hcGkiOnRydWUsIlYiOiIwLjAuMDAwMCIsIlAiOiJXaW4zMiIsIkFOIjoiTWFpbCIsIldUIjoyfQ%3D%3D%7C0%7C%7C%7C&sdata=brozbCiRwLYhSYKFwz%2BELJwsyulJtqF8PwkWrsQMXs0%3D&reserved=0 For authors with ties to industry, please indicate whether any of the interests has a financial stake in the results of the current study.

* Please add this statement to the manuscript's Competing Interests: "JMG is an Academic Editor on PLOS Medicine's editorial board.

Response: We have revised the Competing Interests statement in accordance with the *PLOS Medicine* policy. Specifically, we have added the required disclosure as follows:

We have read the journal's policy and the authors of this manuscript have the following competing interests: SG reported receiving institutional grant funding from the National Institutes of Health and NIH Small Research Projects. JMG is an Academic Editor on the PLOS Medicine editorial board, a Consultant to the WHO Department of Noncommunicable Diseases and the Pan American Health Organization, and conducts research funded by the US National Institutes of Health. EKO reported receiving institutional grant funding from NIH Small Research Projects and the Bill & Melinda Gates Foundation. NB reported receiving NIH funding as a co-investigator on an HIV and comorbidities syndemics study and as principal investigator on an HIV and aging syndemics R21. She also provided expert witness testimony for an asylum claim before a British tribunal in 2026 and serves as an Editor of Discover Social Science and Health. AB reported research funding from the US National Institutes of Health, the American Heart Association, and Boston University and serves as Deputy Editor of the Journal of the International AIDS Society. SL-E reported receiving institutional grant funding from the National Institutes of Health, the Bill & Melinda Gates Foundation, the UK Research and Innovation Medical Research Council, and the South African Medical Research Council. No other competing interests to declare.

DATA AVAILABILITY

* PLOS defines the “minimal data set” to consist of the data set used to reach the conclusions drawn in the manuscript with related metadata and methods, and any additional data required to replicate the reported study findings in their entirety. Authors do not need to submit their entire data set, or the raw data collected during an investigation. Please submit the following data:

The values behind the means, standard deviations and other measures reported; The values used to build graphs; The points extracted from images for analysis.

Response: In accordance with the *PLOS Medicine* data sharing policy, we have prepared and submitted the minimal dataset required to reproduce the analyses and figures presented in the manuscript. The submitted dataset contains the de-identified values underlying the summary statistics, tables, and figures reported in the manuscript, together with the accompanying metadata and the Stata do-file used for data cleaning and statistical analyses. These materials are sufficient to reproduce the reported findings while protecting participant confidentiality.

Section revised: Data Availability Statement and **Supporting Information**

Revised text:

The minimal de-identified dataset underlying the results reported in this manuscript, together with the accompanying metadata and the Stata do-file used for data cleaning and statistical analyses, has been provided as Supporting Information to enable reproduction of the reported findings.

FIGURES

* Please provide titles and legends for all figures and tables (including those in Supporting Information files). Please define all acronyms used in each figure or table in its corresponding legend.

Response: We have reviewed all figures, tables, and Supporting Information files to ensure that each includes an appropriate title and legend. We have also revised the legends, where necessary, to define all abbreviations and acronyms used in the corresponding figure, table, or supporting file, in accordance with the *PLOS Medicine* guidelines.

Figures 1–4; Tables 1–3; Supporting Information (S1 Fig, S1 Protocol, S1 Stata Dofile, S2 Table, S1 Checklist, and S1 Appendix)

* Please provide the unadjusted comparisons as well as the adjusted comparisons in all relevant Tables

Response: Table 3 was the only table in the manuscript presenting regression-based comparisons of progression and regression across the HTN care cascade. We have therefore split Table 3 into two tables to present the unadjusted and adjusted estimates clearly: Table 3a now reports unadjusted (crude) risk ratios (RRs), while Table 3b reports the adjusted risk ratios (aRRs) previously presented. The two tables have an identical structure, allowing direct comparison of the unadjusted and adjusted estimates across the five modelled outcomes and four covariates; the diagnosed, not yet treated outcome remains descriptive only because of the small number of participants in this category (n=24). Unadjusted RRs were estimated using univariable Poisson regression with clinic-level clustering, while adjusted RRs were estimated using multivariable Poisson regression with the same clinic-level clustering and adjustment for the prespecified covariates. The unadjusted and adjusted estimates were generally consistent in direction, although the magnitude of some associations changed after adjustment.

Section revised: Table 3, split into Table 3a (unadjusted) and Table 3b (adjusted).

* Please specify the variables controlled for in all relevant Tables.

Response: Table 3b is the only table presenting adjusted estimates. We have added a statement directly below the table title specifying the variables included in the multivariable models and the use of clinic-level clustering. We also clarify that each covariate-specific aRR is adjusted for the other prespecified covariates in the same model. The same four covariates—HIV status, age category, sex, and nationality—were included across all five modelled outcomes; the diagnosed, not yet treated outcome was not modelled because of the small number of participants (n=24).

Section revised: Table 3b

Revised text:

Variables controlled for: All adjusted risk ratios (aRR) in this table were estimated using multivariable Poisson regression models adjusting for HIV status, age category (18–39, 40–49, 50–59, 60+), sex, and nationality (South African, Zimbabwean, Other), with clinic-level clustering. Each covariate-specific aRR reflects the association between that covariate and the outcome after adjustment for the other three covariates in the same model. The same four covariates were included across all five modelled outcomes. The diagnosed, not yet treated outcome was not modelled because of the small number of participants (n=24).

OBSERVATIONAL, COHORT, CROSS-SECTIONAL, AND CASE CONTROL STUDIES

* Please ensure that the study is reported according to the STROBE guideline, and include the completed STROBE checklist as Supporting Information. Please add the following statement, or similar, to the Methods: "This study is reported as per the Strengthening the Reporting of Observational Studies in Epidemiology (STROBE) guideline (S1 Checklist)."

Response: We thank the Editor for this reminder. The study has been reported in accordance with the Strengthening the Reporting of Observational Studies in Epidemiology (STROBE) guideline, and the completed STROBE checklist has been retained as S1 Checklist in the Supporting Information. We have also added the requested statement to the Methods to explicitly indicate adherence to the STROBE guideline.

Section revised: Methods – Data source

Revised text:

This study is reported in accordance with the Strengthening the Reporting of Observational Studies in Epidemiology (STROBE) guideline (S1 STROBE Checklist)

When completing the checklist, please use section and paragraph numbers, rather than page numbers.

Response: We have revised the STROBE checklist to provide the relevant section and paragraph numbers of the manuscript, rather than page numbers, to facilitate identification of the corresponding information.

* Did your study have a prospective protocol or analysis plan? Please state this (either way) early in the Methods section.

Response: We have clarified early in the Methods that the study objectives, HTN care cascade outcomes, and analytical approach were specified in a written secondary analysis protocol developed prior to conducting the analyses, which is provided as S1 Protocol.

Section revised: Methods – Data source

Revised text: *The study objectives, HTN care cascade outcomes, and analytical approach were specified in a written secondary analysis protocol developed prior to conducting the analyses (S1 Protocol).*

a) If a prospective analysis plan (from your funding proposal, IRB or other ethics committee submission, study protocol, or other planning document written before analyzing the data) was used in designing the study, please include the relevant prospectively written document with your revised manuscript as a Supporting Information file to be published alongside your study, and cite it in the Methods section. A legend for this file should be included at the end of your manuscript.

Response: study was a secondary analysis of longitudinal data collected through the iHEART-SA study and was not a prospectively designed cohort study. However, the objectives, HTN care cascade outcomes, and analytical approach for this secondary analysis were specified in a written secondary analysis protocol developed prior to conducting the analyses. We have retained this protocol as S1 Protocol in the Supporting Information and have cited it in the Methods section.

b) If no such document exists, please make sure that the Methods section transparently describes when analyses were planned, and when/why any data-driven changes to analyses took place.

Response: A written secondary analysis protocol specifying the study objectives, HTN care cascade outcomes, and analytical approach was developed prior to conducting the analyses and is provided as S1 Protocol in the Supporting Information. We have retained this document with the revised submission and cited it in the Methods. The study was a secondary analysis of longitudinal data from the iHEART-SA study rather than a prospectively designed cohort study.

Section revised: Methods – Data source

Revised text:

The study objectives, HTN care cascade outcomes, and analytical approach were specified in a written secondary analysis protocol developed prior to conducting the analyses (S1 Protocol).

c) In either case, changes in the analysis-- including those made in response to peer review comments-- should be identified as such in the Methods section of the paper, with rationale.

* Your study is observational and therefore causality cannot be inferred. Please remove language that implies causality and refer to associations instead.

Response: We agree that, given the observational design of this secondary analysis, the findings should be interpreted as associations and should not imply causality. We have therefore carefully reviewed the manuscript and revised causal or potentially causal language throughout, including the Abstract, Results, Discussion, and Conclusions, where necessary. We now consistently describe the relationships between HIV status, participant characteristics, and HTN care cascade outcomes as associations and have avoided wording that could imply causal effects.

* For all observational studies, in the manuscript text, please indicate: (1) the specific hypotheses you intended to test, (2) the analytical methods by which you planned to test them, (3) the analyses you actually performed, and (4) when reported analyses differ from those that were planned, transparent explanations for differences that affect the reliability of the study's results. If a reported analysis was performed based on an interesting but unanticipated pattern in the data, please be clear that the analysis was data-driven.

Response: We have revised the Methods to make the prespecified hypothesis, planned analytical approach, and analyses performed more explicit. The study hypothesis was that patterns of progression and regression across the HTN care cascade would differ by HIV status, with HIV status specified as the primary exposure. The secondary analysis protocol, developed before the analyses were conducted, specified the study objectives, HTN care cascade outcomes, and analytical approach. We have also clarified that the analyses reported in the manuscript were conducted according to the prespecified approach and that no additional post hoc or data-driven analyses were undertaken. The analytical methods used, including Poisson regression with clinic-level clustering and adjustment for age, sex, and nationality, are described in the Statistical Analyses section. The unadjusted estimates now presented in Table 3a were added to provide the unadjusted comparisons requested by the Editor; the underlying outcome definitions and regression framework were unchanged.

Sections revised: Methods – Data source/Study design and Statistical analyses

Revised text:

"The prespecified hypothesis was that patterns of progression and regression across the HTN care cascade would differ by HIV status, with HIV status being the primary exposure of interest."

"The analyses presented in this manuscript were prespecified as part of the secondary analysis objectives and were conducted as described in the protocol and Methods. No additional post hoc or data-driven analyses were undertaken."

<u>Comments from Reviewers:</u>

Reviewer #1: I thank the authors for the obvious care and effort they have put into replying to my review comments. I note the paper is much stronger this time around and is much closer to being publishable. That said, I feel there are a few items that still require attention primarily to address apparent inconsistencies and to improve interpretability. I have preserved the numbering from my original review for clarity and, where new items were required, I have added to the end of the prior numbering. If I have not referenced an item below, please consider it closed. All items are major unless noted otherwise.

4. Thank you for clarifying that participants were included in this study if they had baseline data and at least one follow-up. However, Table 2 indicated only ~66% of participants had one or more follow-up visits, suggesting patients with no follow up were included in the study. I suggest the authors remediate this inconsistency.

Response: We agree that our previous wording could be interpreted to mean that all participants included in the study had at least one follow-up visit, whereas Table 2 correctly reports that only 65.2% of participants had one or more documented follow-up visits. The descriptive analyses included all participants with baseline data and a completed medical record review (N = 23,855), while the longitudinal progression and regression analyses were conducted only among participants eligible for each transition outcome based on their baseline HTN care cascade stage and the availability of follow-up clinical information. We have revised the **Methods** to clarify this analytical approach and to explain why denominators differ across the longitudinal analyses.

Section revised: Methods – Data source

Revised text:

"Participants with baseline data and a completed medical record review were included in the descriptive analyses. Longitudinal progression and regression analyses were subsequently conducted among participants eligible for each transition outcome, based on their baseline HTN care cascade stage and the

availability of follow-up clinical information. Consequently, denominators differed across the longitudinal analyses."

Section revised: Methods – Statistical analyses

Revised text:

Analyses for each cascade outcome were restricted to participants eligible for the relevant transition stage and with available follow-up information for assessment of that transition. Consequently, the denominator varied across longitudinal outcomes according to baseline cascade stage, follow-up availability, and completeness of outcome-specific data.

5. This is enormously improved. Well done!

Response: Noted with thanks

6. I appreciate the authors intent on this point, but I could not find "S2 Table" in the submitted package. I note the inclusion of an Excel spreadsheet as "S1 Appendix" but found this difficult to read and understand. I suggest the authors provide a simple list of included hypertensive medications.

Response: To improve clarity and transparency, we have added a new Supporting Information file (**S2 Table**) that provides a simple list of the antihypertensive medications used to classify treatment status in the longitudinal hypertension care cascade analysis. The REDCap data dictionary (S1 Appendix) has been retained to support reproducibility by documenting the underlying data collection variables and coding structure.

Regarding the reviewer's related concern about the precision of our medication-identification strategy: the abstraction instrument offered five pre-specified medications, all standard first-line agents for hypertension in South African public-sector primary care: hydrochlorothiazide (ATC C03AA03), enalapril (ATC C09AA02), amlodipine (ATC C08CA01), furosemide (ATC C03CA01), and atenolol (ATC C07AB03). These codes, together with drug classes and recorded dose bands, are included in S2 Table. The form also included free-text "Other" fields for medications outside this list; each such entry was reviewed in real time during data abstraction by a clinician or nurse on the study team, who cross-checked the recorded medication against the patient's documented diagnosis/indication in the medical record to confirm hypertension-related use before it was classified as antihypertensive treatment.

We have revised the manuscript to make this explicit in the following locations:

Section: Methods → Subsection: "Defining longitudinal HTN care cascade"

Revised text :

….which captured antihypertensive agents routinely prescribed within South African public sector primary healthcare services during the study period, using five pre-specified medications (hydrochlorothiazide, enalapril, amlodipine, furosemide, and atenolol; ATC codes provided in S2 Table). Medications recorded outside this list were captured via free-text fields and reviewed in real time by a clinician or nurse on the study team, who cross-checked each entry against the patient's documented diagnosis/indication in the medical record to confirm hypertension-related use prior to classification as antihypertensive treatment (S1 Appendix).

Section: Supporting Information Caption (new entry, following S1 Appendix)

New text added:

3. S2 Table. List of hypertensive medications captured in the iHEART-SA medical record abstraction instrument. Legends: This table lists the five pre-specified antihypertensive medications available in the medical record abstraction instrument (hydrochlorothiazide, enalapril, amlodipine, furosemide, and atenolol), their ATC codes, drug classes, and recorded dose bands. Medications outside this list were captured via free-text fields and verified by clinical staff against the patient's documented diagnosis/indication before classification as antihypertensive treatment (S1 Appendix).

13. [MINOR] Again, this is much improved. The only thing I would flag is the relatively small number of clusters. Generally, 30-40 clusters are advisable to ensure validity of the robust method used by the authors. That said, in this case it is unlikely this would meaningfully impact the clinical conclusions presented. As such, I would suggest (but not insist) the authors very briefly note in the limitations that with only nine clusters the cluster-robust intervals may be modestly anticonservative, so borderline findings should be interpreted with care. The authors may wish to familiarise themselves with statistical tools (e.g., wild cluster bootstrap) that are valid for small cluster counts for situations at greater risk of anticonservative results such as cluster randomised trials.

Response: We thank the reviewer for this thoughtful and constructive suggestion. We agree that the relatively small number of clusters (nine clinics) is an important methodological consideration when using cluster-robust variance estimation. Although we do not expect this to materially affect the overall interpretation of our findings, we agree that this limitation should be acknowledged explicitly. We have therefore expanded the Limitations section to note that cluster-robust confidence intervals may be modestly anticonservative when the number of clusters is small and that borderline findings should be interpreted with caution. We have also highlighted that future studies could consider statistical methods specifically developed for analyses with few clusters, such as the wild cluster bootstrap, to further evaluate the robustness of the findings.

Section revised: Discussion – Limitations

Revised text:

An additional methodological limitation relates to the relatively small number of clusters (nine clinics) used to estimate cluster-robust standard errors in the regression models. Cluster-robust variance estimation generally performs best with a larger number of clusters (commonly 30–40 or more); with fewer clusters, confidence intervals may be modestly anticonservative, and borderline findings should therefore be interpreted with caution. However, the principal findings were consistent across analyses and supported by relatively precise effect estimates for the primary outcomes, suggesting that this limitation is unlikely to materially affect the overall interpretation of the results. Future studies could consider statistical methods specifically developed for analyses with few clusters, such as the wild cluster bootstrap, to further evaluate the robustness of these findings.

22. I found Table 3 quite difficult to interpret. Part of the problem is in some columns aRRs > 1 are desirable whereas in other columns it's the opposite. Also, it is not at all clear what the denominators are and, accordingly, what the percentages mean. To assist with both of these issues, I suggest the authors make the numerator and denominator abundantly clear for each column, similar to what was presented in Figures 2 and 3. I would suggest this is prominent in the table (i.e., not just a table note) as this is essential to interpreting the results and is easily confused. Finally, I think the use of the word "events" in the column headings added to the confusion and I suggest it is dropped. This is because, again, in some cases the "event" is desirable and others undesirable.

Response: We thank the reviewer for this detailed and constructive feedback. We have substantially revised Tables 3a and 3b to improve its clarity and interpretation.

First, we have clarified the presentation of the descriptive data by explicitly defining the numerator and denominator for each outcome in the column headers. We have also added an interpretive note immediately above the table explaining that the **n (%)** values describe the composition of each outcome group across covariate strata and should not be interpreted as stratum-specific risks. Instead, the relative likelihood of each outcome is represented by the adjusted risk ratio (aRR) estimated from the multivariable Poisson regression models.

Second, to address the ambiguity regarding interpretation of the aRRs, each outcome column now explicitly indicates whether a higher aRR represents a **more favourable** or **less favourable** outcome. The table legend has also been expanded to explain that favourable outcomes correspond to progression through the HTN care cascade (e.g., receiving treatment or achieving BP control), whereas less favourable outcomes correspond to remaining in, or regressing to, less desirable cascade states.

Third, we have revised the column headings to improve consistency and readability by replacing the term **"events"** with **"n (%) of outcome group,"** which more accurately reflects the descriptive nature of these data.

23. There appear to be some inconsistencies with participant numbers across the manuscript, tables, and figures. I note the authors make a point of saying in a number of places that similar denominators vary due to completeness etc. but my concern is when ostensibly exactly the same descriptor is associated with differing participant counts. For example, number of participants with high BP in Table 2 is different to Figure 2, Figure 3 has different denominators between the second and third pairs of bars for the same descriptor "% of those treated and controlled at enrolment", and regressed to untreated counts seem to be inconsistent between Figure 3 and Table 3. I am sympathetic that there are a lot of counts being managed in this manuscript, but it is important that all the numbers with the same descriptor agree otherwise it undermines the presented research.

Response: We thank the reviewer for identifying these inconsistencies and agree that consistent reporting of denominators across the manuscript is essential. We have carefully reviewed the relevant tables and figures and addressed each of the issues raised.

Table 2 vs. Figure 2 ("high BP" count): We confirmed that the blood pressure categorisation reported in the original Table 2 was inconsistent with the values presented in Figure 2. To avoid duplication and ensure consistency, we have removed the blood pressure categorisation from Table 2. Figure 2 now provides the sole presentation of the baseline high BP population used in the HTN care cascade analyses.

Figure 3, panels 2 and 3 (denominator for participants treated and controlled at enrolment): We confirm that the denominators used in these two panels were inconsistent in the original submission. This has been corrected, and both panels now use the same baseline population (**PLHIV:** *n* = 1,125; **PNLHIV:** *n* = 2,141).

Figure 3 vs. Table 3 ("regressed to untreated"): We reviewed the underlying data and reconciled the counts presented in both the figure and the table. We have also clarified in the **Table 3 legend** that participants contributing to each cascade stage include both incident and prevalent cases. Consequently, the reported counts represent the combined study population at each stage rather than incident cases alone.

24. While the limitations para at line 483 is a good start, it misses a key point. Endpoint ascertainment is driven by visits, not follow-up time, and PNLHIV are more likely to have more visits and, accordingly, more progressions and regressions. Suggest this differential ascertainment is added as a limitation to that para.

Response: We have expanded the **Limitations** section to acknowledge that ascertainment of progression and regression depended not only on the duration of follow-up but also on the frequency of documented clinical encounters. As participants not living with HIV had a higher median number of follow-up visits than people living with HIV (Table 2), differences in visit frequency may have increased opportunities to observe transitions across the HTN care cascade. We also identified that this paragraph had been inadvertently duplicated in the Discussion (as noted elsewhere by the reviewer) and have removed the duplicate paragraph.

Section revised: Discussion – Limitations

Revised text:

In addition, ascertainment of progression and regression depended on the frequency of documented clinical encounters at which BP and treatment status were recorded, rather than follow-up duration alone. PNLHIV had a higher median number of documented follow-up visits than PLHIV (median 3 [IQR 1–5] vs. 2 [IQR 1–4]; Table 2), providing greater opportunity for transitions across the HTN care cascade to be observed. Consequently, differences in visit frequency should be considered when interpreting comparisons in progression and regression between the two groups.

25. [MINOR] It appears a para in the Discussion was included twice (starting at lines 483 and 492). Suggest deleting the duplicate.

Response: We thank the reviewer for carefully identifying the duplicated paragraph. We have removed the duplicated paragraph from the **discussion** section.

Reviewer #2:

There are minor proofreading issues for copyediting (for example, line 470-472 should be should be reviewed for clarity and adding a slash between and and or, "For example, BP measurements and HTN diagnoses and treatment initiation may not always be recorded accurately or completely, and/or discontinuation of treatment." Also, a paragraph has been duplicated (lines 492-500 duplicate the paragraph immediately preceding paragraph).

Response: We thank the reviewer for carefully identifying these copyediting issues. We have revised the text for clarity, corrected the wording as suggested, and removed the duplicated paragraph from the discussion section.

Revised text:

For example, BP measurements, HTN diagnoses, treatment initiation, and/or treatment discontinuation may not always be recorded accurately or completely in routine clinical records.
