# Supplementary material for: The longitudinal care cascade for hypertension in people with and without HIV in South Africa: A clinic-based observational study
Source: PLoS Med. 2026 Sep 11;23(9):e1004957. doi: 10.1371/journal.pmed.1004957 (PMC13567817; doi:10.1371/journal.pmed.1004957)
Supplement: Peer review history

# Editor report: Decision Letter 0

**DOI:** 10.1371/journal.pmed.1004957.r001

**Authors:** Alison Farrell

5 Feb 2026

Dear Dr Gumede,

Thank you for submitting your manuscript entitled "The longitudinal care cascade for hypertension: a clinic-based study of people with and without HIV in South Africa" for consideration by PLOS Medicine.

Your manuscript has now been evaluated by the PLOS Medicine editorial staff, and I am writing to let you know that we would like to send your submission out for external peer review.

However, before we can send your manuscript to reviewers, we need you to complete your submission by providing the metadata that is required for full assessment. To this end, please login to Editorial Manager where you will find the paper in the 'Submissions Needing Revisions' folder on your homepage. Please click 'Revise Submission' from the Action Links and complete all additional questions in the submission questionnaire.

For clinical studies, please upload a copy of your trial study protocol as a supporting information file. The study protocol should be the version submitted for approval to the institutional review board or ethics committee, should include any amendments to the study protocol, as well as the date of their approval by the institutional review or ethics committee. Please also detail any deviations from the study protocol in the Methods section of your manuscript. The editors will consider the protocol and study conduct prior to a final decision for external review.

Please re-submit your manuscript within two working days, i.e. by Feb 09 2026 11:59PM.

Login to Editorial Manager here: https://www.editorialmanager.com/pmedicine

Once your full submission is complete, your paper will undergo a series of checks in preparation for peer review. Once your manuscript has passed all checks it will be sent out for review.

Feel free to email us at plosmedicine@plos.org if you have any queries relating to your submission.

Kind regards,

Alison Farrell, Ph.D.

Senior Editor

PLOS Medicine

# Peer review: Decision Letter 1

**DOI:** 10.1371/journal.pmed.1004957.r002

**Authors:** Louise Gaynor-Brook

20 Apr 2026

Dear Dr Gumede,

Many thanks for submitting your manuscript "The longitudinal care cascade for hypertension: a clinic-based study of people with and without HIV in South Africa" (PMEDICINE-D-26-00436R1) to PLOS Medicine. The paper has been reviewed by subject experts and a statistician; their comments are included below and can also be accessed here: [LINK]

After discussing the paper with the editorial team and an academic editor with relevant expertise, I'm pleased to invite you to revise the paper in response to the reviewers' comments. We plan to send the revised paper to some or all of the original reviewers, and we cannot provide any guarantees at this stage regarding publication.

In addition to these revisions, you may need to complete some formatting changes, which you will receive in a follow up email. A member of our team will be in touch with a set of requests shortly. If you do not receive a separate email within a few days, please assume that checks have been completed, and no additional changes are required.

When you upload your revision, please include a point-by-point response that addresses all of the reviewer and editorial points, indicating the changes made in the manuscript and either an excerpt of the revised text or the location (eg: page and line number) where each change can be found. Please also be sure to check the general editorial comments at the end of this letter and include these in your point-by-point response. When you resubmit your paper, please include a clean version of the paper as the main article file and a version with changes tracked as a marked-up manuscript. It may also be helpful to check the guidelines for revised papers at http://journals.plos.org/plosmedicine/s/revising-your-manuscript for any that apply to your paper.

We ask that you submit your revision by May 11 2026 11:59PM. However, if this deadline is not feasible, please contact me by email, and we can discuss a suitable alternative.

Don't hesitate to contact me directly with any questions (lgaynor@plos.org).

Best regards,

Louise

Louise Gaynor-Brook, MBBS PhD

Senior Editor

PLOS Medicine

lgaynor@plos.org

-----------------------------------------------------------

Comments from the reviewers:

Reviewer #1: This paper presents a longitudinal secondary analysis of the iHEART-SA trial to examine the hypertension care cascade in patients attending primary care clinics in Johannesburg, South Africa. Poission regression models were used to estimate the relative risk of transitioning forward and backwards through the care cascade. Opportunities for improved hypertension diagnosis and treatment were found in all patient subgroups, particularly so in those living with HIV.

This is a good initial submission on an important research topic. The authors do an excellent job of contextualising and justifying their research. I was, however, left concerned and a bit confused about how the authors defined and executed their analyses. Some parts of the analytical approach were not sufficiently clear and there appeared to be multiple instances of internal inconsistencies. I think the authors may be blurring cross-sectional state definitions with longitudinal transition outcomes but it is difficult to tell without clearer Methods. Most importantly, it's crucial the authors are clear on what, how, and why individual statistical modelling approaches are used.

Detailed feedback on the manuscript is provided below. My primary focus has been methodological, but I have made a few broader points as well. All items are major unless indicated otherwise.

1.[MINOR] I suggest the authors follow appropriate reporting guidelines for their study (e.g., https://www.equator-network.org/reporting-guidelines/strobe/) and include the completed checklist as a supplement.

2.The population for this study is patients attending primary care (not the general population), but this important qualification is omitted by the authors at key points in the manuscript e.g., the first sentence of the Discussion. While this can be inferred from the rest of the manuscript I think it's still potentially misleading to suggest the manuscript is presenting the hypertension care cascade for the general population.

3.[MINOR] I'm not sure if all the details on the original iHEART-SA trial in three paras of the Methods (lines 101-116) are necessary. I found it a little confusing and distracting as I was not sure if the info on the different populations applied directly to the current study or not. I surmise some details are superfluous given that all patients irrespective of HIV status were included per lines 117-118 and Figure S2. I suggest the authors consolidate these three paras into one, only present what is directly pertinent to the current study, and point any interested readers to other publication(s) for further detail.

4.Was there a minimum follow-up or were all patients included (including those with only one visit)? Suggest authors make this clear in the Methods.

5.I found the section "Defining longitudinal HTN care cascade" (line 124) a bit repetitive and confusing. For example, diagnosed with controlled BP is defined twice - once at line 135 and again at line 152. More troublingly, two conflicting definitions of antihypertensive treatment are provided at lines 132 and 149 (discussed in more detail below). Suggest the authors remove repetition and ensure consistency in this section, focusing specifically on the exact definitions used in this research.

6.In addition to the previous point, I am concerned about some of the definitions used in the longitudinal hypertension care cascade. There are many medicines that exhibit an antihypertensive effect but not all are routinely used for hypertension. As such, it is important that the authors present the precise search strategy they used to identify hypertension-defining medicines (e.g., ATC codes, active ingredients) either in the body of the manuscript or as a supplement. The illustrative list presented at lines 133-134 lacks precision and is not sufficient.

7.Relatedly to the two points above, in that same sentence (lines 133-134) I am a little confused by what is meant by "… documented in the medical record as taking … at any timepoint." Do the clinicians keep the list of current medicines up to date so it is clear what medicines a patient is on at each visit or was this inferred from prescribing records? I note it is difficult to ascertain whether a patient is on a medicine from prescribing records because single prescriptions can cover long time periods that may include multiple presentations for care. I am also concerned about the conflicting definition at line 152 that suggests patients are only considered treated if they are on drug at baseline. Surely this can change over time if a patient initiates therapy after the baseline visit? I suggest the authors carefully clarify this.

8.I think it is worth stating explicitly that patients can enter the cohort in any state depending on their BP and antihypertensive prescribing status at their first (baseline) visit.

9.[MINOR] I think the authors meant "stage" instead of "outcome" at "Estimates were reported for each outcome…" (line 162).

10.The paras under the section "Sample size, inclusion, and exclusion criteria" are results that should be included in the Results section, not the Methods. Instead, the authors should provide any sample size considerations and inclusion/exclusion criteria not already discussed or remove this section from the Methods entirely.

11.In the "Statistical analyses" section I am not sure what is meant by "For the tests of associations between HIV status and covariates, the Pearson chi-square test was used in case of dichotomous and categorical variables, and a t-test in case of continuous covariables." Generally, p-values should not be used in descriptive tables (see section 14a of the expanded STROBE guidelines https://journals.plos.org/plosmedicine/article?id=10.1371/journal.pmed.0040297 ). If the authors are talking about transition probabilities, HIV status is already included in the Poisson models, isn't it? Suggest the authors consider this and adjust methods and results accordingly.

12.It appears the authors are using Poisson regression to obtain relative risk estimates for their binary outcomes. While this approach can be appropriate, extra care must be taken. There are plenty of resources available on this, but the authors could start with this one https://bmcmedresmethodol.biomedcentral.com/articles/10.1186/s12874-018-0519-5. Please provide more detail on this including an appropriate citation.

13.In terms of the variables included in the statistical models, I am not yet sufficiently clear on exactly what was included and when. The authors say "…with each sociodemographic characteristic as the main exposure." (line 195) but weren't all variables included in an appropriate multivariable model? How was clustering accounted for given nine clinics contributed data? Generally speaking, enough detail should be included in the Methods so an interested reader can replicate the analyses. Including abridged modelling code as a supplement can also help when analyses for multiple endpoints are being undertaken.

14.[MINOR] I don't understand why there are extra boxes in Figure 1 below the main sequence for regressions. Aren't these regressing backwards through the four main sequence boxes?

15.[MINOR] While the approach taken by the authors is arguably valid (notwithstanding my concerns raised in other points), another approach would have been to use Markov chain methods to model state transitions. I suggest the authors consider this as an optional alternative approach. However, this is not necessarily required for this research to be publishable.

16.The authors could more explicitly consider clinical vs. statistical significance. This is particularly important in analyses of large datasets where statistical significance/narrow CIs that don't cross critical values are seen with trivially small effects e.g., "aRR 1.004; 95% CI 1.001-1.007" at line 270. Suggest the authors more clearly consider the magnitude of the effect and its meaningfulness when reporting and discussing results.

17.I suggest including in Table 1 the mean and median number of visits in addition to the number of days. Indeed, the number of visits is more informative than the follow-up in days because new information on each patient's health status is only acquired when they present for a visit.

18.[MINOR] The BP severity brackets in Table 1's note overlap and do not match the text. Suggest the authors correct the table notes.

19.Also in Table 1, for the number of days follow-up the SD and IQR do not appear to be plausible when considered together. Suggest the authors double check the numbers here.

20.The information in the Table 2 notes is important and should also be included in the body of the manuscript where these results are reported.

21.In the Discussion the following assertion was made but I don't think the authors included a statistical comparison of follow up between the two groups "… there was no statistically significant difference in follow-up duration between PLHIV and PNLHIV…"?

Reviewer #2: This is an important and timely paper filling a key gap in the literature, articulating how people with and without HIV differ where they sit in the hypertension care cascade and how they move through it over time. The main strengths are the longitudinal design and results presentation and the large sample size. The main finding that PLHIV are substantially more likely to remain undiagnosed and to regress from treatment despite frequent clinic contact is both clinically meaningful and policy-relevant. Connecting integrated care HTN-HIV policy and the WHO's mandate for integrated care programs to the need for this evidence is well thought out.

That said, the paper needs significant revisions before it can be publication-ready. These are detailed below:

1. The introduction notes that PLHIV may have higher rates of HTN diagnosis and treatment due to frequent clinical contact (lines 62-66), yet the paper's central finding is the opposite. While this contradiction is handled in the discussion, it may be worth more explicitly highlighting this contrast in the introduction. The framing that disparities may widen for non-PWH compared to PWH sounds slightly odd, so should be rephrased.

2. The enrollment criteria between PWH and non-PWH is a methodological concern. The parent trial's effectiveness population were enrolled precisely because they had elevated BP. Because non-PWH were enrolled without a BP eligibility requirement, it means the denominators and the underlying populations being compared are different. Authors should more clearly explain how this affects the comparative findings and comparisons between these two groups remain valid despite different enrollment pathways.

3. Authors should discuss explicitly whether non-PWH in this sample are representative of the broader non-PWH population with hypertension or whether they represent a selected subgroup (e.g., those already engaging with the health system for other chronic conditions).

4. While the paper acknowledges the wide variation in follow-up time (12-36 months) and no statistically significant differences are noted, it is also true that participants with longer follow-up had more opportunity to both progress and regress. This muddies the implications for rates of progression and regression. A sensitivity analysis is warranted, or this effect should be included in the limitations.

5. I appears that the parent trial was actively implementing the HTN integration intervention across the study period. This could bias the estimates in either direction. Authors should account for this in the secondary analysis or explain this in the limitations section.

6. Lines 124-141 define HTN and the cascade categories narratively and lines 142-160 appears to re-state the same information in numbered list format. The redundancy should be resolved. Additionally, the definitions of progression and regression (currently at lines 166-175) should be moved before the numbered cascade stage definitions for logical flow.

7. Consider reformatting the six cascade stage definitions as a table for clarity. The current presentation is difficult to follow because each definition includes denominator information embedded in the text. A table with columns for Stage, Definition, and Denominator would improve readability. Additionally, Figure 1 should also be repositioned to appear adjacent to these definitions rather than at the end of the methods section, where it is currently placed.

8. The single BP reading issue requires clearer explanation. Single readings are subject to white-coat effect and measurement error, and could meaningfully inflate HTN prevalence and subsequent findings. Lines 125-131 acknowledge that HTN diagnosis ideally requires two consecutive elevated readings, but in this trial only a single reading per visit was available. At which specific timepoint was the single reading used for baseline HTN classification? How were multiple readings across visits handled?

9. The choice of Poisson regression for binary outcomes rather than logistic regression should be briefly justified.

10. ART adherence and retention in HIV care are strong proxies for engagement with the health system, yet they are not included as covariates. The authors should either include these covariates or explain in the limitations why they were unavailable and what bias their absence may introduce.

11. Missing data handling should be described. The denominators in Table 2 footnotes differ from those in the main results (e.g., age and undiagnosed: n=3,811 vs. the outcome total N=3,950), but the approach to missing values is not explained. Was complete case analysis used? Was missingness related to key variables?

12. Table 2 is large and difficult to navigate. Consider moving it to supplementary material and presenting a simplified summary table or a visualization such as a forest plot or annotated heatmap that allows quick identification of meaningful associations.

13. Figure 2 needs significant revision. 1) The complementary bars for "undiagnosed" and "diagnosed" (which sum to 100%) are both shown which is visually redundant. The figure should be simplified. 2) The second bar is labeled "participants diagnosed with BP" which should read "participants diagnosed with HTN." "Diagnosed with BP" is not clinically meaningful. 3) Since denominators for PWH and non-PWH differ substantially, percentages alone can be misleading, so absolute counts (or denominators) should be added.

14. It is plausiable that vertical HIV programs explain poorer PWH outcomes but it is unclear why it is presented as the only narrative. Authors may consider exploring other angles, such as drug interactions between ART and anti-HTN meds affecting BP control, pill burden leading patients to deprioritize HTN treatment, providers not routinely checking BP during routine HIV visits.

15. The Zimbabwean nationality finding (lower regression risk, aRR 0.72) is intriguing and has potential policy implications. It warrants more substantive discussion, particularly in the context of South Africa's large migrant population.

A thorough proofread is recommended. Examples of minor issues are:

1. Reference formatting at line 58-59 appears to cite "[3,3-7]" — reference 3 appears to be listed twice.

2. Some phrasing implies that HIV status is driving cascade differences. Given the observational nature of the study and the uncontrolled confounding, the language should consistently reflect association rather than causation.

3. The methods section should specify the name of the institutional review board that provided ethical approval along with the reference number.

4. Figure 2 labeling inconsistencies in the bar labels. Some use "elevated BP," others use "high BP." This should be standardized.

5. Figure 3 would benefit from confidence intervals or p-values being added into the figure rather than only available in Table 2.

Reviewer #3: I was unable to locate the revised manuscript with tracked changes or the point-by-point response to the reviewers' comments, which makes it difficult to fully evaluate the revisions that have been implemented.

Overall, the manuscript is of good quality. However, I recommend a careful revision of the language and formatting, as some inaccuracies remain. In addition, the quality of Figures 2 and 3 should be improved, and the issues with the axis labels should be corrected.

Reviewer #4:

Thank you for the opportunity to review this manuscript. This is an important paper on a highly relevant issue for South Africa and other high HIV-burden settings. The large sample size, the comparison between people living with and without HIV, and the focus on movement across the hypertension care cascade over time are all real strengths. The topic is worth publishing in principle because there is still limited longitudinal evidence on hypertension diagnosis, treatment continuity, and blood pressure control in routine care in this region. At the same time, I do not think the manuscript is ready for publication yet, particularly for PLOS Medicine, where the methods, analysis, and interpretation need to be especially clear and robust. My main concerns are not about the importance of the question, but about how the analysis is framed, how some of the outcomes are defined, and how confidently some conclusions are drawn from the available data. I hope the comments below help strengthen what is already a potentially valuable paper.

1. The paper addresses an important question, but the analytic population is still not explained clearly enough. The study uses secondary data from the iHEART-SA trial, which was conducted across nine public clinics and had a stepped-wedge cluster design. That gives this analysis real value. However, I found it difficult at times to follow exactly who contributed to each stage of the cascade and each regression model.

The manuscript would benefit from one short, very clear paragraph in the Methods that explains, in plain language:

*What counted as baseline,

*What counted as follow-up,

*Who was included in each denominator,

*and whether the same participant could contribute to more than one transition outcome.

Some of this information is present, but it is spread across several sections and remains harder to follow than it should be for a paper of this importance.

2. Hypertension definition needs to be presented more honestly and consistently

This was one of the main areas where I felt the paper needed more clarity. The manuscript says that hypertension at baseline was defined using two consecutive high BP measurements, or a documented diagnosis, or treatment history. But it then states that, in practice, only a single BP reading per visit was available in the medical record, and there was no information on whether a confirmatory measurement had been taken. That means the paper should state more directly what definition was actually used in the analysis. At the moment, the wording gives the impression that a more rigorous BP definition was applied than the available data could support. Related to this, the BP thresholds are not presented consistently. In the Methods, high BP/HTN is defined as SBP ≥140 mmHg or DBP ≥90 mmHg, but in Table 1 footnote, it is written as SBP >140 mmHg or DBP >90 mmHg. (e.g., lines 129-131), vs. Table 1 footnote). The boundaries for normal and high-normal BP also differ slightly between sections. These definitions need to match throughout the paper.

3. Unequal follow-up time needs more careful handling

The paper is framed as a longitudinal analysis, but follow-up time varied considerably across participants. Some people contributed close to three years of observation, while others contributed only about twelve months.

This is important because the longer someone is followed, the more opportunity they have both to progress and to regress across the cascade. The manuscript does acknowledge this in the limitations, but I do not think that is enough on its own. The authors should explain more clearly why the analytic approach is still appropriate despite unequal follow-up time, or consider incorporating follow-up duration more directly into the analysis. The statement that follow-up duration did not differ significantly between PLHIV and PNLHIV is helpful, but it does not fully resolve the issue. Unequal follow-up can still affect the probability of observing transition events overall.

4. The issue of clustering by clinic should be addressed explicitly

Because this analysis comes from a parent study conducted across nine clinics in a cluster stepped-wedge design, it is important to know whether the regression models accounted for clustering at the clinic level. The statistical methods section says that Poisson regression was used and adjusted for age, sex, and nationality, but it does not say whether clinic-level clustering or correlation was taken into account. That is important because participants from the same clinic are unlikely to be independent. If clustering was not accounted for, the confidence intervals may be too narrow. At a minimum, the manuscript should state clearly what was done here.

5. Table 2 still needs a careful check

T Table 2 still needs another careful review for presentation accuracy and consistency.

For example, for age 18-39 years under the "undiagnosed" outcome, the confidence interval is shown as 1.28-139, which is clearly a typographical or formatting error. In the narrative Results section, the same estimate appears as 1.28-1.39, which is much more plausible.This may look minor, but tables like this need to be exact. More broadly, I encourage the authors to recheck all point estimates, confidence intervals, denominators, and footnotes in Table 2. Some of the adjusted risk ratios are extremely close to 1.00, and while that may be statistically correct, the paper should help the reader understand whether these findings are clinically meaningful.

6. Some outcome definitions are still difficult to interpret

The idea of looking at both progression and regression across the cascade is a strength of the paper. However, the way some outcomes are defined still makes the analysis difficult to interpret. For example, "treated and controlled" is presented as a progression outcome, while "regressed to untreated" and "regressed to uncontrolled" are treated as regression outcomes based on later status. The manuscript says that participants could experience both progression and regression during follow-up. That may reflect real clinical care, but it also means the reader needs a clearer explanation of how people were counted. It would help to state plainly:

*whether control at any follow-up visit counted,

*whether the last observed visit was used,

*and whether the same person could contribute to both a progression and a regression outcome.

Without that clarification, the interpretation of the results remains more difficult than it needs to be.

7. Some results outcomes interpretation is certain than the data allow

The central message of the paper is important and clearly worth sharing: PLHIV were more likely to remain undiagnosed, less likely to achieve BP control once treated, and more likely to regress to untreated or uncontrolled hypertension.

Where I would encourage more caution is in the discussion of why this may be happening. The manuscript mentions competing clinical priorities, therapeutic inertia, pill burden, and prioritisation of ART adherence. These are all plausible explanations, and many readers will find them believable. But they were not directly measured in this study. I suggest rephrasing these sections so they read as possible explanations rather than implied findings from the analysis itself.

8. Discussion is relevant, but it could be more focused

The discussion is thoughtful and engages with the broader literature on integrated HIV/NCD care, which is valuable. At the same time, some sections could be tighter. In particular, the discussion comparing integrated, vertical, and semi-vertical service models is interesting, but it goes somewhat beyond what this dataset can directly answer. I think the paper would be stronger if the discussion stayed a little closer to three things:

*What this study clearly shows,

*what it cannot explain,

*and what the most reasonable service-delivery implications are.

That would make the message sharper and more persuasive.

9. The conclusion should be slightly softened

The conclusion is broadly aligned with the results, and the concern about persistent gaps in longitudinal hypertension care is well supported.

However, the statement about the need for "integrated and targeted chronic care models that ensure treatment continuity and sustained control" is a little stronger than the evidence presented here can support. The study shows important weaknesses in current care, but it does not directly test which model of care would solve them. A slightly more cautious wording would keep the conclusion firmly anchored in the data.

10. Data availability statements need to be aligned with PLOS requirements

This is an important issue for the journal. In the submission system, the authors appear to indicate that all data are fully available without restriction, but the detailed data statement says the data are not publicly available and may only be accessed upon request and approval. In the manuscript itself, the data availability statement says simply that the data are available from the corresponding author on reasonable request.

These statements are not consistent with one another and should be corrected before the paper can move forward.

11. A careful editorial cleanup is still needed

There are still a number of visible wording and formatting errors in the manuscript, for example:

*"between August 2022 and May 2024. with follow-up through May 2025" in the abstract,

*"suggesFted" in the introduction,

*and spacing problems such as "servicesrather."

Any attachments provided with reviews can be seen via the following link: [LINK]

--------------------------------------------------------- ---

General editorial requests:

(Note: not all will apply to your paper, but please check each item carefully)

*We ask every co-author listed on the manuscript to fill in a contributing author statement, making sure to declare all competing interests. If any of the co-authors have not filled in the statement, we will remind them to do so when the paper is revised. If all statements are not completed in a timely fashion this could hold up the re-review process. If new competing interests are declared later in the revision process, this may also hold up the submission. Should there be a problem getting one of your co-authors to fill in a statement we will be in contact. Please do not add or remove authors without first discussing this with the handling editor. You can see our competing interests policy here: http://journals.plos.org/plosmedicine/s/competing-interests.

*Please upload any figures associated with your paper as individual TIF or EPS files with 300dpi resolution at resubmission; please read our figure guidelines for more information on our requirements: http://journals.plos.org/plosmedicine/s/figures. While revising your submission, we strongly recommend that you use PLOS's NAAS tool (https://ngplosjournals.pagemajik.ai/artanalysis) to test your figure files. NAAS can convert your figure files to the TIFF file type and meet basic requirements (such as print size, resolution), or provide you with a report on issues that do not meet our requirements and that NAAS cannot fix.

After uploading your figures to PLOS's NAAS tool - https://ngplosjournals.pagemajik.ai/artanalysis, NAAS will process the files provided and display the results in the "Uploaded Files" section of the page as the processing is complete.

If the uploaded figures meet our requirements (or NAAS is able to fix the files to meet our requirements), the figure will be marked as "fixed" above. If NAAS is unable to fix the files, a red "failed" label will appear above.

When NAAS has confirmed that the figure files meet our requirements, please download the file via the download option, and include these NAAS processed figure files when submitting your revised manuscript.

*Please ensure that the paper adheres to the PLOS Data Availability Policy (see http://journals.plos.org/plosmedicine/s/data-availability), which requires that all data underlying the study's findings be provided in a repository or as Supporting Information. For data residing with a third party, authors are required to provide instructions with contact information (web or email address) for obtaining the data. Please note that a study author cannot be the contact person for the data. PLOS journals do not allow statements supported by "data not shown" or "unpublished results." For such statements, authors must provide supporting data or cite public sources that include it.

*We expect all researchers with submissions to PLOS in which author-generated code underpins the findings in the manuscript to make all author-generated code available without restrictions upon publication of the work. In cases where code is central to the manuscript, we may require the code to be made available as a condition of publication. Authors are responsible for ensuring that the code is reusable and well documented. Please make any custom code available, either as part of your data deposition or as a supplementary file. Please add a sentence to your data availability statement regarding any code used in the study, e.g. "The code used in the analysis is available from Github [URL] and archived in Zenodo [DOI link]" Please review our guidelines at https://journals.plos.org/plosmedicine/s/materials-software-and-code-sharing and ensure that your code is shared in a way that follows best practice and facilitates reproducibility and reuse. Because Github depositions can be readily changed or deleted, we encourage you to make a permanent DOI'd copy (e.g. in Zenodo) and provide the URL.

*Please provide a web address or email address for data access requests.

*COIs: Please declare membership of the PLOS Medicine Editorial Board where relevant.

*Please outline consent procedures (informed consent; written or oral) for participants in the iHEART-SA trial.

*Please ensure that the study is reported according to the STROBE guideline and include the completed checklist as Supporting Information.

FORMATTING - GENERAL

*Abstract: Please structure your abstract using the PLOS Medicine headings (Background, Methods and Findings, Conclusions). Please combine the Methods and Findings sections into one section.

*At this stage, we ask that you include a short, non-technical Author Summary of your research to make findings accessible to a wide audience that includes both scientists and non-scientists. The Author Summary should immediately follow the Abstract in your revised manuscript. This text is subject to editorial change and should be distinct from the scientific abstract. Ideally each sub-heading should contain 2-3 single sentence, concise bullet points containing the most salient points from your study. In the final bullet point of 'What Do These Findings Mean?', please include the main limitations of the study in non-technical language. Please see our author guidelines for more information: https://journals.plos.org/plosmedicine/s/revising-your-manuscript#loc-author-summary.

*Please express the main results with 95% CIs as well as p values. When reporting p values please report as p<0.001 and where higher as the exact p value p=0.002, for example. Throughout, suggest reporting statistical information as follows to improve clarity for the reader "22% (95% CI [13%,28%]; p</=)". Please be sure to define all numerical values at first use.

*Please include page numbers and line numbers in the manuscript file. Use continuous line numbers (do not restart the numbering on each page).

*Please cite the reference numbers in square brackets. Citations should precede punctuation.

FIGURES AND TABLES

*Please provide titles and legends for all figures and tables (including those in Supporting Information files).

*Please define all abbreviations used in each figure/table (including those in Supporting Information files).

*Please consider avoiding the use of red and green in order to make your figure more accessible to those with color blindness.

SUPPLEMENTARY MATERIAL

*Please note that supplementary material will be posted as supplied by the authors. Therefore, please amend it according to the relevant comments outlined here.

*Please cite your Supporting Information as outlined here: https://journals.plos.org/plosmedicine/s/supporting-information

REFERENCES

*PLOS uses the numbered citation (citation-sequence) method and first six authors, et al.

*Please ensure that journal name abbreviations match those found in the National Center for Biotechnology Information (NCBI) databases (http://www.ncbi.nlm.nih.gov/nlmcatalog/journals), and are appropriately formatted and capitalised.

*Where website addresses are cited, please include the complete URL and specify the date of access (e.g. [accessed: 12/06/2023]).

*Please also see https://journals.plos.org/plosmedicine/s/submission-guidelines#loc-references for further details on reference formatting.

STUDY TYPE-SPECIFIC REQUESTS - OBSERVATIONAL STUDIES

*Abstract: Please include the study design, population and setting, number of participants, years during which the study took place (enrollment and follow up), length of follow up, and main outcome measures.

*Please ensure that the study is reported according to the STROBE (or appropriate STROBE extension) guideline (available from: https://www.equator-network.org/reporting-guidelines/strobe) and include the completed STROBE (or STROBE extension) checklist as Supporting Information. Please add the following statement, or similar, to the Methods: "This study is reported as per the Strengthening the Reporting of Observational Studies in Epidemiology (STROBE) guideline (S1 Checklist)." When completing the checklist, please use section and paragraph numbers, rather than page numbers.

*For all observational studies, in the manuscript text, please indicate: (1) the specific hypotheses you intended to test, (2) the analytical methods by which you planned to test them, (3) the analyses you actually performed, and (4) when reported analyses differ from those that were planned, transparent explanations for differences that affect the reliability of the study's results. If a reported analysis was performed based on an interesting but unanticipated pattern in the data, please be clear that the analysis was data driven.

*Please state in the Methods section whether the study had a prospective protocol or analysis plan. If a prospective analysis plan (from your funding proposal, IRB or other ethics committee submission, study protocol, or other planning document written before analyzing the data) was used in designing the study, please include the relevant document(s) with your revised manuscript as a Supporting Information file to be published alongside your study and cite it in the Methods section. A legend for this file should be included at the end of your manuscript. If no such document exists, please make sure that the Methods section transparently describes when analyses were planned, and when/why any data-driven changes to analyses took place. Changes in the analysis, including those made in response to peer review comments, should be identified as such in the Methods section of the paper, with rationale.

# Author comment: Author response to Decision Letter 2

**DOI:** 10.1371/journal.pmed.1004957.r003

31 May 2026

Attachment Submitted filename: Gumede et al_Response to reviewers.pdf

# Peer review: Decision Letter 2

**DOI:** 10.1371/journal.pmed.1004957.r004

**Authors:** Alison Farrell

3 Aug 2026

Dear Dr. Gumede,

Thank you very much for re-submitting your manuscript "The longitudinal care cascade for hypertension: a clinic-based study of people with and without HIV in South Africa" (PMEDICINE-D-26-00436R2) for review by PLOS Medicine.

I have discussed the paper with my colleagues and it was also seen again by two reviewers. I am pleased to say that provided the remaining reviewer, editorial and production issues are dealt with we are planning to accept the paper for publication in the journal.

The remaining issues that need to be addressed are listed at the end of this email. Any accompanying reviewer attachments can be seen via the link below. Please take these into account before resubmitting your manuscript:

[LINK]

***Please note while forming your response, if your article is accepted, you may have the opportunity to make the peer review history publicly available. The record will include editor decision letters (with reviews) and your responses to reviewer comments. If eligible, we will contact you to opt in or out.***

In revising the manuscript for further consideration here, please ensure you address the specific points made by each reviewer and the editors. In your rebuttal letter you should indicate your response to the reviewers' and editors' comments and the changes you have made in the manuscript. Please submit a clean version of the paper as the main article file. A version with changes marked must also be uploaded as a marked up manuscript file.

Please also check the guidelines for revised papers at http://journals.plos.org/plosmedicine/s/revising-your-manuscript for any that apply to your paper. If you haven't already, we ask that you provide a short, non-technical Author Summary of your research to make findings accessible to a wide audience that includes both scientists and non-scientists. The Author Summary should immediately follow the Abstract in your revised manuscript. This text is subject to editorial change and should be distinct from the scientific abstract.

In addition to these revisions, you may need to complete some formatting changes, which you will receive in a follow up email. A member of our team will be in touch with a set of requests shortly. If you do not receive a separate email within a few days, please assume that checks have been completed, and no additional changes are required.

We expect to receive your revised manuscript within 1 week. Please email us (plosmedicine@plos.org) if you have any questions or concerns.

We ask every co-author listed on the manuscript to fill in a contributing author statement. If any of the co-authors have not filled in the statement, we will remind them to do so when the paper is revised. If all statements are not completed in a timely fashion this could hold up the re-review process. Should there be a problem getting one of your co-authors to fill in a statement we will be in contact. YOU MUST NOT ADD OR REMOVE AUTHORS UNLESS YOU HAVE ALERTED THE EDITOR HANDLING THE MANUSCRIPT TO THE CHANGE AND THEY SPECIFICALLY HAVE AGREED TO IT.

Please ensure that the paper adheres to the PLOS Data Availability Policy (see http://journals.plos.org/plosmedicine/s/data-availability), which requires that all data underlying the study's findings be provided in a repository or as Supporting Information. For data residing with a third party, authors are required to provide instructions with contact information for obtaining the data. PLOS journals do not allow statements supported by "data not shown" or "unpublished results." For such statements, authors must provide supporting data or cite public sources that include it.

To enhance the reproducibility of your results, we recommend that you deposit your laboratory protocols in protocols.io, where a protocol can be assigned its own identifier (DOI) such that it can be cited independently in the future. Additionally, PLOS ONE offers an option to publish peer-reviewed clinical study protocols. Read more information on sharing protocols at https://plos.org/protocols?utm_medium=editorial-email&utm_source=authorletters&utm_campaign=protocols

Please review your reference list to ensure that it is complete and correct. If you have cited papers that have been retracted, please include the rationale for doing so in the manuscript text, or remove these references and replace them with relevant current references. Any changes to the reference list should be mentioned in the rebuttal letter that accompanies your revised manuscript.

Please note, when your manuscript is accepted, an uncorrected proof of your manuscript will be published online ahead of the final version, unless you've already opted out via the online submission form. If, for any reason, you do not want an earlier version of your manuscript published online or are unsure if you have already indicated as such, please let the journal staff know immediately at plosmedicine@plos.org.

If you have any questions in the meantime, please contact me or the journal staff on plosmedicine@plos.org.

We look forward to receiving the revised manuscript by Aug 10 2026 11:59PM.

Sincerely,

Alison Farrell, PhD

Senior Editor

PLOS Medicine

plosmedicine.org

------------------------------------------------------------

Requests from Editors:

GENERAL EDITORIAL REQUESTS

* Your title must comply with PLOS Medicine's style. Your title must be nondeclarative and not a question. It should begin with main concept if possible. "Effect of" should be used only if causality can be inferred, i.e., for an RCT. Please revise your title to place the study design (only) ("A randomized controlled trial," "A retrospective study," "A modelling study," etc.) in the subtitle (ie, after a colon).

* Please confirm that your abstract complies with our requirements, including format (three sections: Background, Methods and Findings, and Conclusions) and providing all the information relevant to this study type https://journals.plos.org/plosmedicine/s/submission-guidelines#loc-abstract

* Please ensure that the Introduction ends with a clear description of the study question or hypothesis.

* Please ensure that all abbreviations are defined at first use throughout the text.

* Please confirm that all numbers presented in the abstract are present and identical to numbers presented in the main manuscript text.

GENERAL

* Please include a statement regarding use or non-use of AI tools and technologies at the end of the Methods. Please specify the name(s) and version(s) of any tool(s) used and a description of their use, including how the AI-assisted or generated content was evaluated by authors, and a clear statement of which aspects of the study, article contents, data, or supporting files were affected or generated by AI tool usage. Please also include a statement specifying who retains responsibility for the final manuscript. See our guidelines for more information: https://journals.plos.org/plosmedicine/s/ethical-publishing-practice#loc-artificial-intelligence-tools-and-technologies.

* Please remove the 'conclusions' subheading from the discussion. Please also remove any other subheadings from the discussion.

* In the last sentence of the Abstract Methods and Findings section, please describe the main limitation(s) of the study's methodology.

* In the author summary, in the final bullet point of 'What Do These Findings Mean?', please include the main limitations of the study in non-technical language.

FUNDING STATEMENT

* The funding statement should include: specific grant numbers, initials of authors who received each award, URLs to sponsors’ websites. Also, please state whether any sponsors or funders (other than the named authors) played any role in study design, data collection and analysis, the decision to publish, or preparation of the manuscript. If they had no role in the research, include this sentence: “The funders had no role in study design, data collection and analysis, decision to publish, or preparation of the manuscript.”

* Please ensure that the funding statement in the manuscript matches that in the metadata. They are presently discrepant.

COMPETING INTERESTS STATEMENT

* All authors must declare their relevant competing interests per the PLOS policy, which can be seen here: https://journals.plos.org/plosmedicine/s/competing-interests For authors with ties to industry, please indicate whether any of the interests has a financial stake in the results of the current study.

* Please add this statement to the manuscript's Competing Interests: "JMG is an Academic Editor on PLOS Medicine's editorial board.

DATA AVAILABILITY

* PLOS defines the “minimal data set” to consist of the data set used to reach the conclusions drawn in the manuscript with related metadata and methods, and any additional data required to replicate the reported study findings in their entirety. Authors do not need to submit their entire data set, or the raw data collected during an investigation. Please submit the following data:

The values behind the means, standard deviations and other measures reported;

The values used to build graphs;

The points extracted from images for analysis.

FIGURES

* Please provide titles and legends for all figures and tables (including those in Supporting Information files). Please define all acronyms used in each figure or table in its corresponding legend.

* Please provide the unadjusted comparisons as well as the adjusted comparisons in all relevant Tables

* Please specify the variables controlled for in all relevant Tables.

OBSERVATIONAL, COHORT, CROSS-SECTIONAL, AND CASE CONTROL STUDIES

* Please ensure that the study is reported according to the STROBE guideline, and include the completed STROBE checklist as Supporting Information. Please add the following statement, or similar, to the Methods: "This study is reported as per the Strengthening the Reporting of Observational Studies in Epidemiology (STROBE) guideline (S1 Checklist)."

The STROBE guideline can be found here: http://www.equator-network.org/reporting-guidelines/strobe/

When completing the checklist, please use section and paragraph numbers, rather than page numbers.

* Did your study have a prospective protocol or analysis plan? Please state this (either way) early in the Methods section.

a) If a prospective analysis plan (from your funding proposal, IRB or other ethics committee submission, study protocol, or other planning document written before analyzing the data) was used in designing the study, please include the relevant prospectively written document with your revised manuscript as a Supporting Information file to be published alongside your study, and cite it in the Methods section. A legend for this file should be included at the end of your manuscript.

b) If no such document exists, please make sure that the Methods section transparently describes when analyses were planned, and when/why any data-driven changes to analyses took place.

c) In either case, changes in the analysis-- including those made in response to peer review comments-- should be identified as such in the Methods section of the paper, with rationale.

* Your study is observational and therefore causality cannot be inferred. Please remove language that implies causality and refer to associations instead.

* For all observational studies, in the manuscript text, please indicate: (1) the specific hypotheses you intended to test, (2) the analytical methods by which you planned to test them, (3) the analyses you actually performed, and (4) when reported analyses differ from those that were planned, transparent explanations for differences that affect the reliability of the study's results. If a reported analysis was performed based on an interesting but unanticipated pattern in the data, please be clear that the analysis was data-driven.

Comments from Reviewers:

Reviewer #1: I thank the authors for the obvious care and effort they have put into replying to my review comments. I note the paper is much stronger this time around and is much closer to being publishable. That said, I feel there are a few items that still require attention primarily to address apparent inconsistencies and to improve interpretability. I have preserved the numbering from my original review for clarity and, where new items were required, I have added to the end of the prior numbering. If I have not referenced an item below, please consider it closed. All items are major unless noted otherwise.

4. Thank you for clarifying that participants were included in this study if they had baseline data and at least one follow-up. However, Table 2 indicated only ~66% of participants had one or more follow-up visits, suggesting patients with no follow up were included in the study. I suggest the authors remediate this inconsistency.

5. This is enormously improved. Well done!

6. I appreciate the authors intent on this point, but I could not find "S2 Table" in the submitted package. I note the inclusion of an Excel spreadsheet as "S1 Appendix" but found this difficult to read and understand. I suggest the authors provide a simple list of included hypertensive medications.

13. [MINOR] Again, this is much improved. The only thing I would flag is the relatively small number of clusters. Generally, 30-40 clusters are advisable to ensure validity of the robust method used by the authors. That said, in this case it is unlikely this would meaningfully impact the clinical conclusions presented. As such, I would suggest (but not insist) the authors very briefly note in the limitations that with only nine clusters the cluster-robust intervals may be modestly anticonservative, so borderline findings should be interpreted with care. The authors may wish to familiarise themselves with statistical tools (e.g., wild cluster bootstrap) that are valid for small cluster counts for situations at greater risk of anticonservative results such as cluster randomised trials.

22. I found Table 3 quite difficult to interpret. Part of the problem is in some columns aRRs > 1 are desirable whereas in other columns it's the opposite. Also, it is not at all clear what the denominators are and, accordingly, what the percentages mean. To assist with both of these issues, I suggest the authors make the numerator and denominator abundantly clear for each column, similar to what was presented in Figures 2 and 3. I would suggest this is prominent in the table (i.e., not just a table note) as this is essential to interpreting the results and is easily confused. Finally, I think the use of the word "events" in the column headings added to the confusion and I suggest it is dropped. This is because, again, in some cases the "event" is desirable and others undesirable.

23. There appear to be some inconsistencies with participant numbers across the manuscript, tables, and figures. I note the authors make a point of saying in a number of places that similar denominators vary due to completeness etc. but my concern is when ostensibly exactly the same descriptor is associated with differing participant counts. For example, number of participants with high BP in Table 2 is different to Figure 2, Figure 3 has different denominators between the second and third pairs of bars for the same descriptor "% of those treated and controlled at enrolment", and regressed to untreated counts seem to be inconsistent between Figure 3 and Table 3. I am sympathetic that there are a lot of counts being managed in this manuscript, but it is important that all the numbers with the same descriptor agree otherwise it undermines the presented research.

24. While the limitations para at line 483 is a good start, it misses a key point. Endpoint ascertainment is driven by visits, not follow-up time, and PNLHIV are more likely to have more visits and, accordingly, more progressions and regressions. Suggest this differential ascertainment is added as a limitation to that para.

25. [MINOR] It appears a para in the Discussion was included twice (starting at lines 483 and 492). Suggest deleting the duplicate.

Reviewer #2: The authors have appropriately responded to and addressed my comments.

There are minor proofreading issues for copyediting (for example, line 470-472 should be should be reviewed for clarity and adding a slash between and and or, "For example, BP measurements and HTN

diagnoses and treatment initiation may not always be recorded accurately or completely, and/or discontinuation of treatment." Also, a paragraph has been duplicated (lines 492-500 duplicate the paragraph immediately preceding paragraph).

However, I have no remaining substantive concerns and recommend the manuscript for publication.

Any attachments provided with reviews can be seen via the following link:

[LINK]

# Author comment: Author response to Decision Letter 3

**DOI:** 10.1371/journal.pmed.1004957.r005

14 Aug 2026

Attachment Submitted filename: Gumede et al_Response to reviewers R2.pdf

# Editor report: Decision Letter 3

**DOI:** 10.1371/journal.pmed.1004957.r006

**Authors:** Alison Farrell

20 Aug 2026

Dear Dr Gumede,

On behalf of my colleagues and the Academic Editor, Andre-Pascal Kengne, I am pleased to inform you that we have agreed to publish your manuscript "The longitudinal care cascade for hypertension: a clinic-based study of people with and without HIV in South Africa" (PMEDICINE-D-26-00436R3) in PLOS Medicine.

Before your manuscript can be formally accepted you will need to complete some formatting changes, which you will receive in a follow up email. Please be aware that it may take several days for you to receive this email; during this time no action is required by you. Once you have received these formatting requests, please note that your manuscript will not be scheduled for publication until you have made the required changes.

Please note that we ask you to revise your title to "The longitudinal care cascade for hypertension in people with and without HIV in South Africa: a clinic-based observational study".

In the meantime, please log into Editorial Manager at http://www.editorialmanager.com/pmedicine/, click the "Update My Information" link at the top of the page, and update your user information to ensure an efficient production process.

PRESS

We frequently collaborate with press offices. If your institution or institutions have a press office, please notify them about your upcoming paper at this point, to enable them to help maximise its impact. If the press office is planning to promote your findings, we would be grateful if they could coordinate with medicinepress@plos.org. If you have not yet opted out of the early version process, we ask that you notify us immediately of any press plans so that we may do so on your behalf.

We also ask that you take this opportunity to read our Embargo Policy regarding the discussion, promotion and media coverage of work that is yet to be published by PLOS. As your manuscript is not yet published, it is bound by the conditions of our Embargo Policy. Please be aware that this policy is in place both to ensure that any press coverage of your article is fully substantiated and to provide a direct link between such coverage and the published work. For full details of our Embargo Policy, please visit http://www.plos.org/about/media-inquiries/embargo-policy/.

To enhance the reproducibility of your results, we recommend that you deposit your laboratory protocols in protocols.io, where a protocol can be assigned its own identifier (DOI) such that it can be cited independently in the future. Additionally, PLOS ONE offers an option to publish peer-reviewed clinical study protocols. Read more information on sharing protocols at https://plos.org/protocols?utm_medium=editorial-email&utm_source=authorletters&utm_campaign=protocols

Thank you again for submitting to PLOS Medicine. We look forward to publishing your paper.

Sincerely,

Alison Farrell, Ph.D.

Senior Editor

PLOS Medicine
